# Solid-Phase Reactivity-Directed Extraction (SPREx): An Alternative Approach for Simultaneous Extraction, Identification, and Prioritization of Toxic Electrophiles Produced in Water Treatment Applications

**DOI:** 10.1021/acsenvironau.4c00025

**Published:** 2024-09-20

**Authors:** Daisy N. Grace, Matthew N. Newmeyer, Carsten Prasse

**Affiliations:** † Department of Environmental Health and Engineering, Johns Hopkins University, Baltimore, Maryland 21218, United States; ‡ Risk Sciences and Policy Institute, Bloomberg School of Public Health, Johns Hopkins University, Baltimore, Maryland 21205, United States

**Keywords:** reactivity-directed analysis, oxidation byproducts, disinfection byproducts, organic electrophiles, carbonyls, extraction, *in chemico*

## Abstract

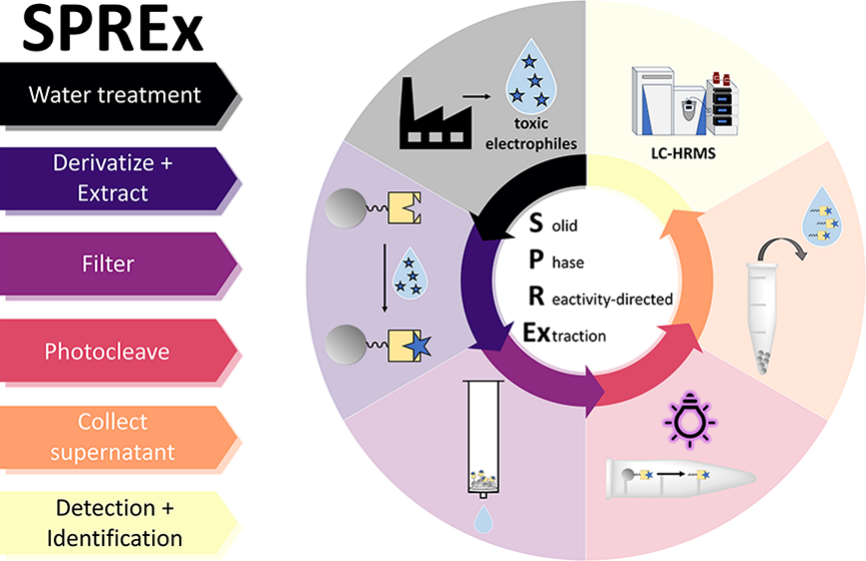

Current strategies to assess water quality are ineffective at prioritizing the most toxic chemicals within a treated water sample. Although it is well known that oxidation byproducts (OBPs) from water treatment processes (e.g., chlorination and ozonation) are linked to adverse health outcomes such as skin diseases, reproductive toxicity, and various cancers, we are still unable to account for a large fraction of the toxicity drivers. Previous approaches utilize *in vitro* or *in vivo* assays to assess OBPs on an individual basis, which is too time- and resource-intensive considering the countless number of transformation byproducts of unknown toxicities that exist in treated waters. *In vitro* assays have also been developed to analyze the toxicity of OBPs in environmental mixtures, but these approaches do not provide identification information about the responsible toxicants. Furthermore, an additional challenge for OBP detection arises during the extraction and detection stages of analysis, as certain OBPs are typically lost using traditional extraction methods or are not detectable via liquid-chromatography–high-resolution mass spectrometry (LC-HRMS) without derivatization. To address these issues, we have developed the analytical assay **S**olid-**P**hase **R**eactivity-directed **Ex**traction (SPREx), which aims to provide an all-in-one evaluation for (i) *in chemico* toxicity screening, (ii) extraction, (iii) detection, and (iv) identification via LC-HRMS. The performance of SPREx was evaluated by testing different nucleophile probes for the capture and detection of 24 different carbonyl compounds, which serve as model electrophiles and are known OBPs that provide unique extraction and detection challenges. SPREx provided distinct advantages for extraction recoveries and was an effective screening tool for carbonyl detection and quantification in complex water matrices such as drinking water and wastewater.

## Introduction

Current approaches for assessing toxic oxidation byproduct (OBP) formation overlook a substantial number of OBPs that have been identified to date or fail to prioritize the relevant toxicity drivers, leaving many OBPs unmonitored and unregulated. It is well known that oxidation byproducts—which are formed during reactions between organic matter and oxidants (e.g., chlorine, ozone, hydroxyl radicals) in water treatment plants—have been linked to adverse health outcomes.^1−6^ Ozonation, which is widely used for both disinfection and micropollutant abatement, generates carbonyl OBPs^4,7−14^ that are known to be carcinogenic, mutagenic, teratogenic, and neurotoxic and can cause reproductive toxicity.^15−24^ Chlorine and chloramine are routinely utilized for drinking water disinfection but also react with organic matter to produce OBPs.^10,25−27^ Exposures to these OBPs are linked to adverse health outcomes such as skin disease, reproductive toxicity, and various cancers in epidemiologic studies.^1,6,28^ However, our methods for evaluating and regulating OBPs lag far behind our knowledge regarding their abundance, variety, and toxicity.^29,30^ For instance, while over 700 OBPs have been detected, only 11 are currently regulated by the EPA, and these 11 are not the most toxic of those identified.^1,28,31−35^ An additional challenge is that the water composition (e.g., organic matter content, bromine/iodine concentrations) as well as the oxidant type and dose strongly influence OBP formation, all of which are likely to vary across water treatment plants, possibly producing widely different OBP concentrations at different sites.^26,27,34−42^

Modern toxicity assessments for OBPs often involve time-consuming *in vivo* or *in vitro* assays that analyze either: 1) toxicity of *known* OBPs on an individual, chemical-by-chemical basis, thus ignoring environmentally relevant mixtures and unknowns^5,33,43−51^ or 2) overall toxicity of a treated water sample, without identifying the responsible toxicant(s).^26,52−59^ The former suffers from the lack of available chemical standards for many identified OBPs, as most of them remain unknown. The latter highlights the shortcoming that treatment solutions cannot be informed if the toxicant identities remain unknown. It is not feasible to continue using the current approaches to assess the tens of thousands of anthropogenic chemicals of concern in addition to their countless associated transformation products; otherwise, the knowledge gap between approaches to identify OBPs and determine which are of the highest toxicological concern will remain substantial. Therefore, all-encompassing methods are needed to achieve simultaneous: (i) high-throughput analysis of unknown mixtures, (ii) identification of individual toxicants, and (iii) prioritization of analytes that are of highest environmental and human health concern.

To achieve higher throughput compared to *in vitro/in vivo* assays, *in chemico* assays have been more recently applied to study OBP formation and toxicity. Drawing from insights in molecular toxicology, *in chemico* assays utilize molecular probes that mimic the biological targets of OBPs.^43^ OBPs are electrophilic by nature and react with electron-rich nucleophiles to form covalent bonds, which is the underlying reason for their toxicity.^15,60,61^ Biologically, these nucleophiles are typically present as amines (e.g., proteins via lysine/arginine and DNA bases) and thiols (e.g., glutathione and proteins via cysteine).^60,62−67^ When toxic electrophiles bind to these nucleophiles, they disrupt their typical structure and function, which is the molecular initiating event that cascades into an adverse health outcome.^15,60^ In molecular toxicology, glutathione depletion assays have been successfully used to evaluate aquatic toxicity of organic electrophiles such as carbonyls.^68−71^ Moreover, peptide and amino acid depletion assays such as the direct peptide reactivity assay (DPRA) and the amino acid derivative reactivity assay (ADRA) have been endorsed by industry for their high-throughput analysis of electrophilic skin sensitizers.^43,72−78^ Particular to OBP assessment, *in chemico* assays have included cysteine, glutathione, or lysine probes to evaluate individual OBP toxicity,^79^ evaluate whole-sample toxicity of tap waters and wastewaters,^27,80,81^ or provide identification of individual OBPs.^82−86^ While these *in chemico* assessments have provided advantages for electrophilic OBP assessment, there remains a key drawback: many OBPs in the environment are present at low concentrations (nanomolar to low micromolar), so extraction procedures have been utilized previously to concentrate the analyte(s) of interest prior to detection. However, many OBPs are reactive, polar, and/or volatile and do not survive traditional extraction procedures such as solid-phase extraction (SPE).^83,87−90^ Therefore, common extraction procedures such as SPE may not be suitable for detecting a large fraction of the toxicity drivers in treated water samples, as many of them are likely polar analytes that are not retained on conventional SPE sorbents.^87−89^

Therefore, to address the shortcomings of current OBP assessments, this study introduces a novel extraction approach that specifically targets the detection of toxic organic electrophiles at environmentally relevant concentrations in water. This approach, which we call **S**olid-**P**hase **R**eactivity-directed **Ex**traction (SPREx), combines approaches from both molecular toxicology and analytical chemistry to provide key advantages for high-throughput OBP: (i) toxicity prioritization, (ii) extraction, (iii) detection, and (iv) identification. To demonstrate the applicability of the SPREx approach, this study focuses on the detection of carbonyls, which serve as model electrophiles and are commonly found in water treated by various chemical oxidants such as chlorine, ozone, and hydroxyl radicals.^10,84^

## Materials and Methods

### Overview of SPREx

**Figure 1:**
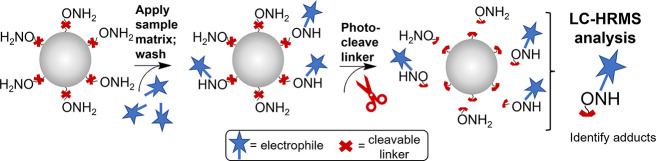
Overview of the SPREx assay. Nucleophiles (e.g., −ONH_2_) are immobilized onto solid-phase microbeads. Electrophiles (blue stars; OBPs) form adducts with the beads, and the rest of the sample matrix is removed to promote a higher analytical sensitivity. The nucleophile–electrophile adducts are then cleaved from the beads to enable detection and identification via LC-HRMS.

The basis of SPREx combines the high-throughput advantages of an *in chemico* toxicology assay while enabling extraction by utilizing a solid-phase nucleophile probe to capture, detect, and identify (toxic) electrophiles (Figure 1).

#### Toxicity Screening

SPREx toxicity screening relies on an *in chemico* approach to prioritize toxic analytes within complex mixtures. Known as reactivity-directed analysis, this approach involves developing probes that mimic the biological targets of toxicants for their capture and detection.^43^ For reactivity-directed analysis, rather than viewing these nucleophiles as biological targets to enact electrophilic OBP toxicity, they are instead developed into a detection probe to specifically detect electrophilic analytes.

#### Extraction

In SPREx, the nucleophilic detection probes are immobilized onto solid-phase microbeads, which enables a built-in extraction feature to provide key advantages over previous extraction technologies for OBP analysis (Figure 1). With SPREx, the stable adduct formation of the electrophilic OBPs onto the immobilized nucleophiles increases the molecular weight while decreasing the reactivity and volatility of the overall analyte prior to liquid chromatography-high-resolution mass spectrometry (LC-HRMS) analysis.^91^ Once the beads have reacted, they only require a simple filtration step to wash out the rest of the sample matrix, thus enabling compound concentration and specific extraction of only the electrophilic analytes bound to the beads. As mentioned before, SPREx is particularly advantageous for the detection of polar electrophilic OBPs, which are difficult to extract with conventional approaches such as solid-phase extraction (SPE).^87,90^ In addition, the extraction procedure of SPREx enables a wide range of matrices to be utilized during the initial reaction step, so high concentrations of salts/buffers can be used and filtered out before LC-HRMS analysis, thus reducing ion suppression during electrospray ionization and issues related to instrument contamination. Matrix effects in the final sample caused by other organic molecules (e.g., organic components from various environmental water sources) should also be minimized or negligible due to the filtration step in SPREx prior to LC-HRMS analysis.

#### Detection

In addition to functioning as a toxicity screening tool, derivatization with the nucleophiles serves an additional purpose in electrophilic OBP detection. Certain OBPs (e.g., carbonyls) are poorly ionizable and have low molecular weights, making them undetectable in LC-HRMS. Derivatizing with an ionizable nucleophile probe both increases the molecular weight of the analyte while also enabling overall adduct detection.^91^

#### Identification

While solid-phase microbead nucleophile probes enable sample extraction, they cannot be directly analyzed by LC-HRMS. Therefore, cleavable linkers (Figure 1, red “X”) enable the separation of the nucleophile–electrophile adducts from the beads to analyze via LC-HRMS. With the molecular formula of the cleaved nucleophile known, this formula can be subtracted from the detected adduct mass to identify the molecular formulas of unknown electrophiles. This cleavable bead approach has been successfully utilized for the assessment of the skin sensitization of individual chemicals but has never been applied for the assessment of complex chemical mixtures such as those found in environmental samples.^74,75^

### Standards and Reagents

Detailed descriptions for the chemicals used in this work are provided in Supporting Information 1 (S1) in Text S1.1 and Table S1.1.

### Microbead Synthesis

Because nucleophile-labeled, cleavable beads are not commercially available, they were synthesized via copper-catalyzed azide–alkyne cycloaddition (CuAAC) click chemistry reactions between alkyne-functionalized beads with cleavable linkers and an azide-labeled nucleophile.^92−94^ Both photocleavable (PC) or chemically cleavable (Dde) alkyne agarose beads were evaluated in this study, where photocleavage is performed with 365 nm light, while Dde is 1-(4,4-dimethyl-2,6-dioxocyclohex-1-ylidene)ethyl) and is cleaved via 2% hydrazine. The click chemistry reaction between the alkyne and azide produces high yields of a 1,2,3-triazole, thus forming an irreversible bond connecting the nucleophile probe with the cleavable microbead (see Figure S1.1).^92−94^ The complete methods for the cleavable microbead synthesis are detailed in Text S1.2.

### Microbead Nucleophile Quantification

For carbonyl detection, an excess concentration of the nucleophile must be present relative to the carbonyl to ensure complete derivatization. After the nucleophile-labeled, cleavable microbeads were synthesized (herein referred to as the nucleophile beads), the nucleophile concentration on the surface of the beads was initially unknown. To address this issue, we determined the nucleophile concentrations via colorimetric techniques, where the bicinchoninic assay (BCA/Cu^+^ assay) was performed to measure the amine or thiol concentrations on the bead surfaces (for details, see Text S1.3).

### Nucleophile Probe Testing

To determine the best nucleophile probe and pH conditions for carbonyl derivatization, different nucleophilic chemical functional groups were tested against a set of 16 different carbonyl compounds ranging in sterics, polarity, and functional groups (i.e., aldehyde, ketone, amide, ester, saturated (di)carbonyls, and α,β-unsaturated (di)carbonyls). The tested nucleophile probes consisted of the following: −NH_2_, −SH, −NNH_2_, and −ONH_2_ (see Figure S1.4 for full structures of all synthesized beads). According to the hard and soft acid and base theory, these nucleophiles demonstrate varying degrees of hardness/softness, which may impact their reactivity between different carbonyls, as hard nucleophiles prefer reactions with hard electrophiles (e.g., the carbonyl carbon), whereas soft nucleophiles prefer reactions with soft electrophiles (e.g., the β-carbon in α,β-unsaturated carbonyls).^15,60^ The carbonyls consisted of 1,4-benzoquinone, 2,6-dichloro-1,4-benzoquinone, 2-butene-1,4-dial, 2-methacrolein, 3-methylcrotonaldehyde, acrolein, acrylamide, benzaldehyde, butanal, crotonaldehyde, furaldehyde, glyoxal, hexanal, methacrylate, methyl vinyl ketone, and methylglyoxal (see Table S1.1 for all molecular structures). A stock containing 100 mM of each carbonyl was made daily in chilled methanol using a gastight syringe to avoid loss from volatilization and further diluted in water. The various conditions tested with these bead systems are summarized in Table 1.

**Table 1:** Nucleophile Bead Systems Tested with the Carbonyl Mixture^a^

**nucleophile probe**	**cleavable linker**	**pH**	**reducing agent**
amine	Dde	7	none
amine	Dde	9	none
amine	PC	7	none
amine	PC	7	sodium cyanoborohydride
amine	PC	9	none
amine	PC	9	sodium cyanoborohydride
amine	PC	9	sodium triacetoxyborohydride
thiol	PC	9	none
thiol	PC	9	TCEP
hydrazide	PC	5	none
hydrazide	PC	7	none
aminooxy	PC	5	none
aminooxy	PC	7	none
aminooxy	PC	9	none

a PC = photocleavable, Dde = 1-(4,4-dimethyl-2,6-dioxocyclohex-1-ylidene)ethyl.

Each condition was tested in triplicate using 300 μL of the bead stock and 5 μM of each carbonyl in 10 mM phosphate buffer (final volume = 1.5 mL). For applicable conditions (specified in Table 1), 1 mM sodium cyanoborohydride, sodium triacetoxyborohydride, or tris(2-carboxyethyl)phosphine (TCEP) was added. The borohydrides are utilized as reducing agents to aid in the stability of amine-carbonyl adducts,^91,95^ while TCEP aids in the reduction of disulfide bonds for thiol-carbonyl analysis (see the Results and Discussion section for further details).^96^ The pH values were selected to test (i) conditions similar to those at water treatment plants (pH 7), (ii) if more basic conditions (pH 9) enhanced the nucleophilicity of the thiol or amines to enhance carbonyl reactivity, or (iii) if more acidic conditions (pH 5) catalyzed carbonyl adduct formation, the latter of which has been observed in previous carbonyl derivatization studies.^97^ The samples were allowed to react overnight with shaking before centrifuging at 8000 × *g* for 5 min, where the supernatant was removed and refilled with 1.5 mL of Milli-Q water. This wash step was repeated five times to remove the rest of the sample matrix (i.e., buffer, reducing agents, or unreacted carbonyls), leaving behind the bead-immobilized carbonyl-nucleophile adducts. For the Dde-bead samples, 1.5 mL of a 2% hydrazine solution was added back to the beads and allowed to react with shaking for 50 min to cleave the carbonyl-nucleophile adducts into solution.^98^ For the PC-bead samples, 1.5 mL of Milli-Q water was added back to the beads and photocleaved for 20 min using a 365 nm UV light. Then, all samples were centrifuged at 8000 × *g* for 5 min, and the supernatant was collected. As an added precaution to prevent microbeads from passing into the LC-HRMS instrument, the supernatant was sent through a 0.22 μm PTFE filter into an HPLC vial for analysis. These steps were repeated for triplicates of controls for each of the sample sets; these controls contained all of the same reagents except for the spiked carbonyls and were used for background subtraction (i.e., subtracting the peak area detected in the control from the peak area detected in the sample).

### Recovery Experiments

Since the built-in reactivity-directed extraction feature of SPREx is a key advantage over conventional SPE-based extraction methods for carbonyl analysis, recovery experiments were conducted to determine if—and to what extent—loss of carbonyls occurred during the extraction process. For these experiments, aminooxy-PC beads were selected for carbonyl derivatization, as they provided the highest LC-HRMS signals for the majority of the carbonyl-nucleophile adducts compared to the other bead systems that were tested (see Results and Discussion: Nucleophile Probe Testing for further details). All carbonyls described in Nucleophile Probe Testing were used in these experiments except for the two quinones and acrylamide, which were unreactive or produced poor peak shapes under the chromatographic conditions.

To test the recovery of the carbonyls undergoing SPREx, 300 μL of beads were added in 10 mM pH 7 phosphate buffer. Milli-Q water was added to reach a final volume of 10 mL in amber vials with no headspace. Then, 100 μL of a concentrated carbonyl mixture was spiked into the vials to achieve a final concentration of 0.1, 1, or 5 μM (each tested in triplicate). The vials were then capped and allowed to react overnight with shaking at 250 rpm at 25 °C. After reacting, the bead solution was sent through an empty 3 mL SPE cartridge fitted with a 20 μm filter to collect the beads, while the rest of the sample was discarded. The beads were collected by pushing the filters to the tops of the cartridges and rinsing the beads at the tops of the filters with 1.5 mL of Milli-Q water in a 1.5 mL Eppendorf tube. The beads were then photocleaved at 365 nm for 20 min with a UV lamp, and the tubes were subsequently centrifuged at 8000 × *g* for 5 min. The supernatant was collected and passed through a 0.22 μm PTFE filter and analyzed via LC-HRMS (Figure S1.5).

In order to calculate the absolute recoveries, aqueous samples not undergoing SPREx were prepared to compare to the extracted samples described above. Because there is no commercially available standard of the free nucleophile probe, a stock solution of the cleaved nucleophile probe first had to be generated by exposing 300 μL of bead stock in 1.2 mL of Milli-Q water to 365 nm light for 20 min with a UV lamp in a 1.5 mL Eppendorf tube. The tubes were then centrifuged at 8000 × *g* for 5 min before collecting the supernatant and passing it through a 0.22 μm PTFE filter. Then, this generated nucleophile stock was added to 10 mM phosphate buffer and 0.1, 1, or 5 μM of the carbonyl mix in a 1.5 mL Eppendorf tube, shaken overnight at 250 rpm at 25 °C, and analyzed on the LC-HRMS. The recovery for each carbonyl is defined in eq 1. It should be noted that this approach only provides an estimate of the recoveries, as the nucleophiles for the carbonyl reactions are not an exact match in the extracted versus nonextracted samples (discussed in further detail in the Results and Discussion section).
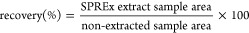
1

### Accuracy Experiments

To test whether carbonyl concentrations could be accurately quantified using the SPREx assay, accuracy experiments were conducted (Figure S1.6). Aminooxy beads (300 μL) were reacted in pH 7 phosphate buffer in 10 mL amber vials with no headspace (final volume = 10 mL) with 0 (negative control), 0.05, 0.1, 1, or 10 μM of carbonyl mix and 10 μM each of internal standards butanal-d2 and benzaldehyde-d5 in triplicate. The incorporation of internal standards allows us to account for extraction losses and/or potential matrix effects in the subsequent LC-HRMS analysis. Due to the lack of commercially available isotope-labeled nucleophile probe and isotope-labeled carbonyls, only benzaldehyde-d5 and butanal-d2 were included in this study as internal standards. Similar to previous work regarding aldehyde detection, benzaldehyde-d5 was used as the internal standard for all carbonyl adducts,^7,8^ except for butanal and hexanal, for which butanal-d2 was utilized due to better similarities in structure and chromatographic behavior compared to benzaldehyde-d5 (Figures S2.2–37 for structures and discussion below in section Nucleophile Probe Testing). The microbead samples spiked with the known carbonyl concentrations are herein referred to as “spiked controls.” A calibration curve was also prepared under the same conditions with carbonyl mix (0.01, 0.02, 0.05, 0.1, 0.5, 0.8, 1, 2, 5, 8, and 10 μM of each carbonyl) and 10 μM each of both internal standards.

All samples and calibrators were extracted as described above for the recovery samples, and the filtered supernatant was analyzed via LC-HRMS for the carbonyl-aminooxy labeled adducts. The carbonyl concentrations in the spiked controls were quantified based on the calibration curves (Figure S1.7). High background signals are known to be present in negative controls, which is a common challenge encountered during carbonyl quantification.^8,9,99,100^ Due to the high signal in the negative controls for the majority of the carbonyl-nucleophile adducts, both the calibration curve and all samples were background subtracted by the negative controls.^7,8^ The limit of detection was defined as the lowest calibrant with a normalized peak area distinguishable from that of the negative control. The limit of quantification was the lowest spiked concentration that could be accurately quantified (70–130% accuracy). More details on determining the detection and quantification limits are provided in Text S1.4. Accuracy was defined using eq 2.
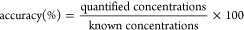
2

### Stability Experiments

To detect and accurately quantify carbonyls via LC-HRMS, the nucleophile-carbonyl adduct must be stable since nonderivatized carbonyls themselves are not LC-HRMS detectable.^91^ Therefore, to ensure that the SPREx nucleophile probes yielded stable carbonyl adducts, stability experiments were conducted. Target carbonyls were spiked at four different concentrations (0.05, 0.1, 1, and 10 μM) along with 10 μM each of internal standards butanal-d2 and benzaldehyde-d5 to 300 μL of aminooxy beads at pH 7 in triplicate and extracted with the method discussed above (final volume = 10 mL with no headspace in amber vials). The carbonyl concentrations in the spiked controls were quantified, as described above. One of the triplicate spiked controls at each concentration was injected at the beginning, middle, and end of a ∼72 h LC-HRMS analysis to observe if the quantification accuracy decreased over the duration of the total run time.

### Applications of SPREx

The application of SPREx was tested for the detection of carbonyls formed during the chlorination of amino acids (AAs) and the quantification of carbonyls in drinking water and wastewater. AAs were selected as model compounds since they are known precursors of odorous aldehydes, relevant in environmental waters, and especially abundant in wastewater and water sources with algae contamination.^101−112^

#### Amino Acid Chlorination

Nine out of the 20 AAs (l-alanine, l-glycine, l-isoleucine, l-leucine, l-methionine, l-phenylalanine, l-serine, l-threonine, and l-valine—see structures in Table S1.4) were chosen based on the availability of a commercial carbonyl standard (determined based on literature of the expected carbonyl formation mechanisms; Table S1.4 shows the expected carbonyl transformation product from each AA system).^102,103,107^ For the AA chlorination experiments, 10 μM of individual AAs were reacted with sodium hypochlorite in 10 mM pH 7 phosphate buffer to achieve a final free chlorine dose of 1.5× AA concentration. This ratio was chosen based on previous literature for achieving maximum aldehyde concentrations.^103^ A separate set of controls was prepared under the same conditions but (1) with AA, without chlorine (for a 0 Cl:AA dose); (2) with chlorine, without AA (for background subtraction from the 1.5 Cl:AA samples); and (3) without AA or chlorine (negative control) for subtraction from the 0 Cl:AA samples. These reactions were conducted in capped 60 mL amber vials with no headspace, vortexed periodically, and allowed to react for 2 h before quenching with 150 μM sodium thiosulfate for at least 25 min. Afterward, the solution was transferred into three 10 mL amber vials (for triplicate samples of each AA) containing 300 μL of aminooxy-PC beads. A set of spiked controls (0.05, 0.1, 1, and 10 μM) for the AA-related carbonyls was also prepared in triplicate to evaluate the quantification accuracy and stability over the course of the LC-HRMS analysis using the procedures described above. More information on calibration curves and discussion on detection limitations for the AA carbonyls can be found in Figure S1.7 and Text S1.4.

#### Quantification in Drinking Water and Wastewater

Wastewater effluent was collected at a treatment plant in October 2023 and stored at 4 °C until analysis. The wastewater samples were filtered first through a 0.45 μm PVDF filter and then through a 0.2 μm PES filter. The total organic carbon (TOC) concentration was measured via a Shimadzu Total Organic Carbon Analyzer (TOC-L_CPH/CPN_ Series) and determined to be 5.3 ppm. Drinking water was obtained from a drinking fountain that provided water treated by the Baltimore City Department of Public Works and prepared for analysis on the same day. Any residual chlorine was quenched with 150 μM sodium thiosulfate before spiking in known concentrations of carbonyls. All water samples contained 10 mM pH 7 phosphate buffer to maintain a consistent pH throughout all water matrices analyzed. All samples were prepared in triplicate in 10 mL amber vials containing 300 μL of aminooxy beads (final volume = 10 mL with no headspace). Spiked controls and calibration curves of the AA-related carbonyls were prepared in Milli-Q water, drinking water, and wastewater to check the accuracy of the carbonyl quantification in increasingly complex environmental matrices, respectively, using the methods described above.

### LC-HRMS Analysis

The details on LC-HRMS analysis of carbonyl-nucleophile adducts can be found in Text S1.5. Briefly, an UltiMate 3000 RSLCnano ultrahigh-performance liquid chromatography system coupled with a Thermo Scientific Q Exactive HF high-resolution mass spectrometer (LC-HRMS) equipped with a heated-electrospray ionization (H-ESI) source was utilized for the detection of all derivatized carbonyl compounds in positive ionization mode (Text S1.5). For the LC system, separations were performed with a Phenomenex Synergi Hydro-RP column (4 μm, 80 Å, 1 × 150 mm) held at 30 °C. For the mobile phases, 0.1% (v/v) formic acid in Milli-Q water (A) and methanol (B) were utilized.

## Results and Discussion

### Microbead Nucleophile Quantification

The nucleophile concentration on the synthesized microbeads was evaluated to select the appropriate volume of beads to be used in subsequent experiments. This volume of bead stock was selected to maintain an excess nucleophile concentration relative to electrophiles to ensure complete derivatization. From the BCA/Cu^+^ assay, 150 μL of thiol bead stock was found to contain 102 ± 9 μM thiol (average ± standard deviation; *n* = 3), while the amine bead system contained 121 ± 3 μM amine (average ± standard deviation; *n* = 3). The lower nucleophile concentration of the thiol bead system relative to the amines can potentially be attributed to disulfide bond formation (e.g., free nucleophile must be present to reduce copper(II) in the BCA/Cu^+^ assay). The external calibration curves of the known thiol concentrations in the BCA/Cu^+^ assay can be found in Figure S1.3. Overall, these concentrations showed that the CuAAC click chemistry reactions to synthesize the nucleophile, cleavable microbeads were effective. With this knowledge, 300 μL of beads were chosen for subsequent experiments to ensure a balance between (1) excess nucleophile concentrations for carbonyl derivatization and (2) minimization of bead waste.

### Nucleophile Probe Testing

Various nucleophile probe conditions were tested to identify the probe that would enable (1) effective cleavage of carbonyl adducts from solid-phase microbeads for LC-HRMS analysis, (2) sufficient stability of carbonyl-nucleophile adducts for reliable carbonyl detection, and (3) high signals in LC-HRMS analysis to effectively evaluate a wide variety of carbonyls at low concentrations. Addressing the first goal, it was determined that the PC linker outperformed the Dde cleavable linker and was used for all subsequent analyses (for a detailed discussion, see Text S1.6). Addressing goals (2) and (3), different nucleophiles were evaluated to determine which produced the highest LC-HRMS signals. These nucleophiles consisted of −SH, −NH_2_, reduced −NH_2_, −NNH_2_, and −ONH_2_. Thiols (−SH) are toxicologically meaningful *in chemico* probes due to their presence in biomolecules such as cysteine as well as the antioxidant glutathione.^62,71,80^ However, the −SH bead system did not perform as well as the −ONH_2_ beads for effective carbonyl derivatization and detection. Further discussion can be found in Text S1.7. The −NH_2_ moiety is also a toxicologically meaningful candidate to use as an *in chemico* toxicity probe in SPREx. Lysine and DNA bases are abundant with primary amine groups, and these nucleophilic biomolecules serve as targets for electrophiles in the molecular initiating events that cascade into adverse health outcomes.^15,60^ As a derivatization agent, however, the −NH_2_ probe alone can be ineffective in aqueous conditions due to the reversibility of the amine-carbonyl products (known as Schiff bases or imines) when water interacts with the imine’s carbon–nitrogen double bond (Figure S1.8).^91^ This was observed in the LC-HRMS analysis where all but two PC-amine bead carbonyl adducts were not detected, and for the few adducts that were detected, peaks had low intensities or poor shapes. Incorporating a reducing agent enabled the detection of three more carbonyl adducts, albeit with low intensities. A full discussion on the −NH_2_ and reduced −NH_2_ system results is provided in Text S1.8.

#### Hydrazide and Aminooxy Probes

Since the instability of the Schiff base adduct presents an issue for reliable carbonyl detection—and reducing agents did not drastically improve the carbonyl adduct signals—two alternative nucleophile systems were tested: hydrazide (−NNH_2_) and aminooxy (−ONH_2_). The heteroatom N or O adjacent to the amine group can help donate electron density into the imine product, decreasing the partial positive charge on the imine carbon and making it less favorable for nucleophilic attack from water molecules (i.e., decreasing the favorability for the reverse reaction).^113^ The increased electron density around the amine group can also make it a stronger nucleophile for nucleophilic attack on the carbonyls. For these reasons, −NNH_2_ nucleophile derivatization reagents have been previously utilized by using 2,4-dinitrophenylhydrazine (DNPH)^11,99^ and, more recently, *p*-toluenesulfonyl hydrazide (TSH)^7−9^ to detect carbonyls in disinfected waters. Derivatization reagents using −ONH_2_ as the nucleophilic probe have also been utilized in GC-MS for carbonyl detection (e.g., *o*-(2,3,4,5,6-pentafluorobenzyl)-hydroxylamine (PFBOA)).^100^ Previous research has indicated that −ONH_2_ Schiff base products have a slight stability advantage over −NNH_2_ Schiff bases,^113^ so both bead systems were compared in the SPREx assay to determine which provided better signals in LC-HRMS analysis.

**Figure 2:**
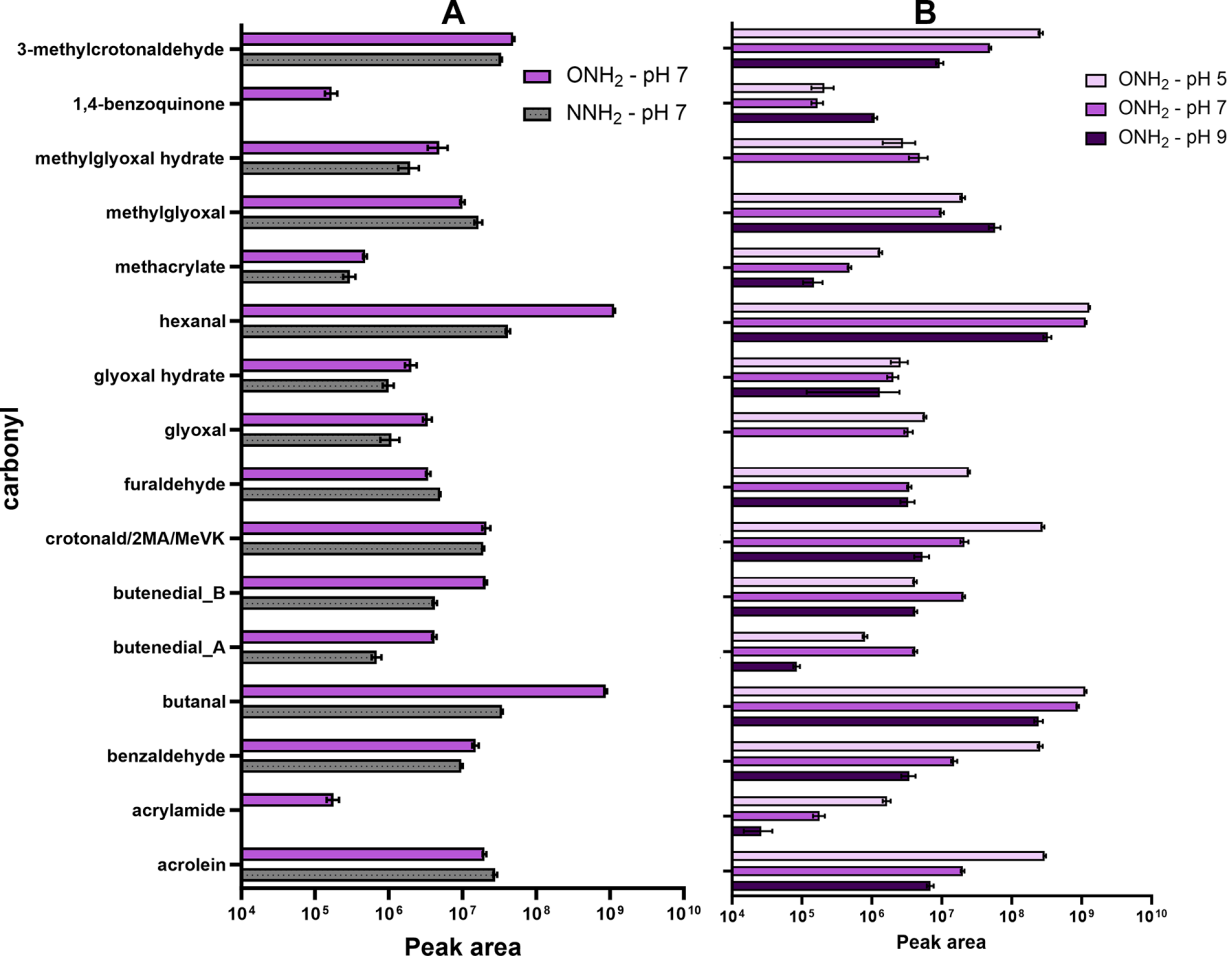
(A) (left) Peak areas (log_10_ scale) for selected carbonyl Schiff base adducts detected in the −ONH_2_ bead system (purple; top) and −NNH_2_ bead system (gray; bottom) reacted at pH 7. A figure depicting the Michael addition adducts for α,β-unsaturated carbonyls is available in the SI (Figure S1.10). (B) (right) pH impact on carbonyl adduct peak areas (log_10_ scale) in the ONH_2_ bead system for selected carbonyls. Michael addition adducts for α,β-unsaturated carbonyls can be found in the Supporting Information (Figure S1.12). Butenedial is depicted twice, as two chromatographically resolved peaks were detected (Figures S2.3–S4). Two products each are also depicted for dicarbonyls glyoxal and methylglyoxal, as hydrate formation at the second carbonyl was also observed. Values are presented as the average of triplicates, with error bars indicating the standard deviation.

To evaluate the −ONH_2_ versus −NNH_2_ nucleophile probes for forming stable carbonyl derivatives, the peak areas were compared at both pH 5 and 7. Overall, both probes provided improved stability compared with the reduced amine bead system, with the −ONH_2_ beads providing greater signals for certain carbonyls. A summary of the full results for the −ONH_2_ versus −NNH_2_ comparisons at pH 5 or 7 can be found in Figures S1.9–12. A summary of selected carbonyls detected via −ONH_2_ versus −NNH_2_ at pH 7 can be found in Figure 2A.

It should be noted that, in addition to Schiff base products, another type of adduct can form from α,β-unsaturated carbonyls depending on the site of nucleophilic attack. To clarify, a covalent bond at the carbonyl carbon forms a Schiff base adduct, while covalent bond formation with the β-carbon forms a Michael addition adduct (Figure S1.13). Both reactions are energetically feasible and result in different masses (e.g., *m*/*z* 385 versus 403, Table S1.5).^114^ Other carbonyl derivatization studies in environmental water matrices have ignored the evaluation of both adducts in terms of method performance.^8,9^ In this study, the performances of both the Schiff base and Michael addition analytes were evaluated in terms of recoveries, quantification accuracy, and stability. For clarity in the main text, however, only the Schiff base adducts are represented in all subsequent figures. Further discussion and figures for Michael addition adducts can be found in Supporting Information 1 (S1).

Overall, both nucleophile bead systems provided a significant advantage over the reduced amine bead system, where the peak signals had much greater intensities with clear Gaussian peak shapes (e.g., the signal intensities for butanal in the −ONH_2_ versus reduced −NH_2_ systems were 6.89 × 10^8^ and 3.98 × 10^5^, respectively). In addition to Schiff base products, both −ONH_2_ and −NNH_2_ beads were able to capture the Michael addition products for the α,β-unsaturated carbonyls (see Table S1.5). The only carbonyl in the mixture that could not be detected by any system tested in this work was 2,6-dichloro-1,4-benzoquinone. For the −NNH_2_ bead system, only acrylamide and 1,4-benzoquinone were not able to be detected at any pH (Figure S1.11). For the −ONH_2_ bead system, all carbonyls (except 2,6-dichloro-1,4-benzoquinone) were detected. For the saturated dicarbonyls (glyoxal and methylglyoxal), hydrates were also in equilibrium with carbonyls due to water reacting with the nonderivatized carbonyl to form gem-diols (Figures S2.25 and S2.35). The glyoxal and methylglyoxal hydrates could be detected by both bead systems (*m*/*z*_NNH2_ 419 and *m*/*z*_ONH2_ 405, and *m*/*z*_NNH2_ 433 and *m*/*z*_ONH2_ 419, respectively), with the −ONH_2_ bead system yielding a slightly higher signal (Figure 2A). While hydrate formation of (di)carbonyls is well known,^115−117^ previous OBP studies have ignored the hydrate products during analyses of glyoxal and methylglyoxal, which may have overlooked their presence.^7−9^

At the high concentration (5 μM each carbonyl) used in this study, some dimers also formed in equilibrium with the dicarbonyls (*m*/*z*_ONH2_ 463 for glyoxal, *m*/*z*_NNH2_ 505 for methylglyoxal) as well as acrolein (*m*/*z*_ONH2_ 423) and crotonaldehyde (*m*/*z*_NNH2_ 483). These dimers have been observed previously in atmospheric aerosol studies;^116−118^ however, these dimers exhibited weak signals and are unlikely to form at low, environmentally relevant carbonyl concentrations in disinfected waters, so these adducts were ignored in all subsequent analyses.

Overall, in addition to acrylamide and 1,4-benzoquinone detection, the −ONH_2_ bead system outperformed the −NNH_2_ bead system to a significant extent for saturated carbonyls with long alkyl chains (i.e., butanal and hexanal). While most other peak area signals fell within magnitudes of 10^5^–10^7^, butanal and hexanal both had integrated peak areas of 10^9^. This difference in signal compared to the other carbonyls in the −ONH_2_ system can likely be attributed to aspects such as (i) greater adduct formation/concentrations, (ii) better adduct stability, and/or (iii) better ionization in MS analysis. Due to this difference in behavior for butanal and hexanal compared to the rest of the carbonyls, the butanal-d2 internal standard was used to normalize peak areas for quantification in further analyses for these compounds. Benzaldehyde-d5 was used as the internal standard for the rest of the carbonyls due to its similar signal intensity as well as its use as a proxy internal standard in previous work for detecting carbonyl-TSH adducts.^7,8^ Ideally, an isotope-labeled carbonyl should be used for each unlabeled analyte, but these were not commercially available. For all other carbonyls, the performance between the −NNH_2_ and −ONH_2_ bead systems was similar and did not have a clear pattern in terms of carbonyl class or product type (see Text S1.9 for more details).

Comparing the effect of pH on peak areas for the derivatization reactions, more acidic conditions yielded greater signals for the majority of the carbonyl adducts in both the −NNH_2_ and −ONH_2_ bead systems, with a general trend for peak area exhibiting pH 5 > pH 7 > pH 9 (pH 9 not tested for the −NNH_2_ system; see Figures S1.9–12 for full data sets and Figure 2B for a summary of the pH trends for the −ONH_2_ system). This was expected, as the resulting hydrazone and oxime products are known to be more stable under acidic conditions.^91,113^ For the −ONH_2_ system, only a few exceptions were notable (e.g., methylglyoxal and 1,4-benzoquinone had the highest peak areas at pH 9; Figure 2B). At pH 9, however, the peak areas for the adducts exhibited higher variability compared to the areas obtained from the pH 5 and 7 conditions (*n* = 3 at each condition). In addition, the ratio between the Schiff base products versus Michael addition products for the same carbonyl (e.g., acrolein *m*/*z* 385 vs 403 for the ONH_2_ system, Figure 2B and Figure S1.12) appeared to be greater at pH 5 than pH 7, which is expected due to the aforementioned acid stabilization of the oxime bond (which is not applicable for the Michael addition products). With these results in mind, subsequent experiments were run with the −ONH_2_ bead system at pH 7. First, the −ONH_2_ system compared to the −NNH_2_ system was able to detect a greater number of carbonyls and had slightly higher peak areas for the majority of the Schiff base products at pH 7. Although pH 5 produced a general trend of higher signals for the majority of carbonyl adducts, pH 7 was selected due to its relevance in water treatment facilities as well as to mimic physiological conditions in a toxicity screening for carbonyl binding to biological nucleophiles.

### Recovery Experiments

**Figure 3:**
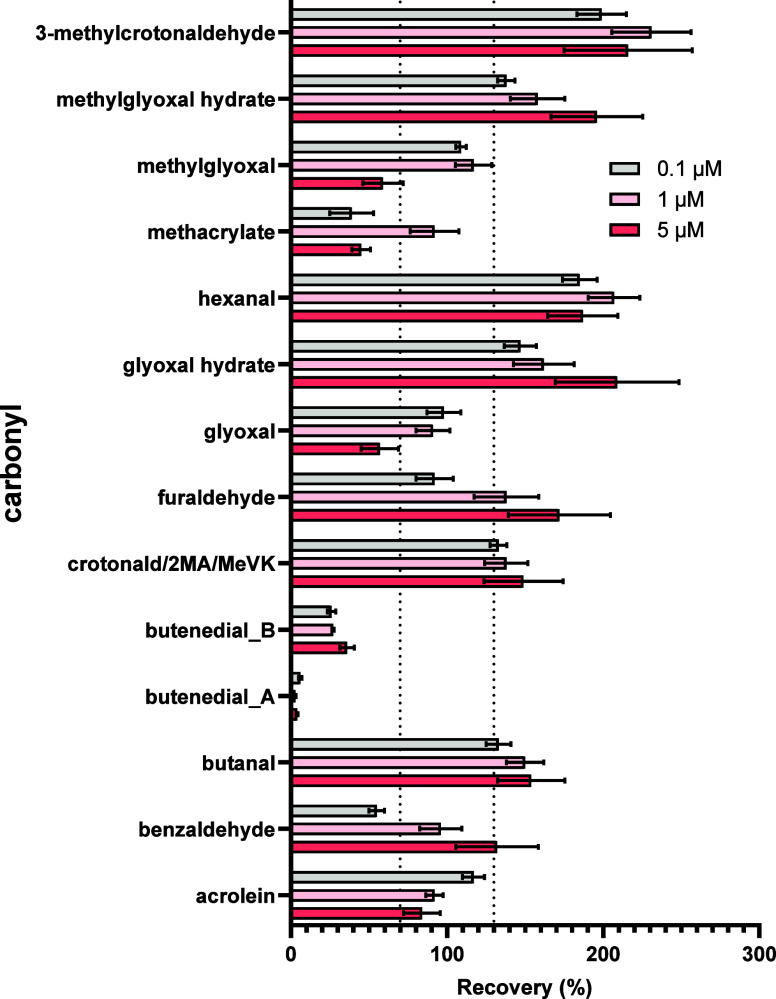
Recoveries (%) of carbonyl adducts after undergoing the SPREx extraction protocol (using 0.1, 1, or 5 μM of each carbonyl). Dashed lines across the *x*-axis indicate the carbonyl adducts that fall within the acceptable range of 70–130% recovery. Values are reported as the averages of each extracted sample peak area divided by the average peak area of the nonextracted samples, with error bars indicating the standard deviation. Michael addition adducts are presented in Figure S1.14. Butenedial is depicted twice, as two chromatographically resolved peaks were detected (Figures S2.3–4). Hydrate formation from glyoxal and methylglyoxal was also observed and included in these results. Acrylamide and 1,4-benzoquinone were not included due to poor signals and reproducibility.

One of the key advantages offered by the SPREx assay is the convenience of its built-in reactivity-directed extraction feature to avoid time-consuming extraction procedures such as SPE, which notably perform poorly for analyses of polar and/or volatile OBPs such as carbonyls.^87−90^ The recoveries from the SPREx extraction procedure are reported in Figure 3. Overall, recoveries were >100% for many of the carbonyls. It is important to note that errors can be introduced in the accuracy of the recovery results due to the difference between the extracted and nonextracted samples. To clarify, adducts were formed in the extracted samples utilizing only the nucleophile probe (because the nucleophile was still linked to the bead), whereas for the nonextracted samples, nucleophiles were first cleaved from the bead, allowing adduct formation to occur on either the cleaved −NH_2_ end group or the target −ONH_2_ nucleophile probe (refer to structures in Figure S1.1). While amines were not observed to form stable adducts with the majority of the carbonyls (see previous discussion on observed stability issues using amine probes), it is possible that the exposed amine end group could compete with the −ONH_2_ group during the reaction step, decreasing the concentration of the stable ONH_2_-carbonyl adduct. Furthermore, 2-butenedial likely demonstrated poor recovery (<∼5 or 40%; Figure 3) relative to the rest of the carbonyls since 2-butenedial was the only analyte observed to form stable Schiff base adducts with the −NH_2_ beads. Methylglyoxal, methacrylate, glyoxal, furaldehyde, and acrolein fell within the acceptable 70–130% recovery range via SPREx at the lowest concentration tested (0.1 μM).

Overall, SPREx represents an effective new approach to extract electrophilic analytes that are lost when other approaches such as SPE are used. The recovery values obtained via SPREx are a significant improvement compared to those obtained via SPE. For instance, the spiking recovery of acrolein post-SPE extraction was determined to be <1% in one study,^83^ but the recovery for acrolein via SPREx was 117% for the acrolein-aminooxy bead adduct at the spiked 0.1 μM concentration (Figure 3).

### Accuracy Experiments

The obtained results highlight that the SPREx approach provides key advantages for the simultaneous extraction, detection, and identification of toxic carbonyl analytes in a qualitative manner. While qualitative analyses are impactful to test for the formation of OBPs, further analyses were performed to test the capacity of SPREx as a quantitative assay.

**Figure 4:**
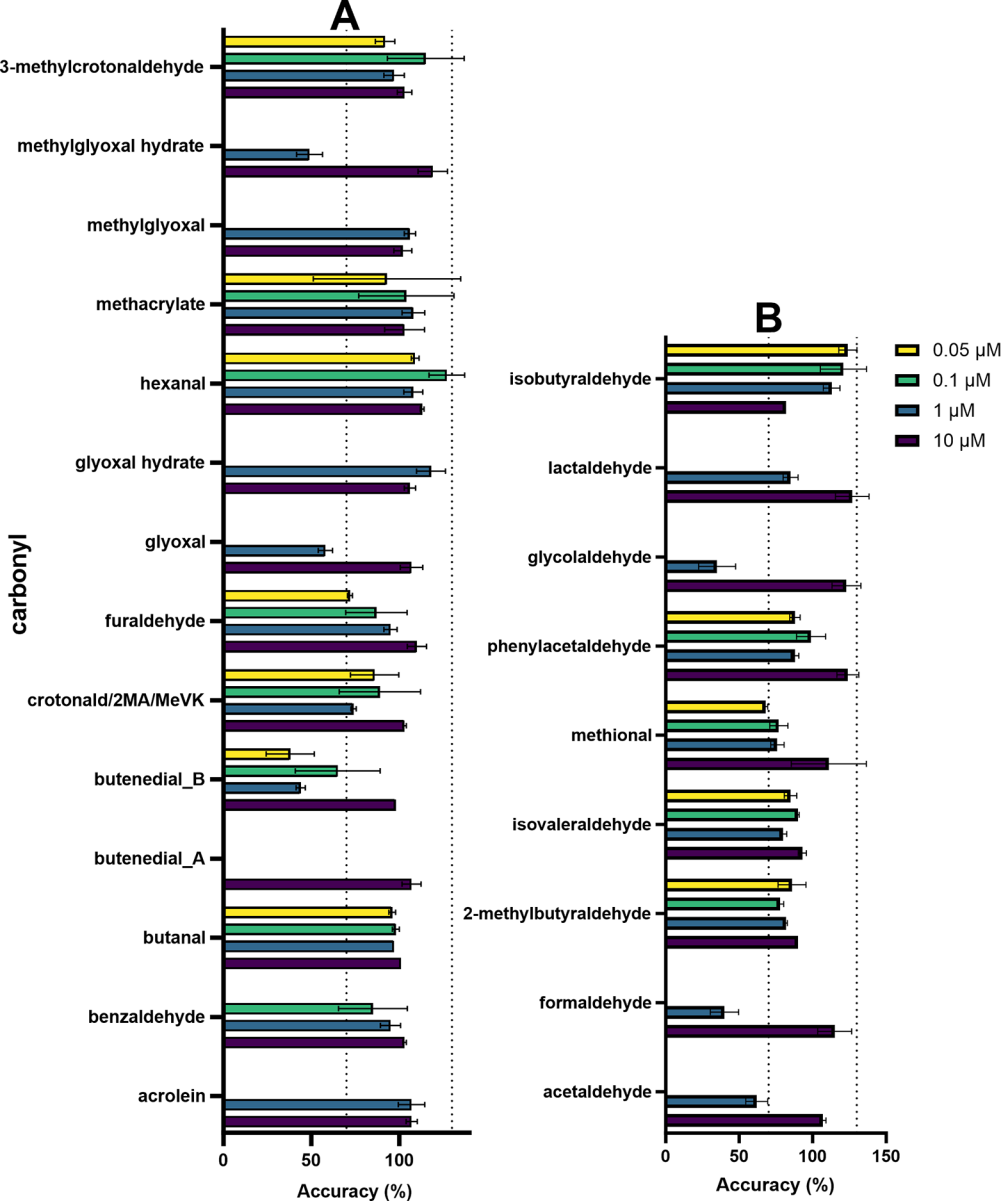
Accuracy (%) of quantification at different spiked control concentrations of (A, left) the carbonyls tested in the nucleophile probe and recovery experiments and (B, right) the carbonyls generated from amino acid chlorination. Dashed lines indicate the signals that fell within the acceptable range of 70–130% accuracy. Missing values indicate that the concentration was below the LOQ. Values are reported as the ratio (%), indicating the averages of the triplicate samples over the known spiked concentration with error bars indicating the standard deviation. Butenedial is depicted twice, as two chromatographically resolved peaks were detected (see Figures S2.3–4).

To ensure that accurate quantification of the carbonyl adducts could be accomplished in the chlorination of AAs and environmental matrix applications of SPREx (see below), the quantification accuracy of spiked controls containing known carbonyl concentrations was determined. The results of these experiments are depicted in Figure 4A using the same carbonyl mixture used during the nucleophile probe testing and recovery experiments for the Schiff base adducts discussed above. The results for the Michael addition adducts for applicable carbonyls are depicted in Figure S1.15.

In addition to these carbonyls, a separate set of spiked control experiments was carried out for carbonyls produced via AA chlorination that are relevant in the following applications section (Figure 4B).

All carbonyls at 10 μM were accurately quantified within the 70–130% range (Figure 4 and Figure S1.15). As the concentration decreased, however, the quantification of many of the polar carbonyls was less accurate. This is likely due to the higher background concentrations observed from the polar carbonyls impacting the limits of detection and quantification (Text S1.4). However, even at more environmentally relevant concentrations (e.g., 0.05 μM), many of the carbonyls were still accurately quantified within the 70–130% range (Figure 4 and Figure S1.15). These carbonyls included 3-methylcrotonaldehyde via Michael addition and butanal, crotonaldehyde/2-methacrolein/methyl vinyl ketone (isomers), furaldehyde, hexanal, 3-methylcrotonaldehyde, 2-methylbutyraldehyde, isovaleraldehyde, phenylacetaldehyde, and isobutyraldehyde via Schiff base formation. Additionally, benzaldehyde, 2-butenedial, methacrylate, and methional achieved high accuracy in the 0.1 μM range. Only glyoxal, methylglyoxal hydrate, acetaldehyde, formaldehyde, and glycolaldehyde fell out of the 70–130% accuracy range at 1 μM.

The lack of available isotope-labeled internal standards for each carbonyl may also be a key factor impacting the measured accuracies (Figure 4 and Figure S1.15). Since the peak area of each derivatized carbonyl was normalized to the benzaldehyde-d5 peak area (excluding butanal and hexanal), differences in carbonyl behavior during SPREx derivatization/extraction and LC-HRMS analysis would contribute to higher error if this behavior deviated from the internal standard. For instance, normalizing the adduct signal of butanal to the signal of butanal-d2 achieved accuracies of 96, 98, 97, and 101% for the 0.05, 0.1, 1, and 10 μM spiked controls, respectively. In contrast, normalizing to benzaldehyde-d5 yielded accuracies of 47, 55, 58, and 103%, respectively. However, we were constrained to using the only two commercially available standards, limiting the design of the accuracy experiments, but other quantitative carbonyl studies have also used the benzaldehyde-d5 internal standard as a best approximation.^7,8^

### Stability

To ensure accurate measurements of carbonyl concentrations were consistent over the duration of LC-HRMS analysis, stability experiments were conducted by evaluating spiked control accuracies at the beginning, middle, and end of an ∼72 h analysis. The results of these experiments are illustrated in Figures S1.16–18.

If degradation or instability of the Schiff base adducts were occurring, there would be a clear trend of decreasing accuracy from the beginning to the end of the LC-HRMS analysis (Figure S1.16). For the SPREx carbonyl adducts, no clear degradation trend was observed for the majority of the carbonyls. Only benzaldehyde (0.05 and 0.1 μM) and acrolein (1 μM) showed a notable drop in accuracy from beginning to the end of the LC-HRMS analysis, which may be due to instability at lower concentrations, as this trend was not observed for the higher spiked control concentrations. The observed stability for the carbonyl adducts is an improvement over previous methods, where one study using *p*-toluenesulfonyl hydrazide (TSH)-carbonyl derivatization in environmental water analyses observed significant losses for a few carbonyls, including complete loss of glyoxal, 40% loss of formaldehyde, 39% loss of crotonaldehyde, and 32% loss of methacrolein after 1 day.^8^ Our results indicate that these losses can be substantially reduced when using SPREx.

### Application of SPREx

The advantages and limitations of the SPREx method were evaluated by focusing on carbonyls as an important class of OBPs, as they provide unique challenges in terms of poor ionizability in LC-HRMS and poor extraction recoveries during SPE due to their polar and volatile nature. More importantly, carbonyls possess high toxicities due to their electrophilicity^15^ and are known byproducts of water disinfection via chlorination and ozonation.^7,10,101^ Many carbonyls have been overlooked in previous OBP studies due to the biases of the extraction procedures or the lack of derivatization reagents. However, they have been receiving recent attention in the literature.^7−10,82,84,86^

In order to evaluate the applicability of the developed approach for the detection of toxic electrophiles such as carbonyls, we utilized SPREx for the detection of carbonyls formed in laboratory chlorination experiments and different environmental matrices (wastewater and drinking water). All quantified carbonyls were validated by matching the retention times to reference standards during LC-HRMS analysis, and these chromatographs and fragmentation data are provided within Figures S2.2–37.

#### Formation of Carbonyls during Chlorination of Free Amino Acids

It is well known that nonpolar AAs (both free and within peptides) degrade to odorous aldehydes during chlorination (Figure S1.19),^101−103,106,107,119^ which impacts water quality in terms of aesthetics for consumers as well as toxicity. Polar AA systems have received less attention, however, likely due to the detection challenges associated with higher polarity and lower molecular weight carbonyls (see previous discussion). Additionally, these AAs are relevant precursors in many environmental water matrices, especially in wastewater and algae-infested waters.^110,112^ Therefore, after the SPREx assay method was established, its performance was assessed in chlorination applications targeting carbonyl formation from nine different AA systems ranging in polarity. Results of the recoveries of the different AA carbonyls, quantification accuracy, and adduct stability are depicted in Figure 4B and Figures S1.18 and 20. Briefly, the recoveries for all nine AAs for all three concentrations tested (0.1, 1, and 5 μM) fell within or were only slightly above the acceptable 70–130% recovery range (Figure S1.20). The stability results indicated no sign of adduct degradation over the course of the LC-HRMS analysis, except for formaldehyde, acetaldehyde, and glycolaldehyde at the lower concentrations (approximately 72 h; Figure S1.18).

**Figure 5:**
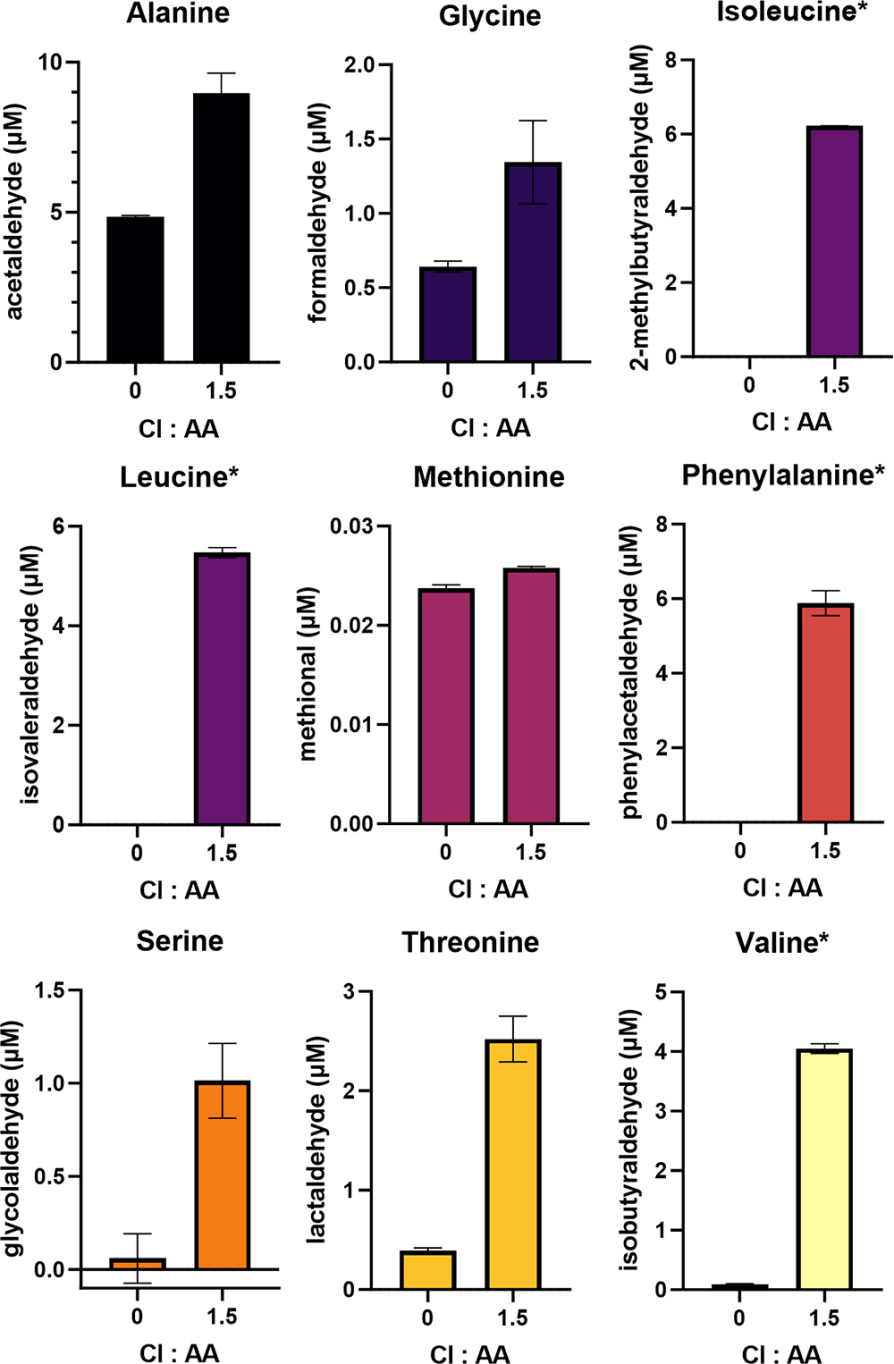
Aldehyde concentration versus chlorine dose for each amino acid system (*n* = 3). Initial concentrations of the amino acids were 10 μM. Nonpolar amino acid systems are indicated with an asterisk (*). Error bars indicate the standard deviations.

The results of the carbonyl concentrations from each of the nine chlorinated AA systems are depicted in Figure 5.

For all AAs, there was a statistically significant increase in carbonyl concentrations when chlorine was added (0 versus 1.5 Cl:AA). The nonpolar AA systems (* in Figure 5) have been studied extensively in previous literature, and one study by Froese et al. determined that at reaction conditions similar to this work (i.e., pH 7, 1.5 Cl:AA, and a 2 h reaction time), the carbonyl yields for isobutyraldehyde, 2-methylbutyraldehyde, isovaleraldehyde, and phenylacetaldehyde were approximately 56, 61, 78, and 86%, respectively (% of theoretical).^103^ Quantifying the nonpolar aldehyde yields via SPREx, it was determined that these results were slightly lower for three of the four aldehydes at 41, 62, 55, and 59%, respectively (% of theoretical).

The nonpolar aldehydes also did not exhibit the significantly higher background signals of aldehydes in the 0 Cl:AA dose, as seen in the polar AA systems. While aldehydes such as acetaldehyde and formaldehyde are known to be abundant in laboratory air and reagent water,^9^ it is unlikely this is the source of aldehydes in the 0 Cl:AA controls since these results were corrected for background contamination. One possibility for these observed signals could be due to decomposition of the AAs themselves without the presence of chlorine, which has been noted before in previous studies, although in different matrices (e.g., the food chemistry field).^120^ However, further research is necessary to confirm this speculation and determine the contributing factors. Nevertheless, the SPREx assay was able to show a statistically significant increase in aldehyde concentrations between the nonchlorinated and chlorinated polar AA systems (*t* test: α = 0.05, *p*-values <0.05). For alanine, glycine, methionine, serine, and threonine, their respective aldehyde concentrations were 8.97, 1.34, 0.03, 1.01, and 2.52 μM, respectively. After background subtraction, the total contributions of aldehyde formation from AA chlorination were 4.12, 0.70, 0.002, 0.95, and 2.13 μM, respectively. For methionine, the detected concentrations for its corresponding carbonyl methional were notably lower than the carbonyl yields for the other AA systems. As methionine was the only sulfur-containing AA analyzed in this study, its difference in reactivity is likely due to the oxidation of the sulfur atom by hypochlorite.^121−123^ This reaction produces methionine sulfoxide, which, following the mechanism in Figure S1.19, would further react to produce the sulfoxide analogue of methional, i.e., 3-methylsulfinylpropanal (CAS: 393781-95-8). Indeed, a strong signal was detected for the aminooxy-3-methylsulfinylpropanal adduct that was ∼118× higher in the chlorinated versus nonchlorinated methionine system (Figure S1.21). The MS^2^ data further supports the sulfoxide analogue, as the fragments *m*/*z* 340 and 385 indicate the loss of a sulfoxide moiety (Figure S2.34). However, no standard was commercially available to quantify this product. Nevertheless, this evidence indicating a different major product resulting from methionine chlorination supports the low methional yields that were observed.

#### Quantification of Carbonyls in Drinking Water and Wastewater

**Figure 6:**
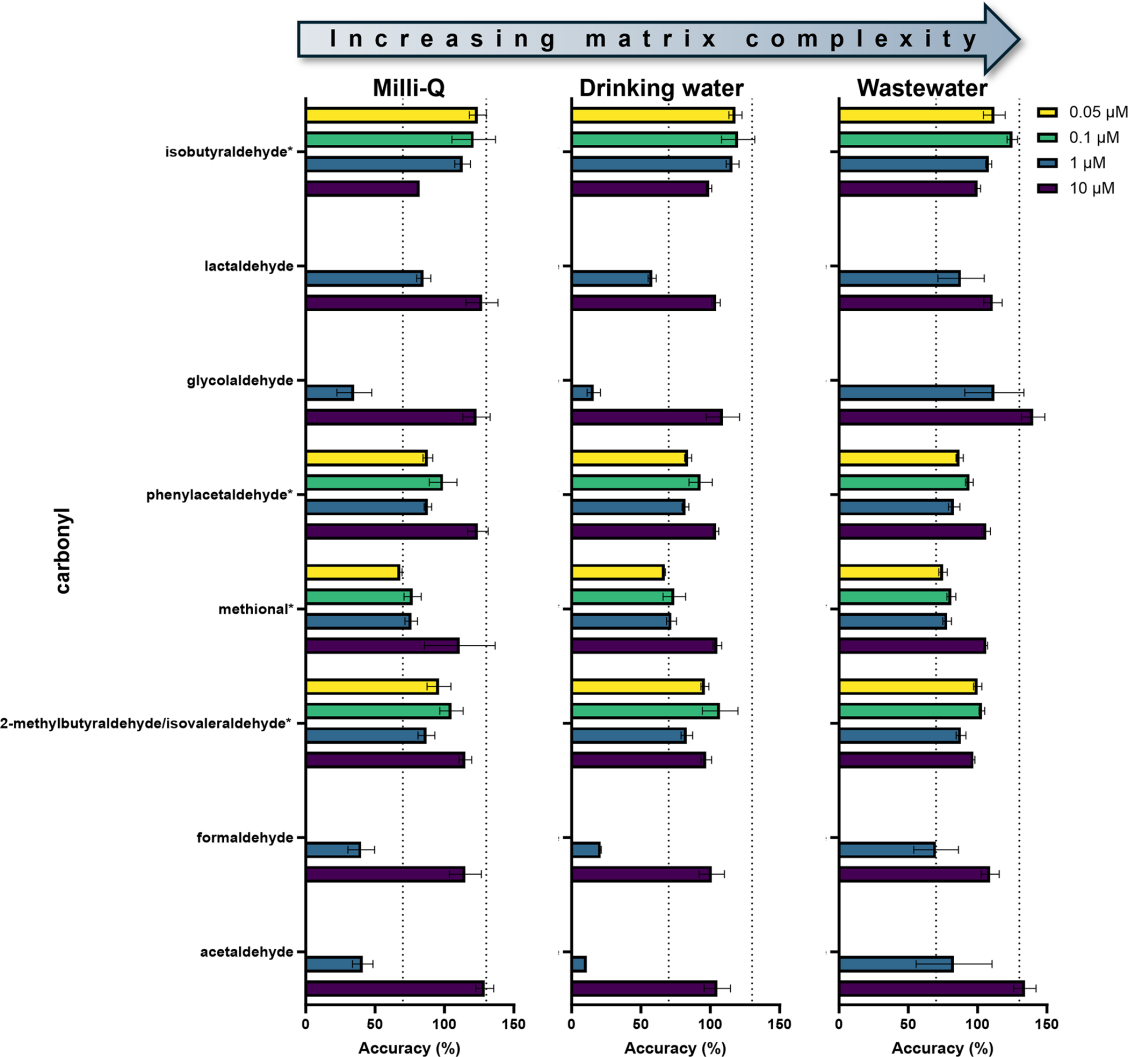
Quantification accuracies of carbonyls (0.05, 0.1, 1, and 10 μM) spiked into Milli-Q water (left), drinking water (middle), and wastewater (right). Dashed lines indicate the acceptable range of accuracy from 70 to 130%. All values are the averages of triplicates with error bars showing the standard deviation. The more nonpolar carbonyls are indicated by an asterisk (*) on the *y*-axis.

In addition to evaluation of the SPREx assay for the detection of carbonyls formed during the chlorination of AAs, its performance was evaluated for the quantification of carbonyls in drinking water and wastewater. As displayed in Figure 6, quantification accuracy did not significantly change for most carbonyls when the complexity of the environmental water matrix increases from Milli-Q water to drinking water and wastewater.

As seen previously, all carbonyls exhibited an acceptable quantification accuracy (70–130%) at the highest spiked concentration (10 μM). For the carbonyls with higher polarities, quantification accuracies decreased with lower spiked concentrations, which is likely attributable to higher background contamination of the more polar, lower molecular weight carbonyls. At these lower concentrations, many of the polar carbonyls were below their limits of quantification (Text S1.4). Quantification accuracy for some of these more polar carbonyls may also be limited by their tendency to oligomerize and/or form hydrates,^115−118^ thus decreasing the availability of the free, monomer form of the carbonyl to be derivatized, especially at low concentrations. Nevertheless, for the more nonpolar carbonyls (* in Figure 6), quantification accuracy at the lowest spiked concentration (0.05 μM) was achievable via SPREx. Overall, these results suggest that, for environmentally relevant concentrations (nM–low μM range), SPREx demonstrates an added feature for use as a quantitative assay for many carbonyls in environmental water matrices depending on the variability in matrix composition. For instance, from chlorinated surface and pool waters to ozonated drinking water, wastewater, and surface waters, carbonyls such as acetaldehyde, glyoxal, butyraldehyde, and isovaleraldehyde have been detected at concentrations in the ∼0.1–42 μM,^7,100,124,125^ 0.25–38 μM,^100,125^ 0.05–0.91 μM,^100,124,125^ and 0.06–0.14 μM^106^ ranges, respectively. As such, SPREx represents a suitable approach for the quantification of these carbonyls in relevant aqueous matrices. It should be noted that the quantification accuracy could be further improved by reducing carbonyl backgrounds in the controls and obtaining internal standards for each carbonyl. Nevertheless, the spiked control experiments demonstrate the importance of determining quantification accuracies in carbonyl studies, as consistent accuracy values can help correct for under- or overestimated carbonyl concentrations.

As mentioned, in addition to its performance as a quantitative assay, SPREx serves an additional purpose as a qualitative screening tool to extract, detect, and identify carbonyls in an environmental water sample. For future work in the applications of SPREx, we can assess the effects of the presence of OBPs in environmental mixtures including the identification of unknown OBPs. Screening for the unknown OBPs will be simplified by the discovery of signature fragments (*m*/*z* 229 and 257; see Figure S2.1) and common neutral loses from the SPREx nucleophile–electrophile adducts in developing a nontargeted LC-HRMS method.

## Conclusions

A new method, SPREx, was successfully developed to achieve simultaneous extraction, identification, and prioritization of (toxic) electrophilic OBPs. The synthesized aminooxy beads and hydrazide beads enabled higher stability of carbonyl adducts compared to thiol and amine beads (with and without reducing agents) and were capable of targeting carbonyls of different product types (i.e., Schiff base versus Michael addition) and functional groups. Additionally, the SPREx assay demonstrated markedly improved recovery rates with few extraction steps. Notably, the majority of carbonyl adducts showed no significant signs of instability over the course of LC-HRMS analysis. To test the quantitative limits of SPREx, application experiments demonstrated that (1) accurate quantification was achieved at environmentally relevant concentrations for nonpolar carbonyls but decreased with more polar carbonyls (e.g., the current ONH_2_ SPREx system might be better suited for qualitative analyses of polar carbonyls depending on the matrix) and (2) carbonyl compound concentrations could be quantified in increasingly complex environmental matrices, ranging from ultrapure water to wastewater/drinking water. While these advantages are significant, it is also important to note limitations observed with SPREx. For derivatization, the aminooxy (−ONH_2_) nucleophile probe exhibited greater stability for amine-carbonyl adducts, but as a toxicity prioritization probe, it may not be as biologically relevant compared to amines and thiols. However, this is a necessary trade-off between detection/identification and prioritizing toxic analyte derivatization when developing nucleophile probes.

Based on these notable benefits and limitations, we conclude that SPREx can be utilized as a preferred extraction method since electrophilic OBPs are often lost during SPE. As a quantitative assay, SPREx yielded acceptable results, especially for nonpolar carbonyls, if background controls were utilized. For unknown OBPs, SPREx could serve as a useful screening tool for treated environmental water matrices to extract, detect, and identify toxic electrophiles. Now that the SPREx method has been established using carbonyls as model OBPs with the aminooxy derivatization probe, future work will concentrate on expanding the SPREx approach to optimize the derivatization and identification of other organic electrophiles in a nontargeted analysis of treated water matrices. The demonstration of successful, one-step click chemistry synthesis of multiple different nucleophile labels—featuring both hard (amine) and soft (thiol) reactivities—establishes the basis for future work regarding more widespread detection of both hard/soft electrophilic OBPs such as halogenated (soft) OBPs in addition to carbonyls (hard/soft).

## Supporting Information Available

Standards and reagents, nucleophile bead synthesis and characterization, supporting figures, calibration curves, limits of detection, LC-HRMS settings, full list of nucleophile-carbonyl *m*/*z* values, figures for −NNH_2_ adducts, figures for all Michael addition adducts, stability experiment figures, and amino acid-related carbonyl recoveries (PDF)
All selected ion chromatograms and MS^2^ spectra for nucleophile-carbonyl adducts (PDF)
