# Supplementary material for: Solid-Phase Reactivity-Directed Extraction (SPREx): An Alternative Approach for Simultaneous Extraction, Identification, and Prioritization of Toxic Electrophiles Produced in Water Treatment Applications
Source: ACS Environ Au. 2024 Sep 20;4(6):317–32. doi: 10.1021/acsenvironau.4c00025 (PMC11583095; doi:10.1021/acsenvironau.4c00025)
Supplement: Supplementary file 1 — vg4c00025_si_001.pdf [file vg4c00025_si_001.pdf]

Supporting Information 1

Solid-phase reactivity-directed extraction (SPREx): an alternative approach for simultaneous extraction, identification, and prioritization of toxic electrophiles produced in water treatment applications

Daisy N. Grace,[1] Matthew N. Newmeyer,[1] Carsten Prasse[1,2,]*

[1]Department of Environmental Health and Engineering, Johns Hopkins University, Baltimore, MD 21218, USA.

[2]Risk Sciences and Policy Institute, Bloomberg School of Public Health, Johns Hopkins University, Baltimore, MD 21205, USA.

*Corresponding author. Email: cprasse1@jhu.edu

Content

Text

Text S1.1. Standards and reagents.	S4
Text S1.2. Cleavable microbead synthesis via CuAAC click chemistry.	S6
Text S1.3. Characterization of nucleophile concentrations on the synthesized microbeads.	S8
Text S1.4. Determination of linear ranges, limits of detection (LOD), limits of quantification (LOQ), and discussion of background carbonyl concentrations.	S16
Text S1.5. LC-HRMS analysis.	S19
Text S1.6. Nucleophile probe testing: comparing the cleavable linkers.	S19
Text S1.7. Nucleophile probe testing: thiol probe results & discussion.	S20
Text S1.8. Nucleophile probe testing: NH_2 and reduced NH_2 systems.	S20
Text S1.9. Nucleophile probe testing: further discussion on the ONH_2 vs. NNH_2 beads systems.	S27

Tables

Table S1.1. Structures, names, CAS numbers, purities, and manufacturers of all carbonyls analyzed in this work. S5

Table S1.2. LODs for the Schiff base carbonyl adducts in the PC-ONH_2 bead system. S16

Table S1.3. LODs for the Michael addition carbonyl adducts in the PC-ONH_2 bead system. S17

Table S1.4. Structures of all amino acids tested in this study, along with the expected aldehyde formation based on known transformation mechanisms. S18

Table S1.5. Expected carbonyl adducts in the SPREx assay method development. S21

Figures

Figure S1.1. Schematic of the CuAAC click chemistry reaction for bead synthesis and the final cleaved adducts. S7

Figure S1.2. Schematic of the Cu^+/BCA thiol quantification assay. S9

Figure S1.3. External calibration curves of the Cu^+/BCA assay. S10

Figure S1.4. Structures of all synthesized nucleophile-labeled bead systems. S10

Figure S1.5. Experimental procedures used to analyze recovery of the carbonyl adducts. S11

Figure S1.6. Experimental procedures used to analyze the accuracy of the carbonyl quantification. S11

Figure S1.7. Calibration curves for carbonyl quantitative analysis. S12

Figure S1.8. Schiff base adduct formation from carbonyl + amine reactions and instability in water. S21

Figure S1.9. ONH_2 versus NNH_2 nucleophile-carbonyl adduct peak areas at pH 5. S23

Figure S1.10. Peak areas (log_{10} scale) for all Michael addition carbonyl adducts applicable for α,β-unsaturated carbonyls only. S24

Figure S1.11. pH impact on carbonyl adduct peak areas (log_{10} scale) in the NNH_2 bead system for all carbonyls. S25

Figure S1.12. pH impact on carbonyl adduct peak areas (log_{10} scale) in the ONH_2 bead system for the Michael addition adducts for the α,β-unsaturated carbonyls. S26

Figure S1.13. Michael addition versus Schiff base reaction pathways. S26

Figure S1.14. Recoveries of amino acid carbonyl adducts after undergoing the SPREx extraction protocol. S27

Figure S1.15. Accuracy of quantification of the Michael addition carbonyls adducts. S28

Figure S1.16. Stability experiments of triplicate spiked controls of the Schiff base carbonyl adducts injected during the beginning, middle, and end of a 72-hour LC-HRMS analysis. S29

Figure S1.17. Stability experiments of triplicate spiked controls of the Michael addition carbonyl adducts injected during the beginning, middle, and end of a 72-hour LC-HRMS analysis. S30

Figure S1.18. Stability experiments of triplicate spiked controls of the amino acid-related carbonyls. S31

Figure S1.19. Abbreviated mechanism of amino acid transformation to aldehydes during chlorination. S31

Figure S1.20. Recoveries of amino acid carbonyl adducts after undergoing the SPREx extraction protocol. S32

Figure S1.21. Peak area for 3-methylsulfinylpropanal (normalized to the benzaldehyde-d5 internal standard) versus chlorine dose for the methionine amino acid system. S33

Text S1.1: Standards and Reagents

Solvents and buffers. All phosphate or carbonate/bicarbonate salts for buffer preparations and organic solvents for relevant stock solution preparations were ACS grade quality from Fisher Scientific. All aqueous solutions and mobile phases utilized ultrapure water (Milli-Q; 18 MΩ cm resistivity equipped with a VOC-Pak from Millipore Sigma). All organic solvents or additives used for liquid chromatography analysis were Optima LC/MS grade from Fisher Scientific.

Microbead synthesis. PC alkyne agarose and Dde alkyne agarose were obtained from Click Chemistry Tools (now acquired by Vector Laboratories; SKU: CCT-1142). As per manufacturer specifications, these beads (size: 50-150 µm) consist of 6% crosslinked agarose resin functionalized with alkyne groups (5-20 µmol alkyne/mL resin) and a photocleavable linker or Dde cleavable linker, respectively. Nucleophile-azide labels include azido-PEG3-amine (CAS: 134179-38-7; 98%), azido-PEG4-hydrazide (CAS: 2170240-96-5; 95%), thiol-PEG3-azide (CAS: 1347705-79-1; > 95%), and aminooxy-amido-PEG3-azide (CAS: n/a; >95%) and were obtained from Conju-Probe. Copper (II) sulfate (CAS: 7758-98-7; ≥99%), tris-hydroxypropyltriazolylmethylamine (THPTA; CAS: 760952-88-3; >95%), sodium ascorbate (CAS: 134-03-2; ≥98%), aminoguanidine hydrochloride (CAS: 1937-19-5; >98%), tris(2-carboxyethyl)phosphine hydrochloride (TCEP; CAS: 51805-45-9; ≤100%), and hydrazine monohydrate (CAS: 7803-57-8; 99%) were obtained from Sigma Aldrich, Lumiprobe, TCI America, VWR, Sigma-Aldrich, and Alfa Aesar, respectively.

Microbead nucleophile quantification. Bicinchoninic acid disodium salt (CAS: 979-88-4; >98%) was obtained from VWR, 2-iminothiolane hydrochloride (Traut's reagent; CAS: 4781-83-3; 98%) was purchased from Acros Organics, N-acetyl-l-cysteine (CAS: 616-91-1; >98%) was obtained from Alfa Aesar, and both copper (II) sulfate pentahydrate (CAS: 7758-99-8; Lab grade) and sodium L-(+)-tartrate dihydrate (CAS: 6106-24-7; ACS grade (99.0-101.0%)) were obtained from Fisher.

Nucleophile probe testing. Sodium cyanoborohydride (CAS: 25895-60-7; ≥98%) and sodium triacetoxyborohydride (CAS: 56553-60-7; 95%) were obtained from Chem Impex and Beantown Chemical, respectively. Information for all carbonyls tested via SPREx is presented in Table S1.1.

Amino acid chlorination. Sodium hypochlorite solution (CAS: 7681-52-9; 5% available chlorine) was obtained from J.T. Baker. L-isoleucine (CAS: 73-32-5; ≥99.5%), L-leucine (CAS: 61-90-5; ≥98%), L-methionine (CAS: 63-68-3; ≥98%), L-phenylalanine (CAS: 63-91-2; ≥99.0%), L-serine (CAS: 56-45-1; 98.5-101%), and L-valine (CAS: 72-18-4; ≥99.5%) were obtained from Sigma Aldrich, while L-alanine (CAS: 56-41-7; 99.80%) and L-glycine (CAS: 56-40-6; 99.50%) were obtained from Chem Impex International, Inc. L-threonine (CAS: 72-19-5; 98%) was purchased from Thermo Scientific.

Table S1.1. Structures, names, CAS numbers, purities, and manufacturers of all carbonyls analyzed in this work.

	Carbonyl	CAS	Purity	Manufacturer
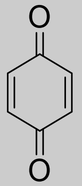	1,4-benzoquinone	106-51-4	Certified reference	Sigma Aldrich
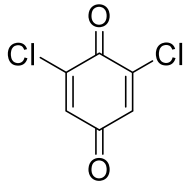	2,6-dichloro-1,4-benzoquinone	697-91-6	>97%	Alfa Aesar
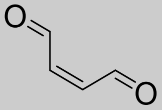	2-butene-1,4-dial	2363-83-9	n/a	synthesized[1]
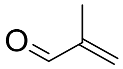	2-methacrolein	78-85-3	95%	Sigma Aldrich
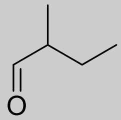	2-methylbutyraldehyde	96-17-3	98%	Combi-blocks
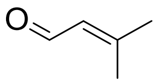	3-methylcrotonaldehyde	107-86-8	97%	Sigma Aldrich
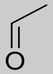	acetaldehyde	75-07-0	40% w/w aq. soln	Neta Scientific
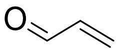	acrolein	107-02-8	5000 ug/ml in H_2O	Restek
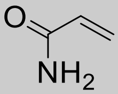	acrylamide	79-06-1	>99%	Sigma Aldrich
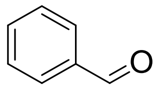	benzaldehyde	100-52-7	≥99%	Sigma Aldrich
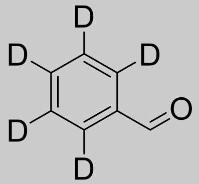	*benzaldehyde-2,3,4,5,6-d_5	14132-51-5	>96%; 99% atom D	CDN Isotopes
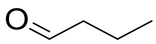	butanal	123-72-8	≥99.5%	Sigma Aldrich
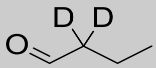	*n-butyraldehyde-2,2-d_2	n/a	96%; 98% atom D	CDN Isotopes
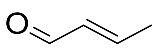	crotonaldehyde	4170-30-3	1:20 cis:trans isomers; ≥99.5%	Sigma Aldrich

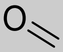	formaldehyde	50-00-0	36.0-38.0% in water	Sigma Aldrich
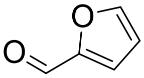	furaldehyde	98-01-1	99%	Sigma Aldrich
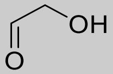	2,5-dihydroxy-1,4-dioxane (glycolaldehyde dimer)	23147-58-2	>98%	Santa Cruz Biotechnology, Inc.
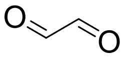	glyoxal	107-22-2	40% w/w aq. soln	Alfa Aesar
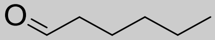	hexanal	66-25-1	98%	Sigma Aldrich
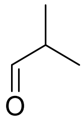	isobutyraldehyde	78-84-2	≥98.0%	TCI America
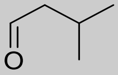	isovaleraldehyde	590-86-3	99%	Oakwood Chemical
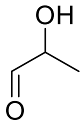	lactaldehyde	598-35-6	1 M in H_2O	Sigma Aldrich
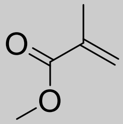	methacrylate	96-33-3	99%	Sigma Aldrich
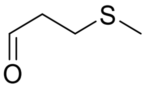	methional	3268-49-3	97%	Thermo Scientific
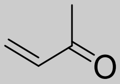	methyl vinyl ketone	78-94-4	90%; contains 0.3-1.0% hydroquinone as stabilizer	Sigma Aldrich
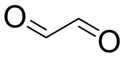	methylglyoxal	78-98-8	40% in H_2O	Sigma Aldrich
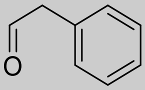	phenylacetaldehyde	122-78-1	≤100%	Sigma Aldrich

<u>Text S1.2. Cleavable microbead synthesis via CuAAC click chemistry</u>

To synthesize the cleavable nucleophile beads, 240 µL of PC (or Dde) alkyne agarose beads were reacted with 10 mM of azide-labeled nucleophiles in a final volume of 1.5 mL in 50 mM phosphate buffer (pH 7.6). The nucleophiles used in this study were amine (NH_2), thiol (SH), hydrazide (NNH_2), and aminooxy (ONH_2) groups; see Text S1.1). Copper (II) sulfate (2.4 mM) was used as the copper source in the CuAAC reaction and kept in the catalytically active Cu (I) state by adding sodium ascorbate (12 mM) as the reducing agent. Note that tris(2-carboxyethyl)phosphine hydrochloride (TCEP; 24 mM) was also tested as a reducing agent for the thiol bead system to additionally aid in reducing potential disulfide bonds, but it was found that TCEP completely

inhibited the CuAAC reaction, likely due to binding to the copper centers or reducing the azides.[2,3] Tris-hydroxypropyltriazolylmethylamine and aminoguanidine were added in a 1.6 and 0.42 ratio relative to the copper concentration, respectively, to accelerate the CuAAC reaction or reduce reactions between ascorbate oxidation products and nucleophile probes, respectively.[2,4–7] The CuAAC reaction was allowed to proceed overnight with shaking. Subsequently, the bead solution was centrifuged at 8000xg for five minutes, and the supernatant was removed and reconstituted with 1.5 mL of Milli-Q water. This step was repeated five times to wash the beads, and the stock was refilled with 1.5 mL of Milli-Q water to create the final stock of nucleophile-labeled, cleavable microbeads.

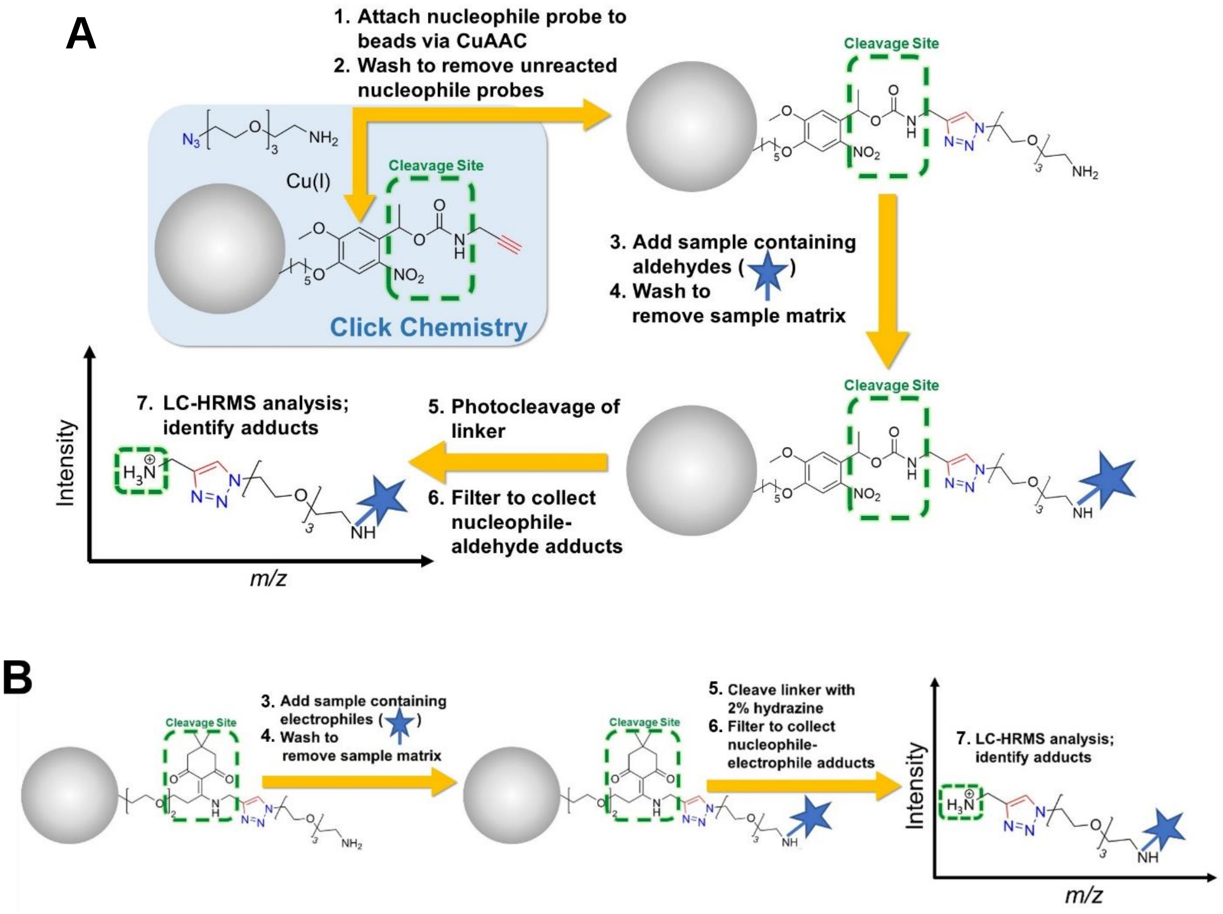

Figure S1.1. A) Schematic of the CuAAC click chemistry reaction between the photocleavable-alkyne agarose beads (alkyne group in red) and the amine (NH_2)-azide nucleophile label (azide = N_3 group in blue) (steps 1 & 2), the addition of carbonyl analytes (steps 3&4), and subsequent photocleavage (steps 5-7; cleavable linker circled by dashed green lines). **B)** Analogous Dde-cleavable bead system post-synthesis and the subsequent cleaved structure enabled by the addition of 2% hydrazine.

Text S1.3. Characterization of nucleophile concentrations on the synthesized microbeads

Multiple methods were tested to discern the nucleophile functionalization on the beads. ^{1}H-NMR was not applicable due to the large size of the beads causing settling and colloidal instability in the bead stock solution. LC-HRMS was also not feasible for bead quantification: first, the solid-phase beads cannot be directly injected into the instrument, and secondly, no standard is available for the cleaved nucleophile to enable quantification with an external calibration curve. Therefore, quantitative UV-visible spectroscopic assays were utilized to calculate the estimated concentration of nucleophiles. These assays have been used previously for the quantification of nucleophilic thiols and amines on proteins,[8,9] and a Cu^{+}/bicinchoninic acid (BCA) assay was determined to be the most effective for amine/thiol quantification in the bead systems. Other UV-vis quantitative assays were conducted but deemed to be incompatible with the bead systems due to producing very low nucleophile yields, high limits of detection, and high error due to adduct instability, especially in aqueous solutions (results not shown). These assays include derivatizing the amines or thiols with 4-nitrobenzaldehyde or o-phthalaldehyde and measuring the absorbance or fluorescence, respectively, of the resulting product.[8–11] The Cu^{+}/BCA assay does not rely on derivatization, but rather the reduction of Cu^{2+} to Cu^{+} by the target nucleophilic thiol and formation of a chromophore with two equivalents of BCA (Figure S1.2, Step 2).[8–10] This assay has also been modified to convert primary amines to thiols via 2-iminothiolane (ITL/Traut's reagent) to be utilized in the Cu^{+}/BCA assay (Figure S1.2, Step 0.)[12]

For quantification of the PC-amine beads, 150 µL from the synthesized nucleophile bead stock were incubated with 4 mM of ITL for 1 hour in pH 7.6 phosphate buffer in triplicate. The bead samples were then centrifuged at 8000xg for 5 minutes and washed five times with 1.5 mL of Milli-Q water to remove any unreacted ITL. Then, both the amine and thiol beads were incubated with 50:1 v/v BCA reagent A (25.75 mM BCA and 8.25 mM sodium tartrate in pH 11.3 carbonate buffer) and BCA reagent B (160.21 mM $CuSO_4 \cdot 5H_2O$) for 30 minutes in a 60°C water bath. A set of bead controls—one with beads without BCA reagent A and one without BCA reagent B—were also incubated at these conditions to ensure only absorbance signals originating from the Cu^{+}/BCA adducts were contributing to the quantitative determination of the thiol concentration. After the incubation, the solutions were immediately placed in an ice bath to prevent degradation of the Cu^{+}/BCA adducts. The absorbance of the supernatant was immediately recorded at 562 nm on a Shimadzu UV-1800 UV-Vis Spectrophotometer in a 0.3 cm quartz cuvette, and all samples were run within 15 minutes. Rerunning samples after 15 minutes showed no significant signs of degradation of the Cu^{+}/BCA adducts. The control without BCA reagent A showed negligible absorption, but the control without BCA reagent B exhibited a slight signal that was used for background subtraction. Additionally, an external calibration curve of 0-140 µM N-acetyl-l-cysteine (NAC) was incubated with BCA reagents A&B at the same conditions specified above to represent the absorption response from known thiol concentrations (Figure S1.3).

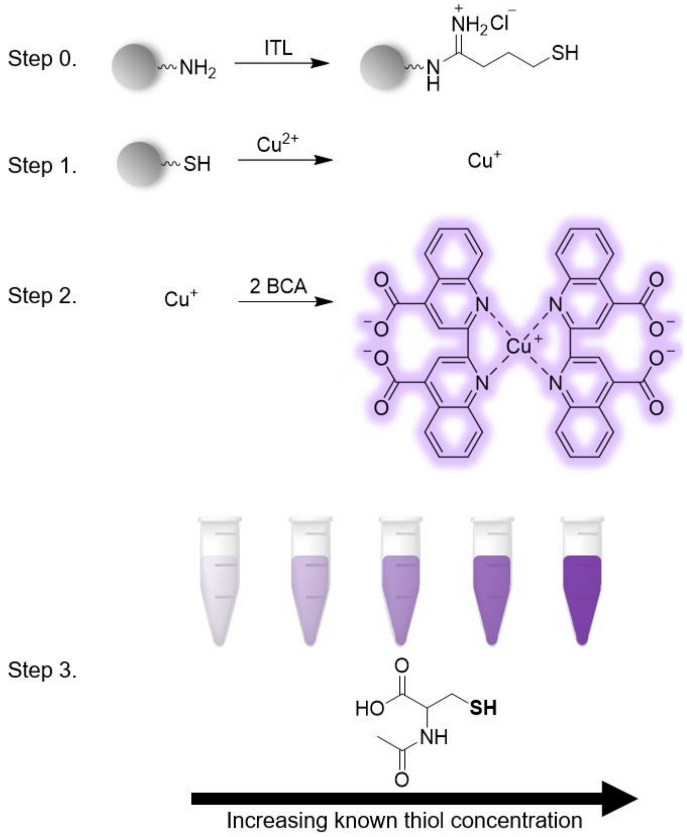

Figure S1.2. Schematic of the abbreviated Cu^{+}/BCA thiol quantification assay. Step 0 (for amine beads only): conversion of amines to thiols via 2-iminothiolane hydrochloride (ITL). Step 1: Reduction of Cu^{2+} to Cu^{+} by free thiols on the beads. Step 2: Production of chromophore for UV-visible absorbance measurements via 1:2 Cu^{+}:bicinchoninic acid (BCA). Step 3: Comparison of unknown bead thiol signals to an external calibration curve of known thiol concentrations (via n-acetyl-l-cysteine).

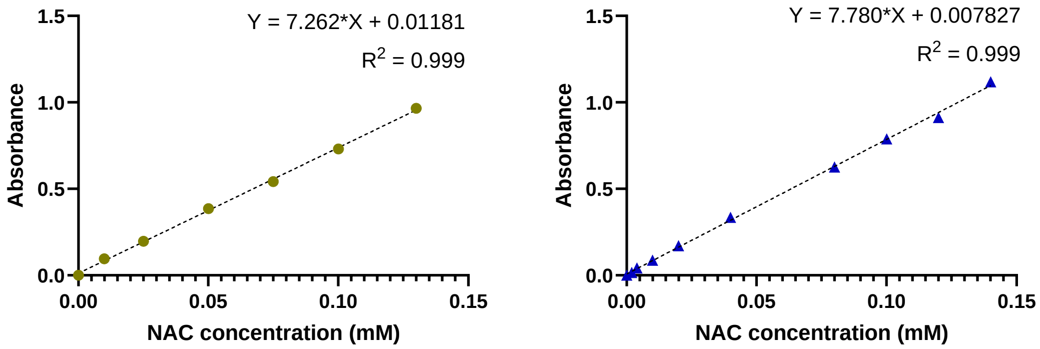

Figure S1.3. External calibration curves of the Cu^+/BCA assay using known n-acetyl-l-cysteine (NAC) concentrations for the amine bead (right) and thiol bead (left) nucleophile quantification.

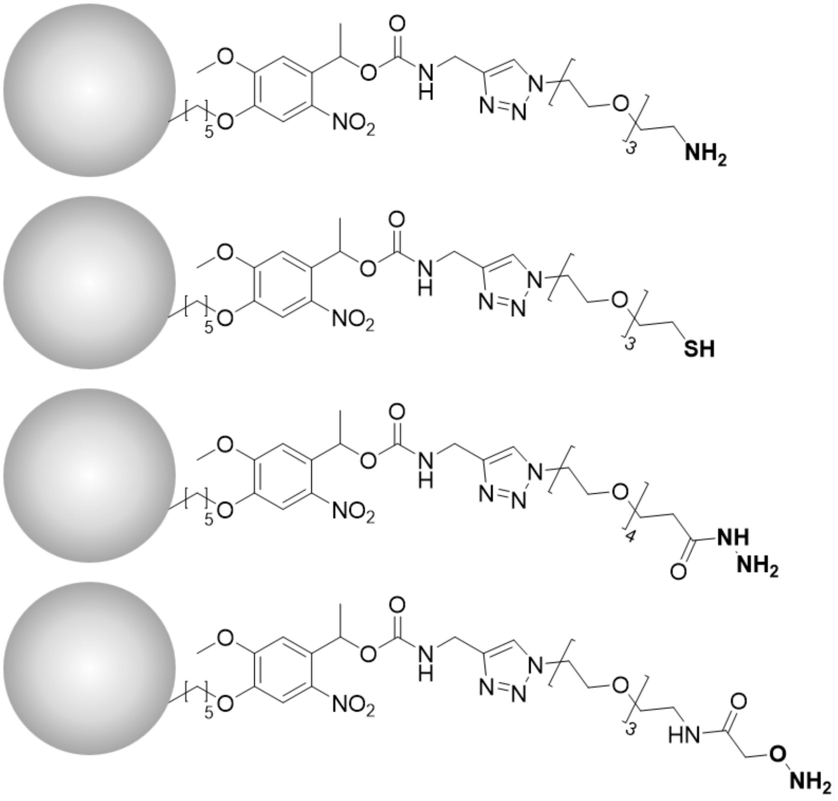

Figure S1.4. Structures of all synthesized nucleophile-labeled bead systems. Nucleophile bead systems are depicted as the following from top to bottom, with the reactive nucleophiles denoted in bold: amine, thiol, hydrazide, and aminooxy. The photocleavable linker is used for these depictions.

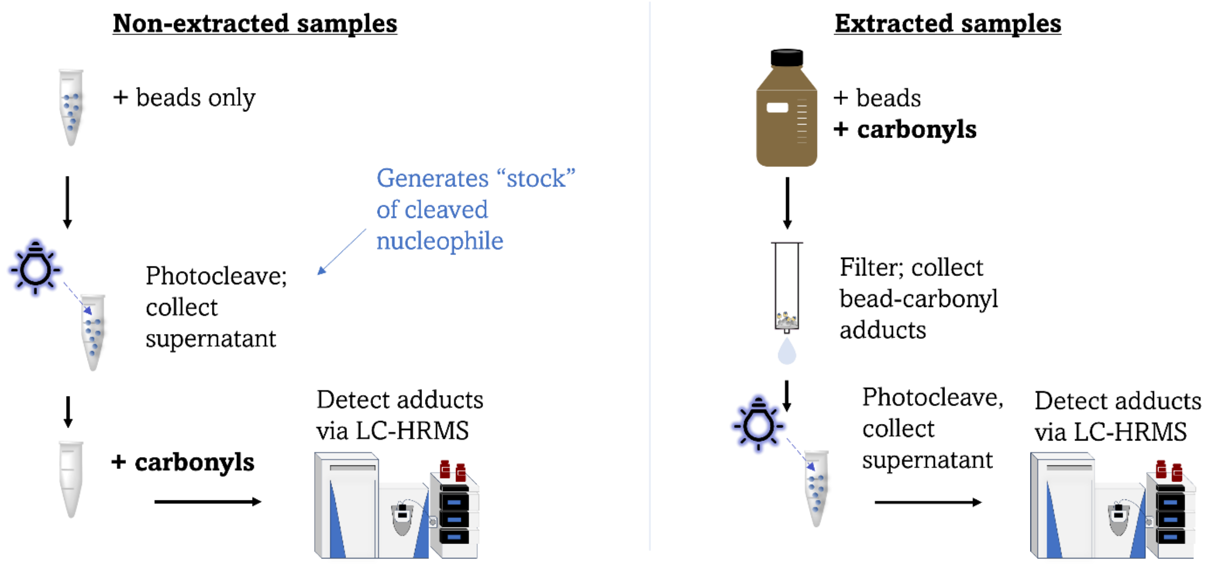

Figure S1.5. Experimental procedures used to analyze recovery of the carbonyl adducts with (right) and without (left) extraction.

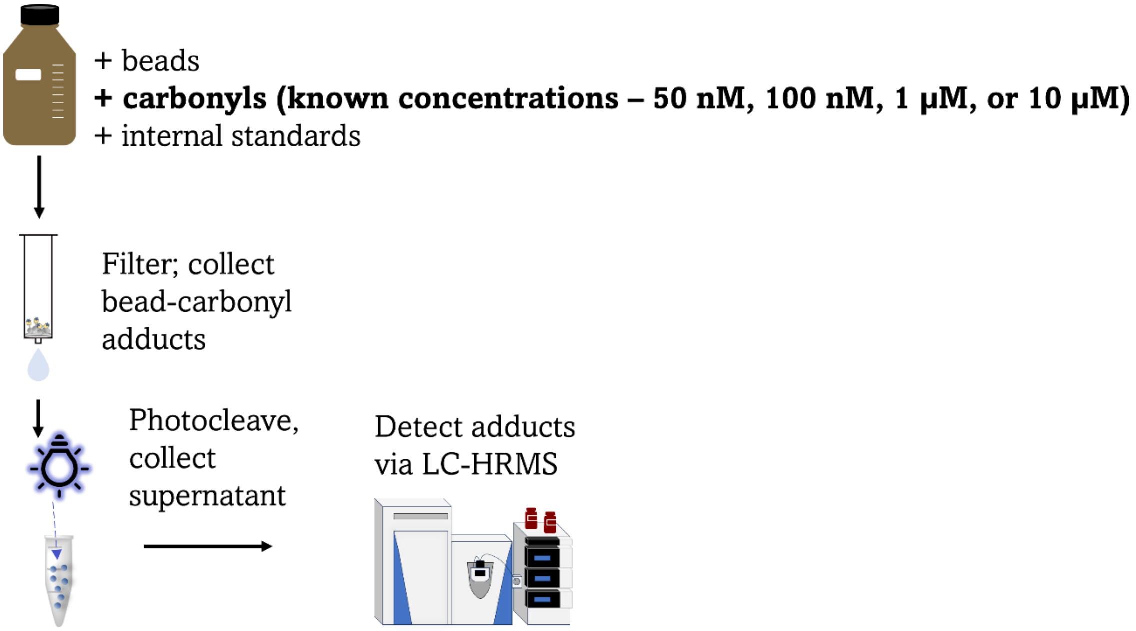

Figure S1.6. Experimental procedures used to analyze the accuracy of the carbonyl quantification.

2-butenedial (earlier RT)

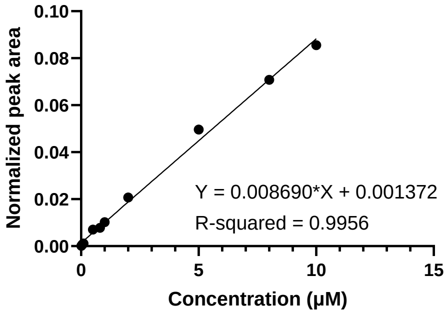

2-butenedial (later RT)

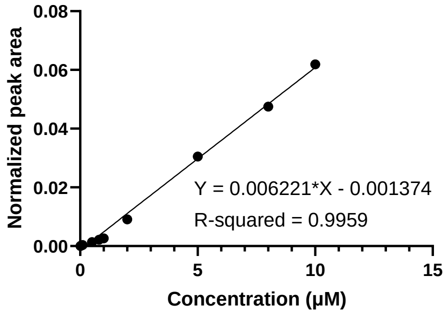

acrolein

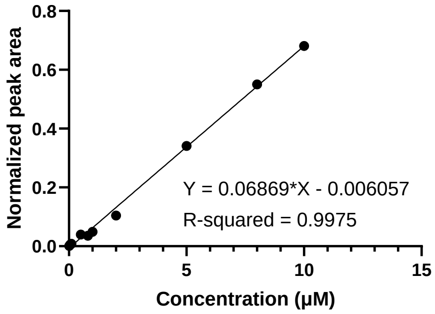

3-methylcrotonaldehyde

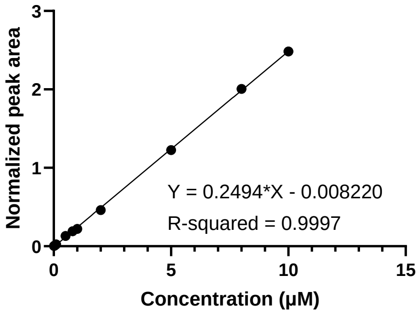

benzaldehyde

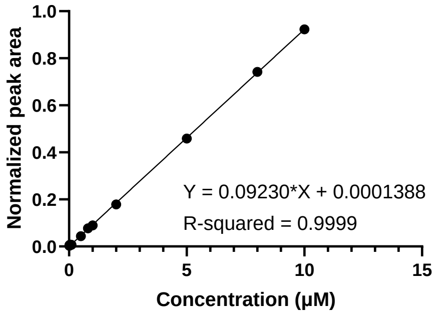

butanal

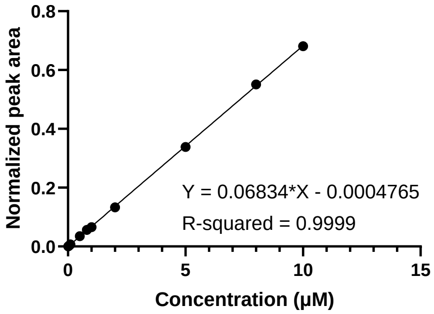

crotonaldehyde/2-methacrolein/methyl vinyl ketone

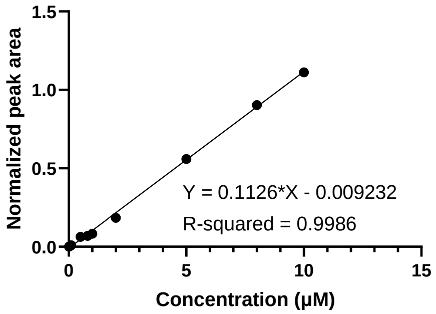

furaldehyde

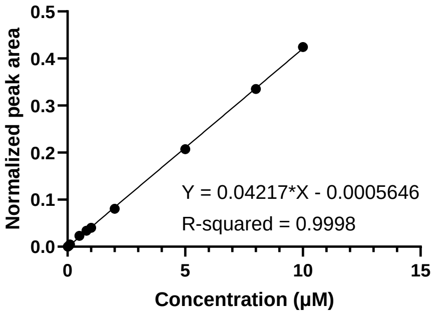

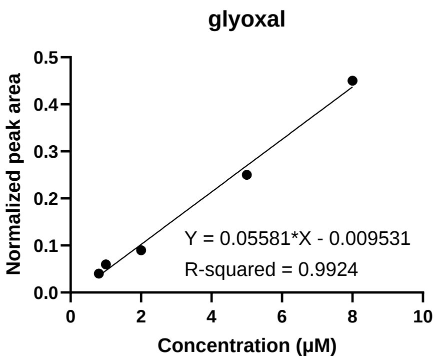
glyoxal
Normalized peak area
Concentration (μM)
Y = 0.05581*X - 0.009531
R-squared = 0.9924

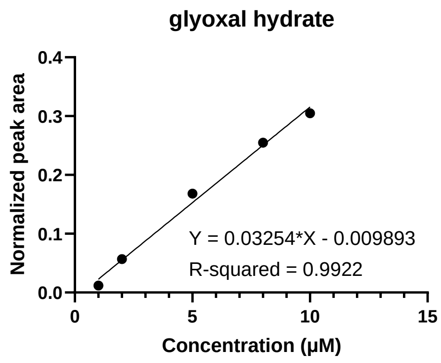
glyoxal hydrate
Normalized peak area
Concentration (μM)
Y = 0.03254*X - 0.009893
R-squared = 0.9922

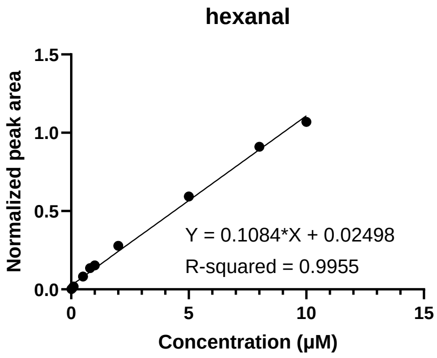
hexanal
Normalized peak area
Concentration (μM)
Y = 0.1084*X + 0.02498
R-squared = 0.9955

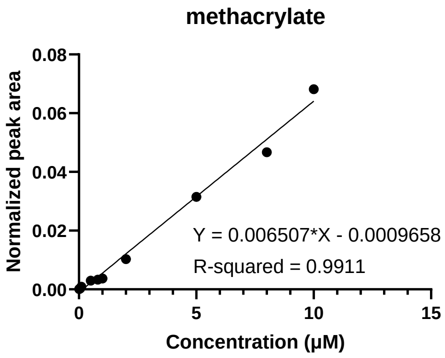
methacrylate
Normalized peak area
Concentration (μM)
Y = 0.006507*X - 0.0009658
R-squared = 0.9911

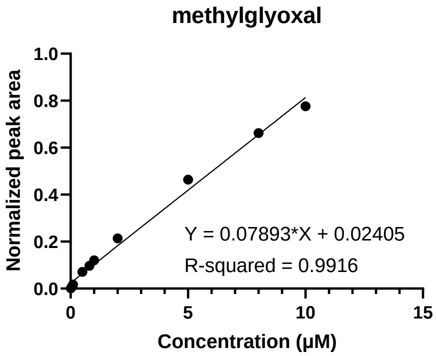
methylglyoxal
Normalized peak area
Concentration (μM)
Y = 0.07893*X + 0.02405
R-squared = 0.9916

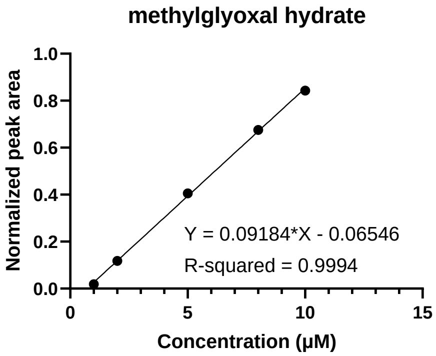
methylglyoxal hydrate
Normalized peak area
Concentration (μM)
Y = 0.09184*X - 0.06546
R-squared = 0.9994

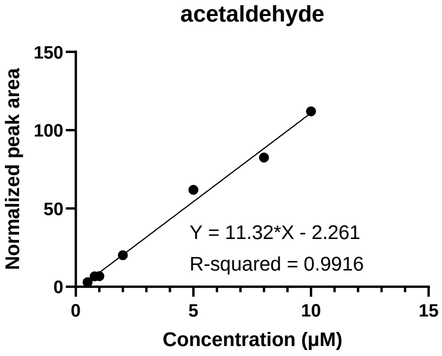
acetaldehyde
Normalized peak area
Concentration (μM)
Y = 11.32*X - 2.261
R-squared = 0.9916

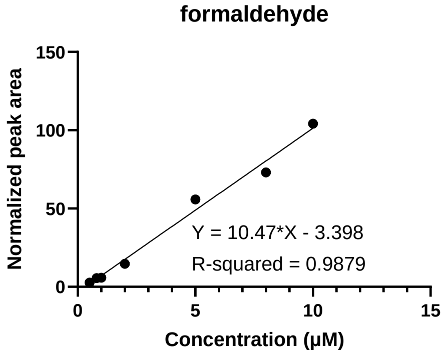
formaldehyde
Normalized peak area
Concentration (μM)
Y = 10.47*X - 3.398
R-squared = 0.9879

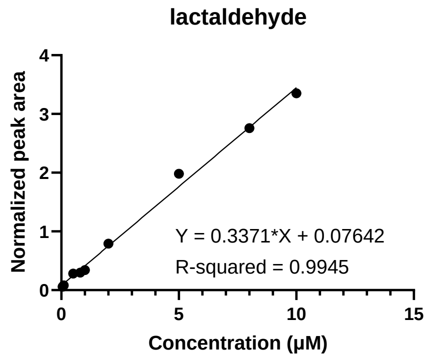
lactaldehyde
Normalized peak area
Concentration (μM)
0
1
2
3
4
0
5
10
15
Y = 0.3371*X + 0.07642
R-squared = 0.9945

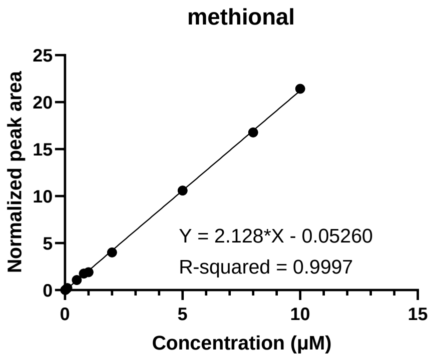
methional
Normalized peak area
Concentration (μM)
0
5
10
15
20
25
0
5
10
15
Y = 2.128*X - 0.05260
R-squared = 0.9997

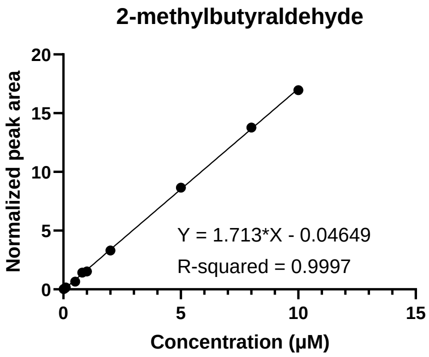
2-methylbutyraldehyde
Normalized peak area
Concentration (μM)
0
5
10
15
20
0
5
10
15
Y = 1.713*X - 0.04649
R-squared = 0.9997

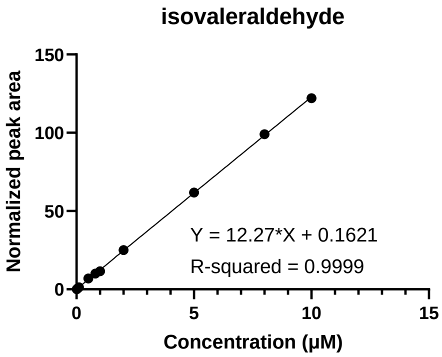
isovaleraldehyde
Normalized peak area
Concentration (μM)
0
50
100
150
0
5
10
15
Y = 12.27*X + 0.1621
R-squared = 0.9999

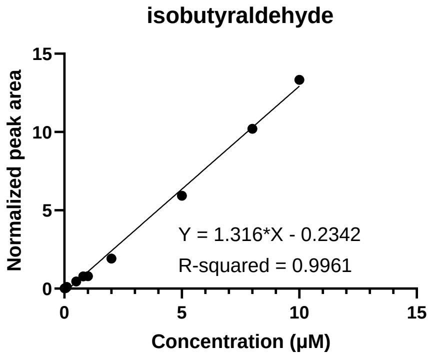
isobutyraldehyde
Normalized peak area
Concentration (μM)
0
5
10
15
0
5
10
15
Y = 1.316*X - 0.2342
R-squared = 0.9961

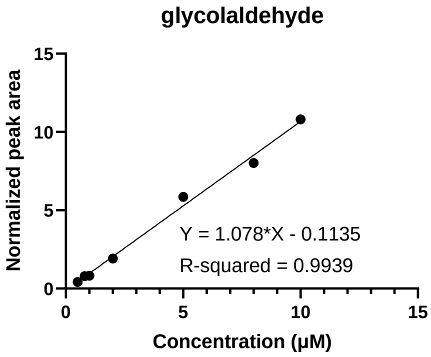
glycolaldehyde
Normalized peak area
Concentration (μM)
0
5
10
15
0
5
10
15
Y = 1.078*X - 0.1135
R-squared = 0.9939

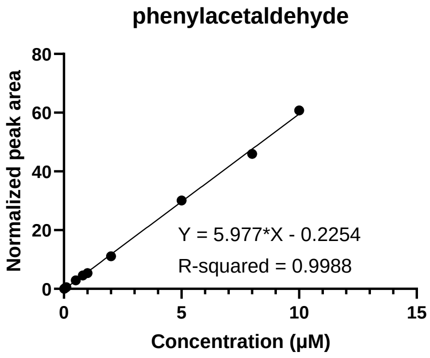
phenylacetaldehyde
Normalized peak area
Concentration (μM)
0
20
40
60
80
0
5
10
15
Y = 5.977*X - 0.2254
R-squared = 0.9988

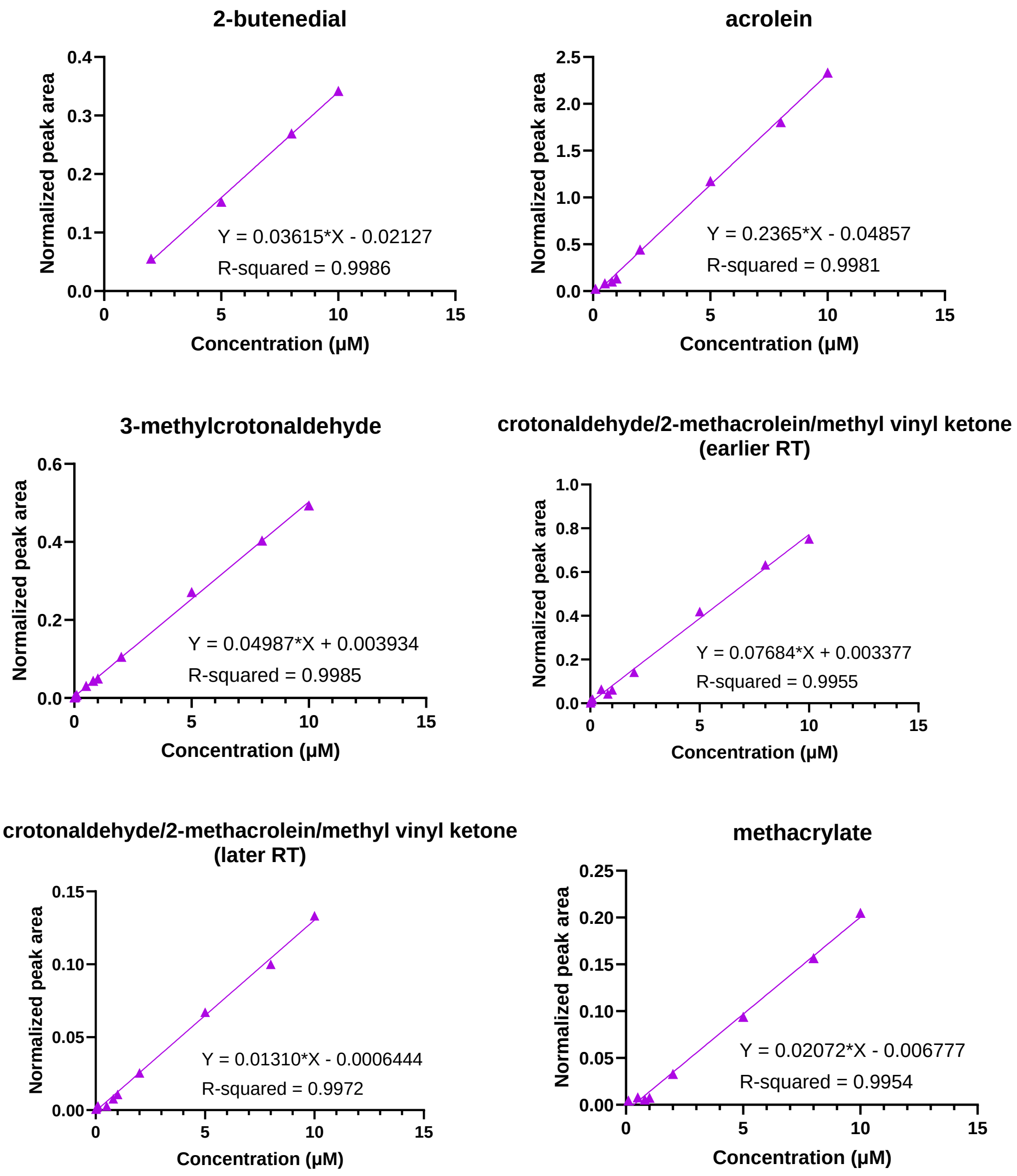

Figure S1.7. Calibration curves for carbonyl quantitative analysis. Calibrants were selected to restrict curves to their linear ranges. All carbonyl adduct peak areas were normalized against the benzaldehyde-d5 internal standard peak area except for butanal and hexanal, which were normalized using the butanal-d2 internal standard. Schiff base adducts are depicted by graphs using black circles, while Michael addition adducts for applicable carbonyls are depicted by purple triangles. RT = retention time. All data are from the PC-ONH_2 bead system.

Text S1.4. Determination of linear ranges, limits of detection (LOD), limits of quantification (LOQ), and discussion of background carbonyl concentrations

High background presence of carbonyls in negative controls is a known challenge in carbonyl quantification studies in environmental matrices.[13–17] Previous studies have attempted to identify the source of contamination, but the identities of the sources remain widely variable or unknown. One study found that formaldehyde concentrations increased overtime when sitting in an LC-MS autosampler tray, and this contamination was speculated to originate from laboratory air.[16] A different study used LC/MS grade water for all stocks and samples, used a new LC column, prepared the derivatizing agent in a different hood than carbonyl stock solutions, and switched their LC mobile phase additive from formic acid to acetic acid; all of these changes did not have an impact on background formaldehyde concentrations.[14] They attributed their formaldehyde background to increasing *p*-toluenesulfonyl hydrazide concentrations.[14]

In this study, while the intensity of the background signals varied between each carbonyl (e.g., typically low for nonpolar carbonyls with higher molecular weights and higher for more polar carbonyls with low molecular weights), there was typically background signal in all negative controls. Because of this, signal-to-noise ratios could not be used for determining the limit of detection (LOD) or quantification (LOQ) for the carbonyl adducts. Instead, the LOD for each carbonyl was defined as the lowest calibrant concentration whose signal was 1) higher than the negative control without spiked carbonyl and 2) followed by an increasing trend for each subsequent calibrant (Tables S1.1-2). Therefore, the lowest LOD that was tested in this study was 0.01 µM (i.e., the lowest calibrant). The LOQ was defined as the lowest spiked control with the lowest concentration accurately (70-130%) quantified (see main text, "Accuracy experiments").

Table S1.2. LODs for the Schiff base carbonyl adducts in the PC-ONH_2 bead system.

CARBONYL	LOD (µM)
**2-butenedial (earlier RT)**	0.01
**2-butenedial (later RT)**	0.01
**3-methylcrotonaldeyde**	0.01
**acrolein**	0.01
**acrylamide**	>10
**benzaldehyde**	0.01
**butanal**	0.01
**crotonaldehyde/2-methacrolein/methyl vinyl ketone (isomers)**	0.01
**furaldehyde**	0.01

**glyoxal**	0.8
**glyoxal hydrate**	1
**hexanal**	0.01
**methacrylate**	0.01
**methylglyoxal**	0.01
**methylglyoxal hydrate**	1
**formaldehyde**	0.5
**acetaldehyde**	0.5
**lactaldehyde**	0.05
**methional**	0.01
**2-methylbutyraldehyde**	0.01
**isovaleraldehyde**	0.01
**glycolaldehyde**	0.5
**phenylacetaldehyde**	0.01

Table S1.3. LODs for the Michael addition carbonyl adducts in the PC-ONH_2 bead system.

CARBONYL	LOD (μM)
**2-butenedial**	2
**3-methylcrotonaldeyde**	0.01
**acrolein**	0.1
**crotonaldehyde/2-methacrolein/methyl vinyl ketone (isomers; earlier RT)**	0.01
**crotonaldehyde/2-methacrolein/methyl vinyl ketone (isomers; later RT)**	0.01
**methacrylate**	0.1

Table S1.4. Structures of all amino acids tested in this study, along with the expected aldehyde formation based on known transformation mechanisms.[18,19]

**Amino acid**		**Aldehyde**	
L-alanine	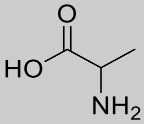	acetaldehyde	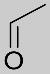
L-glycine	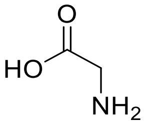	formaldehyde	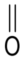
L-isoleucine	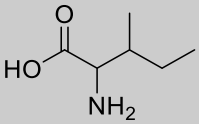	2-methylbutyraldehyde	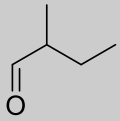
L-leucine	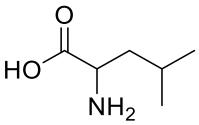	isovaleraldehyde	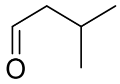
L-methionine	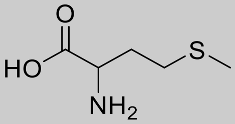	methional	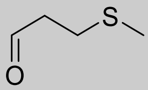
L-phenylalanine	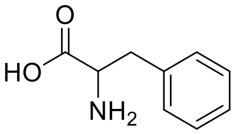	phenylacetaldehyde	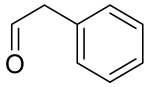
L-serine	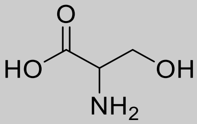	glycolaldehyde	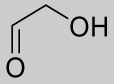
L-threonine	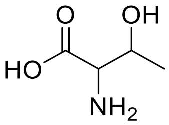	lactaldehyde	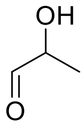
L-valine	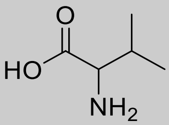	isobutyraldehyde	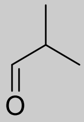

Text S1.5. LC-HRMS analysis

An RSLC3000 ultra-high-performance liquid chromatography system coupled with a Thermo Scientific Q Exactive HF high-resolution mass spectrometer (LC-HRMS) equipped with a heated-electrospray ionization (H-ESI) source was utilized for the detection of all derivatized carbonyl compounds. For the LC system, separations were performed with a Phenomenex Synergi Hydro-RP column (4 um, 80 *Å*, 1 x 150 mm) held at 30 °C. The flow gradient was set as the following, using starting mobile phase conditions of 100% 0.1% (v/v) formic acid in Milli-Q water (A) and 0% methanol (B): for the first 3 minutes, these conditions were held constant, then B was increased linearly to 95% from 3 to 8 minutes. From 8 to 14 minutes, these conditions were held constant before B was decreased linearly back down to 0% at 14.1 min, where it was held until the end of the run at 28 minutes. LC flow was diverted to the MS from 5-27 minutes. The flow rate was constant at 75 µL/min, and sample injection volumes were set at 10 µL.

For the MS analysis, positive ionization mode was used for all derivatized carbonyl detection, and weekly external mass calibration was performed to ensure high mass accuracies in procedures described elsewhere.[20] For the MS settings, ESI source parameters included the following: sheath gas flow rate = 20 arbitrary units (AU), aux gas flow rate = 5 AU, spray voltage = 3.5 kV (positive), capillary temperature = 250 °C, S-lens RF level = 60; aux gas heater temperature = 100 °C. The MS full scan/data-dependent (dd)-MS^2 setting was utilized with the full scans ranging from 50 – 750 *m/z* for the preliminary nucleophile probe testing experiments and 290-750 *m/z* for the remaining experiments (resolution = 120,000, target automatic gain control (AGC) = 3×10^6, maximum inject time = 100 ms). For the dd-MS^2 scans, the settings were as follows: resolution = 60,000, AGC = 1×10^5, isolation window = 1.0 *m/z*, stepped normalized collision energies = 10, 30, and 50%, inject time = 50 ms, data dependent threshold = 2.0×10^4, loop count = 5.

Text S1.6. Nucleophile probe testing: comparing the cleavable linkers

Photocleavable (PC) versus chemically cleavable (Dde) linkers. To ensure that the microbead systems were compatible with LC-HRMS analysis, we needed to verify the nucleophile adducts were successfully cleaved from the solid-phase beads and were detectable via LC-HRMS. Both PC- and Dde-cleavable linkers were tested, using amines as the nucleophile probe. An intense signal was detected for the free nucleophile after photocleavage from the bead using the PC linker, indicating the CuAAC click chemistry synthesis and cleavage step were successful (optimization for carbonyl derivatization is discussed in the main text). Therefore, the beads were successfully synthesized and demonstrated effective photocleavage of the nucleophile from the solid-phase beads to enable LC-HRMS analysis. In contrast, results from the Dde-bead system indicated that the synthesis and/or cleaving steps were unsuccessful. No carbonyl-amine adducts were detected in the Dde-bead systems, with only a weak signal present for the free amine nucleophile. Possible explanations for the lack of success with the Dde-bead systems could include: 1) the 2% hydrazine (i.e., a nucleophilic diamine) cleaving solution competed with amines immobilized on the beads for the carbonyls interactions, and/or 2) the Dde-bead synthesis (or cleavage) was unsuccessful at the tested conditions to produce an excess of amine beads (or cleave the amine adducts from the beads). From these results, the PC-beads were chosen for the SPREx assay experiments in this work.

Text S1.7. Nucleophile probe testing: thiol probe results & discussion

A derivatization issue appeared to be present in the thiol bead system, making it insufficient for detection of environmentally relevant concentrations of OBPs. Although thiols are known to undergo Michael addition reactions with α,β-unsaturated carbonyls to form stable adducts,[21,22] only a response for the non-derivatized thiol adduct was present in the LC chromatograms. No carbonyl-thiol adducts were detected in the LC-HRMS analysis. One speculation behind the poor performance of the thiol beads was thought to be disulfide bond formation preventing free thiols from interacting with the carbonyls. However, the introduction of TCEP to reduce any disulfide bonds did not improve the assay performance. Therefore, the aminooxy beads were still chosen for all subsequent SPREx experiments, with the optimization of the thiol bead reactions to be determined in future work.

Text S1.8. Nucleophile probe testing: NH_2 and reduced NH_2 systems

Impact of reducing agents on amine probes. Amines are a toxicologically meaningful candidate to use as an *in chemico* toxicity probe in SPREx. Lysine and DNA bases are abundant with primary amine groups, and these nucleophilic biomolecules serve as targets for electrophiles in the molecular initiating events that cascade into adverse health outcomes.[23,24] As a derivatization agent, however, amines can be ineffective in aqueous conditions due to the reversibility of the amine-carbonyl products (known as Schiff bases or imines) when water interacts with the imine's carbon-nitrogen double bond (Figure S1.8).[25,26] This was observed in the LC-HRMS analysis where most PC-amine bead carbonyl adducts were not detected, and, for the few adducts that were detected, peaks had low intensities and poor shapes. Increasing the pH from 7 to 9 had negligible impacts on the responses (data not shown). Only 2-butene-1,4-dial (Schiff base product) yielded a strong peak signal and Gaussian shape, likely due to the unique structural advantage of being able to form a stable ring adduct rather than an unstable imine chain (Figure S2.60).[1]

To address the instability issue of the amine bead system, borohydride reducing agents were tested to convert the Schiff base products from reversible, unstable carbon-nitrogen double bonds to irreversible, stable single bonds. Both sodium triacetoxyborohydride and sodium cyanoborohydride have been used previously in the literature for the reduction of imines.[26,27] In our experiments, only sodium cyanoborohydride showed an improvement of carbonyl-amine adduct signals in the SPREx assay. With the reducing agent, 1,4-benzoquinone (*m/z* 366), butanal (*m/z* 330), glyoxal (*m/z* 316), hexanal (*m/z* 358), and methylglyoxal (*m/z* 330) showed marked improvement in detection (Figures S2.62-68; all expected carbonyl adducts can be found in Table S1.5 with their rounded *m/z* values, product type, and target nucleophile). However, the Schiff base adducts for acrylamide, crotonaldehyde, 2-methacrolein, methyl vinyl ketone, furaldehyde, methacrylate, 3-methylcrotonaldehyde, and 2,6-dichloro-1,4-benzoquinone were still not detectable. Comparing the detectable vs. nondetectable carbonyls with the reduced amine bead system, it appears as though this nucleophile system is not able to capture most of the α,β-unsaturated carbonyls, even though Schiff base products are possible with this functional class (Table S1.5).[23,28] In addition to not capturing the majority of the carbonyls tested in the mixture, the signal responses for the detected carbonyls were still too weak to be practically used for environmentally relevant concentrations.

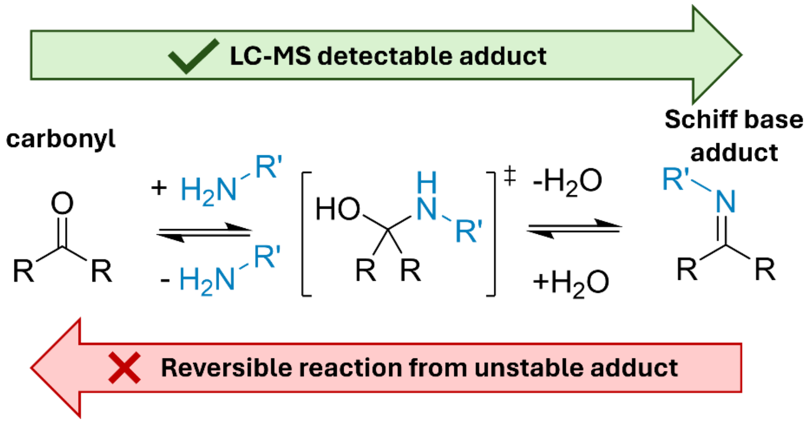

Figure S1.8. Schiff base adduct formation from carbonyl + amine reactions and instability in water.

Table S1.5. Expected carbonyl adducts in the SPREx assay method development. Carbonyls are distinguished by their structural classification (e.g., saturated versus unsaturated (di)carbonyls, amides, esters, ketones), product type (i.e., Schiff base or Michael addition adducts), presence of a hydrate for dicarbonyls, and the rounded *m/z* values when derivatized by the $-NNH_2$, $-ONH_2$, or reduced $-NH_2$ bead systems. Crotonald = crotonaldehyde, 2MA = 2-methacrolein, MeVK = methyl vinyl ketone; these isomers were not chromatographically distinguishable. The $-NH_2$ bead adducts shown in the table were all reduced imine products via sodium cyanoborohydride (e.g., Michael addition products were not applicable (n/a)). (*) indicates the carbonyl adduct was not detected in this system.

Carbonyl	Class	Product type	Hydrate?	NNH_2 *m/z*	ONH_2 *m/z*	NH_2 (reduced) *m/z*
Acrolein	α,β-unsaturated	Schiff base	No	399	385	314*
Acrolein	α,β-unsaturated	Michael addition	No	417	403	332*
Acrylamide	α,β-unsaturated amide	Schiff base	No	414*	400	329*
Benzaldehyde	saturated	Schiff base	No	449	435	364*
Butanal	saturated	Schiff base	No	415	401	330
2-butenedial (earlier RT)	α,β-unsaturated	Schiff base	No	427	413	342
2-butenedial (later RT)	α,β-unsaturated	Schiff base	No	427	413	n/a
2-butenedial	α,β-unsaturated	Michael addition	No	445	431*	360*
Crotonald/2MA/MeVK	α,β-unsaturated (MeVK = ketone)	Schiff base	No	413	399	328*

**Crotonald/2MA/MeVK**	**α,β-unsaturated (MeVK = ketone)**	**Michael addition**	**No**	431	417	346*
**Furaldehyde**	**saturated**	**Schiff base**	**No**	439	425	354*
**Glyoxal**	**saturated dicarbonyl**	**Schiff base**	**No**	401	387	316
**Glyoxal**	**saturated dicarbonyl**	**Schiff base**	**Hydrate**	419	405	334*
**Hexanal**	**saturated**	**Schiff base**	**No**	443	429	358
**Methacrylate**	**α,β-unsaturated ester**	**Schiff base**	**No**	443	429	358*
**Methacrylate**	**α,β-unsaturated ester**	**Michael addition**	**No**	461	447	376*
**Methylglyoxal**	**saturated dicarbonyl**	**Schiff base**	**No**	415	401	330
**Methylglyoxal**	**saturated dicarbonyl**	**Schiff base**	**Hydrate**	433	419	348*
**1,4-benzoquinone**	**α,β-unsaturated dicarbonyl (ketone)**	**Schiff base**	**No**	451*	437	366
**3-methylcrotonaldehyde**	**α,β-unsaturated**	**Schiff base**	**No**	427	413	342*
**3-methylcrotonaldehyde**	**α,β-unsaturated**	**Michael addition**	**No**	445	431	360*

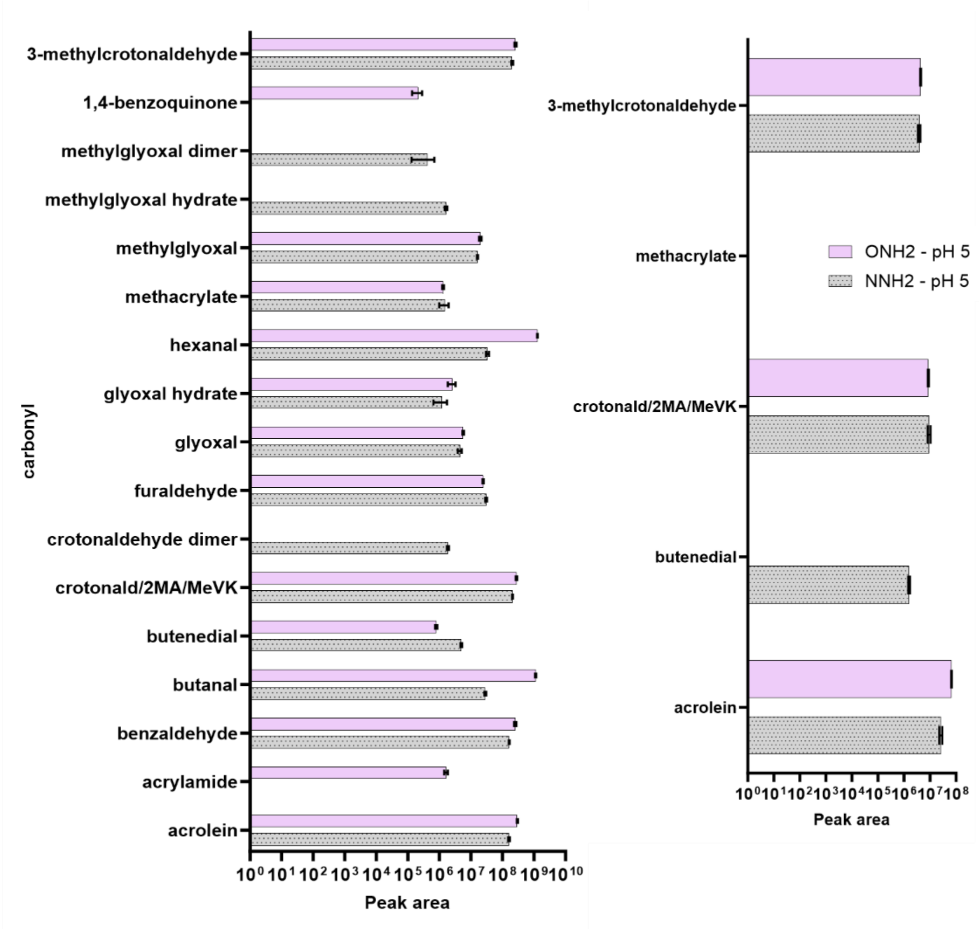

Figure S1.9. Peak areas (log_{10} scale) for all carbonyl adducts detected in the ONH_2 bead system (purple) and NNH_2 bead system (gray) reacted at pH 5. Schiff base adducts are depicted in the left panel, while the Michael addition adducts are depicted in the right panel. Crotonald = crotonaldehyde, 2MA = 2-methacrolein, MeVK = methyl vinyl ketone (isomers).

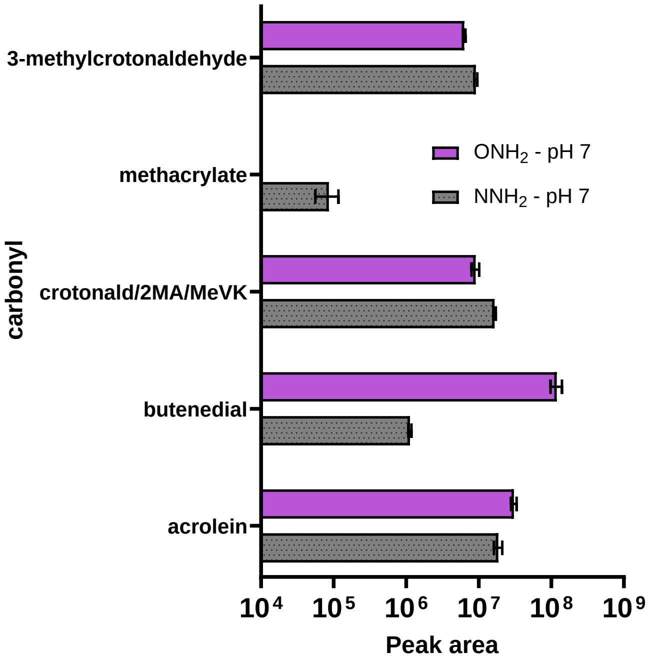

Figure S1.10. Peak areas (log_{10} scale) for all Michael addition carbonyl adducts applicable for α,β-unsaturated carbonyls only. Adducts were detected in both the ONH_2 bead system (purple; top) and NNH_2 bead system (gray; bottom) reacted at pH 7. Crototonald = crotonaldehyde, 2MA = 2-methacrolein, MeVK = methyl vinyl ketone (isomers integrated together or not chromatographically distinguishable).

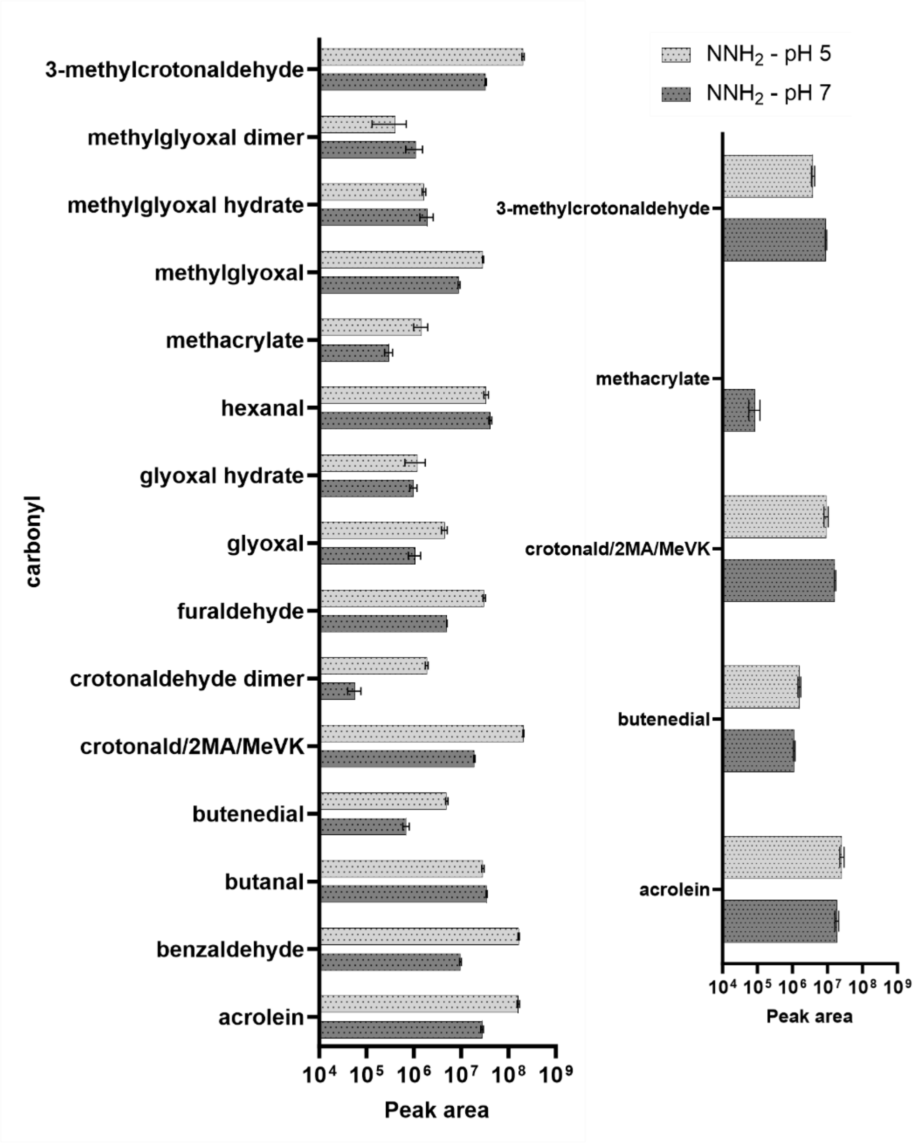

Figure S1.11. pH impact on carbonyl adduct peak areas (log_{10} scale) in the NNH_2 bead system for all carbonyls (Schiff base = left panel, Michael addition = right panel). Crotonald = crotonaldehyde, 2MA = 2-methacrolein, MeVK = methyl vinyl ketone (isomers).

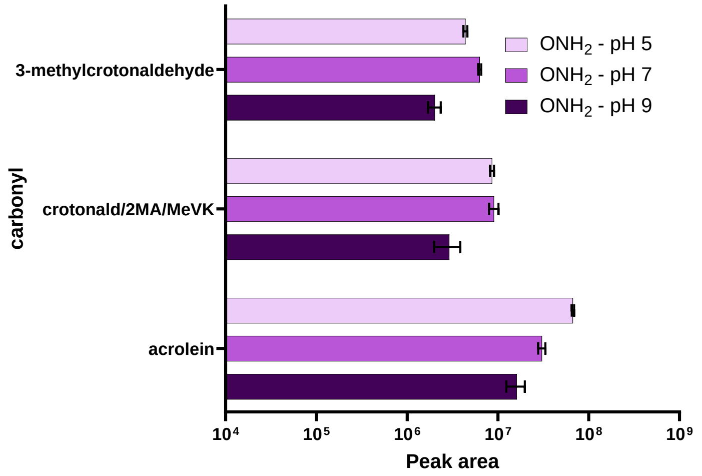

Figure S1.12. pH impact on carbonyl adduct peak areas (log_{10} scale) in the ONH_2 bead system for the Michael addition adducts for the α,β-unsaturated carbonyls. Crototonald = crotonaldehyde, 2MA = 2-methacrolein, MeVK = methyl vinyl ketone (isomers integrated together or not chromatographically distinguishable).

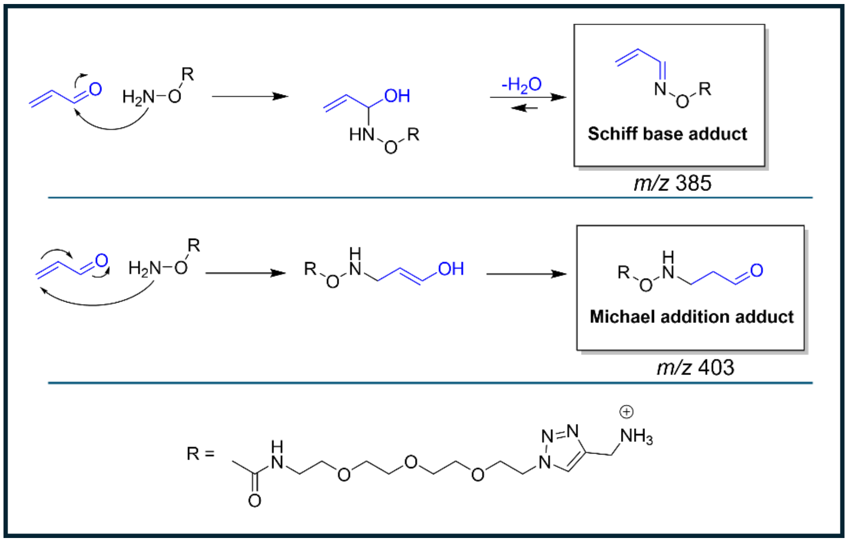

Figure S1.13. Illustration of the two potential adducts from derivatization with α, β-unsaturated carbonyls using acrolein (blue) derivatized with aminooxy beads as an example. Nucleophile attack can either occur at: 1) the carbonyl carbon to yield the Schiff base adduct (top; *m/z* 385) and/or 2) the β-carbon to yield the Michael addition adduct (bottom; *m/z* 403).

Text S1.9. Nucleophile probe testing: further discussion on the ONH_2 vs. NNH_2 bead systems

For the saturated (di)carbonyls at pH 7, NNH_2 slightly outperformed for detecting furaldehyde and methylglyoxal, while ONH_2 outperformed NNH_2 beads for benzaldehyde, butanal, glyoxal, and hexanal (Figure 2A, Figure S1.11). For the α, β-unsaturated dicarbonyls, no clear trend was observed for NNH_2 versus ONH_2 for detecting the Schiff base or Michael addition products. For instance, for acrolein at pH 7, the NNH_2 bead system had a greater signal for the Schiff base product (*m/z* 399 vs. *m/z* 385, Figure 2A), while the ONH_2 system had a greater signal for the Michael addition product (*m/z* 417 vs. *m/z* 403, Figure S1.11). The opposite trend was seen for the Schiff base and Michael addition products of 3-methylcrotonaldehyde (Figures 2A & S1.11).

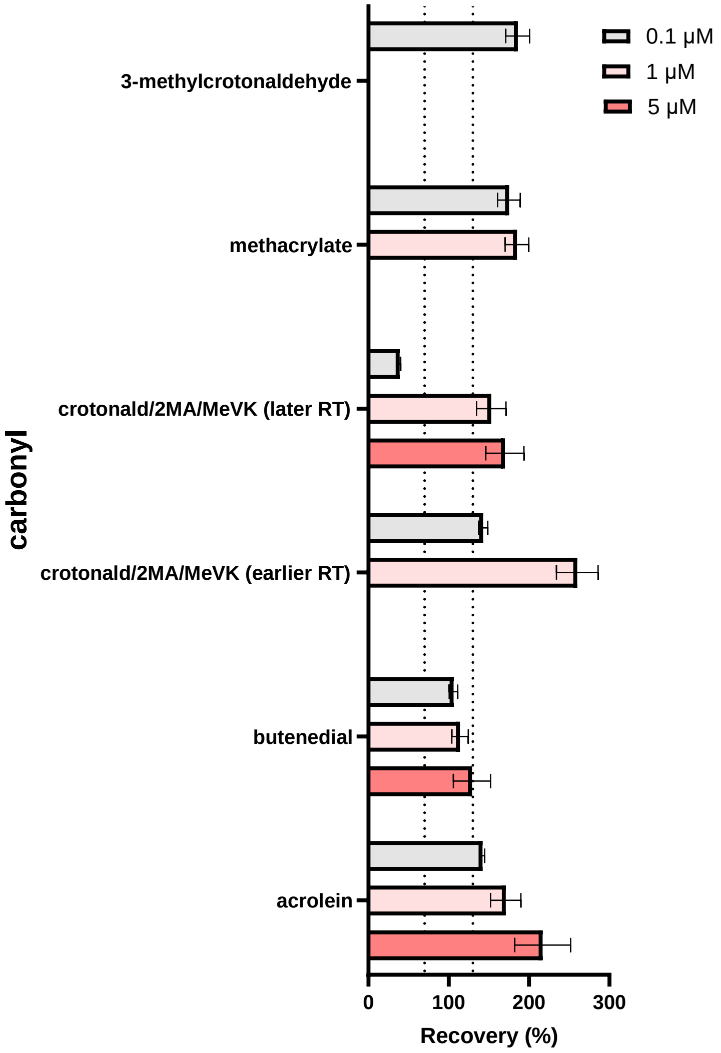

Figure S1.14. Recoveries (%) of Michael addition carbonyl adducts after undergoing the SPREx extraction protocol (using 0.1, 1, or 5 µM of each carbonyl). Dashed lines across the x-axis indicate the carbonyl adducts that fall within the acceptable range of 70-130% recovery. Values >300% were excluded. Values are reported as the averages of each extracted sample peak area divided by the average peak area of the non-extracted samples, with error bars indicating the standard deviation.

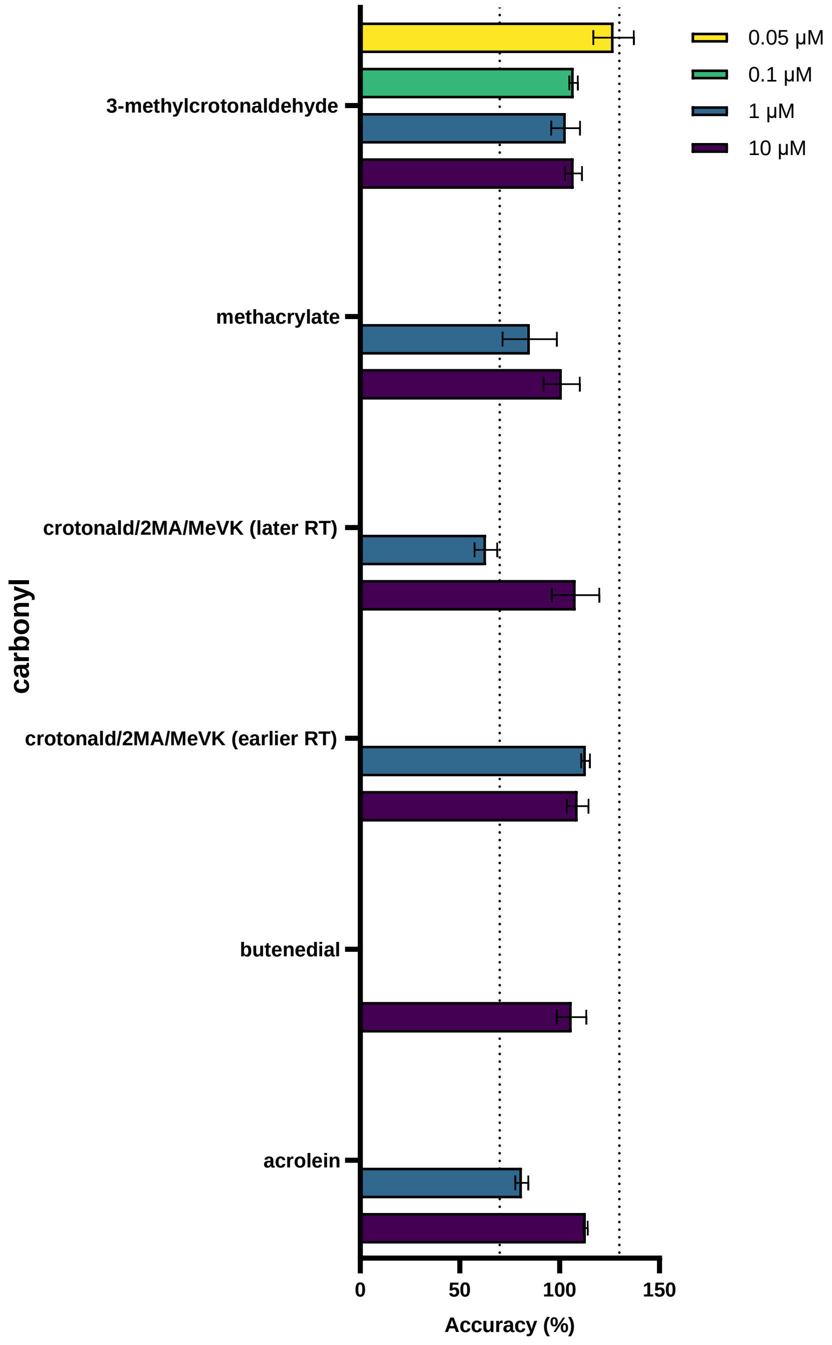

Figure S1.15. Accuracy (%) of quantification of the Michael addition carbonyls adducts tested in the nucleophile probe and recovery experiments at the different spiked control concentrations. Dashed lines indicate the signals that fell within the acceptable range of 70-130% accuracy. Missing values indicate the concentration was below the LOQ. Values are reported as the ratio (%) indicating the averages of the triplicate samples over the known spiked concentration with error bars indicating the standard deviation.

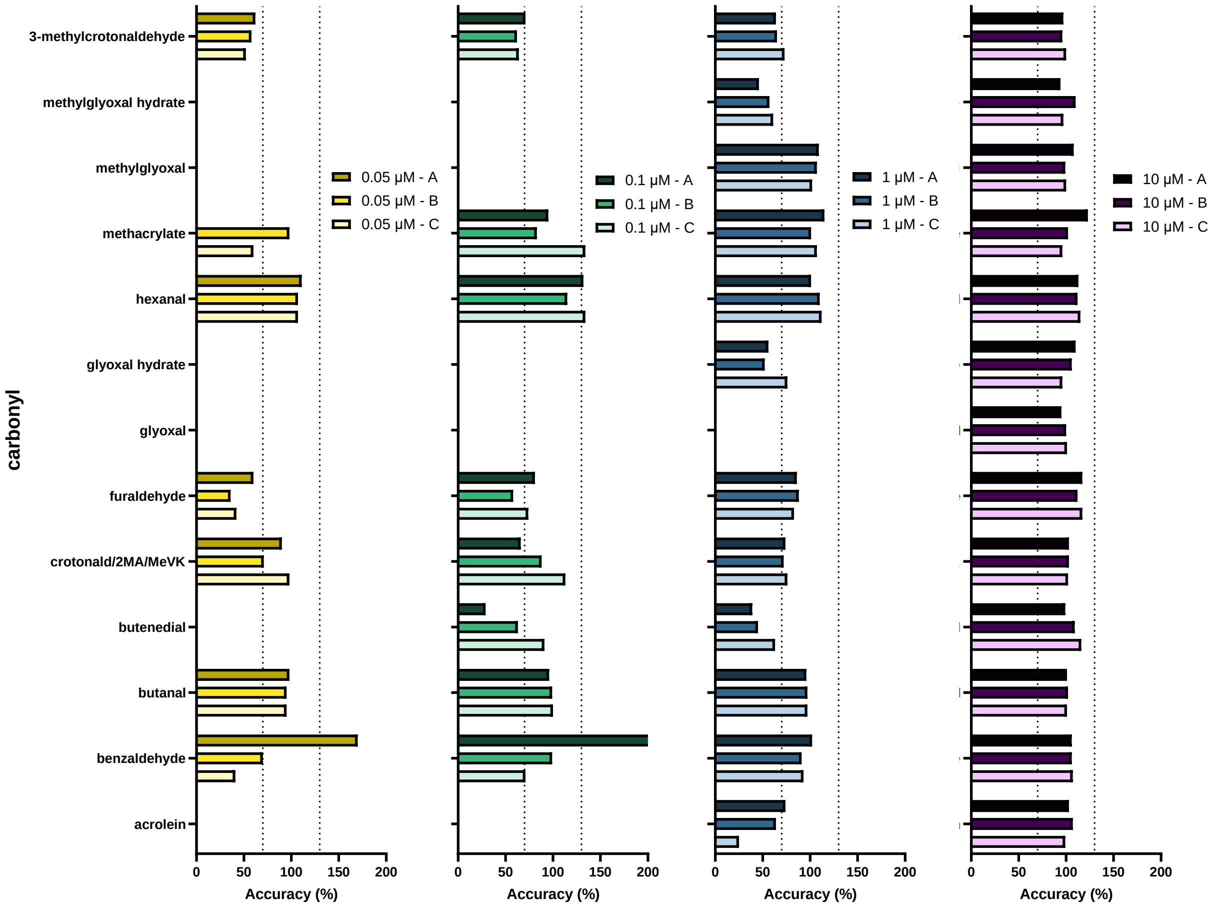

Figure S1.16. Stability experiments of triplicate spiked controls (0.05, 0.1, 1, and 10 µM of the Schiff base carbonyl adducts) injected during the beginning, middle, and end of a 72-hour LC-HRMS analysis (indicated in the legend by A, B, and C, respectively). Results from the Michael addition adducts are depicted in Figure S1.17.

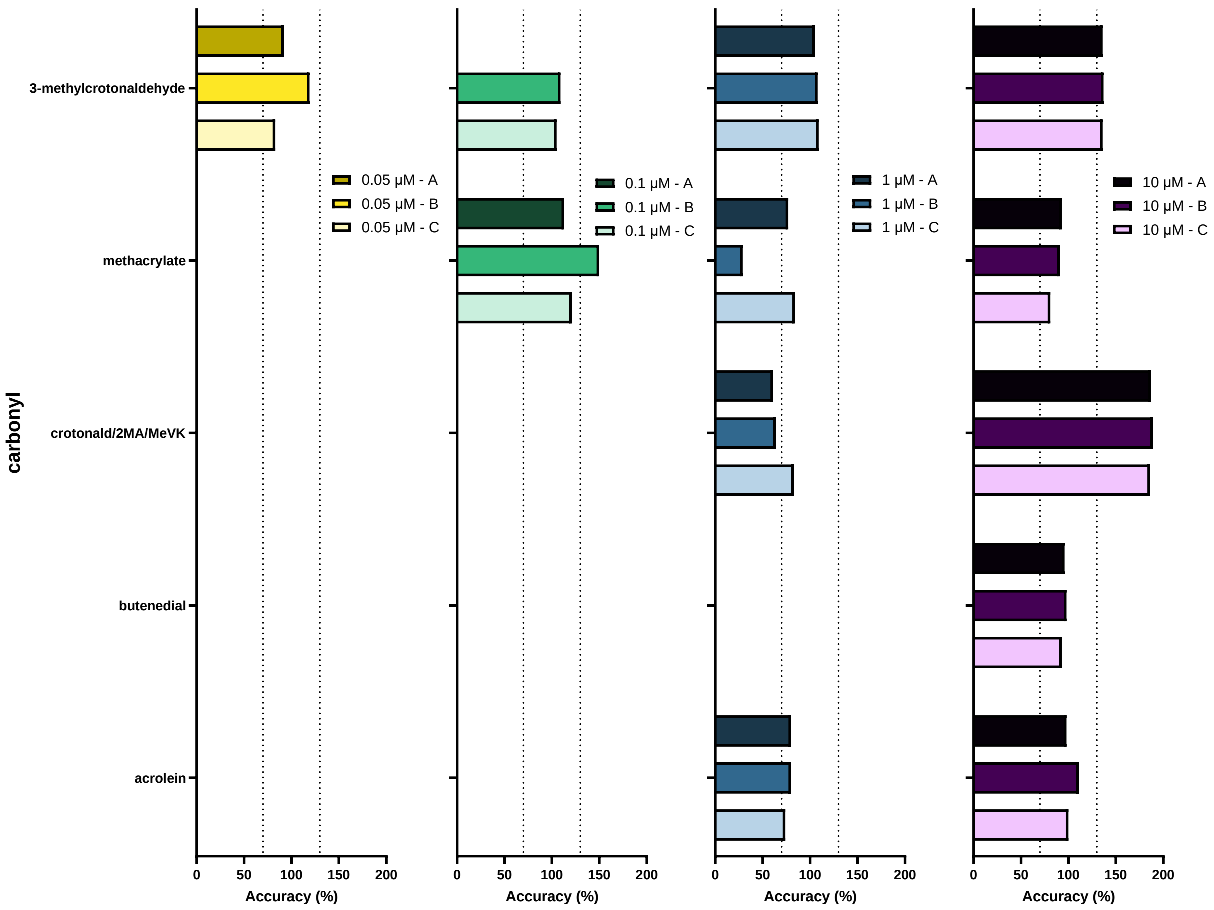

Figure S.17. Stability experiments of triplicate spiked controls (0.05, 0.1, 1, and 10 µM) of the Michael addition carbonyl adducts injected during the beginning, middle, and end of a 72-hour LC-HRMS analysis (indicated in the legend by A, B, and C, respectively).

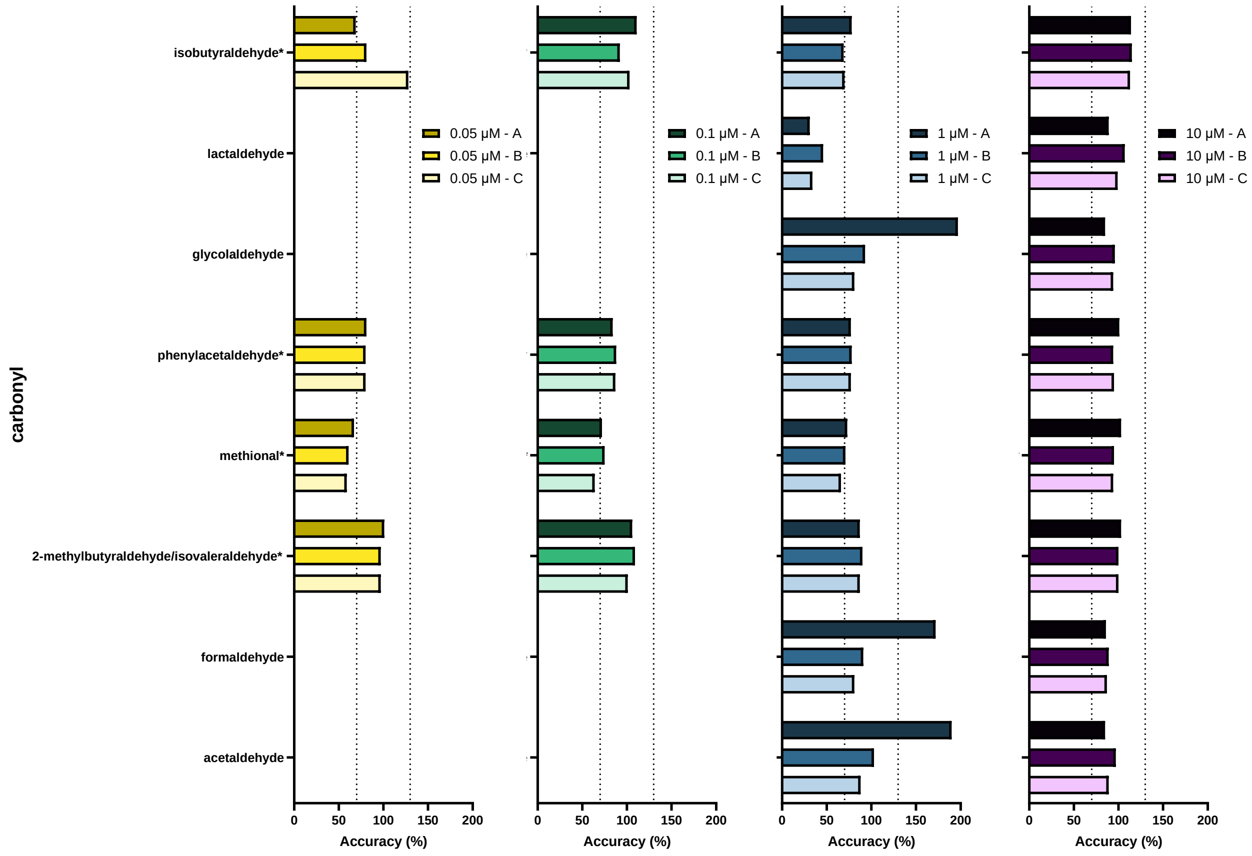

Figure S1.18. Stability experiments of triplicate spiked controls (0.05, 0.1, 1, and 10 μM) injected during the beginning, middle, and end of a 72-hour LC-HRMS analysis (indicated in the legend by A, B, and C, respectively) for the amino acid-related carbonyls.

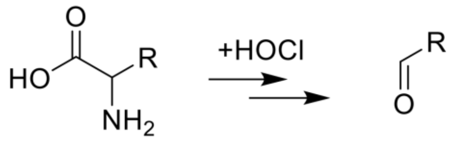

Figure S1.19. Abbreviated mechanism of amino acid transformation to aldehydes during chlorination.

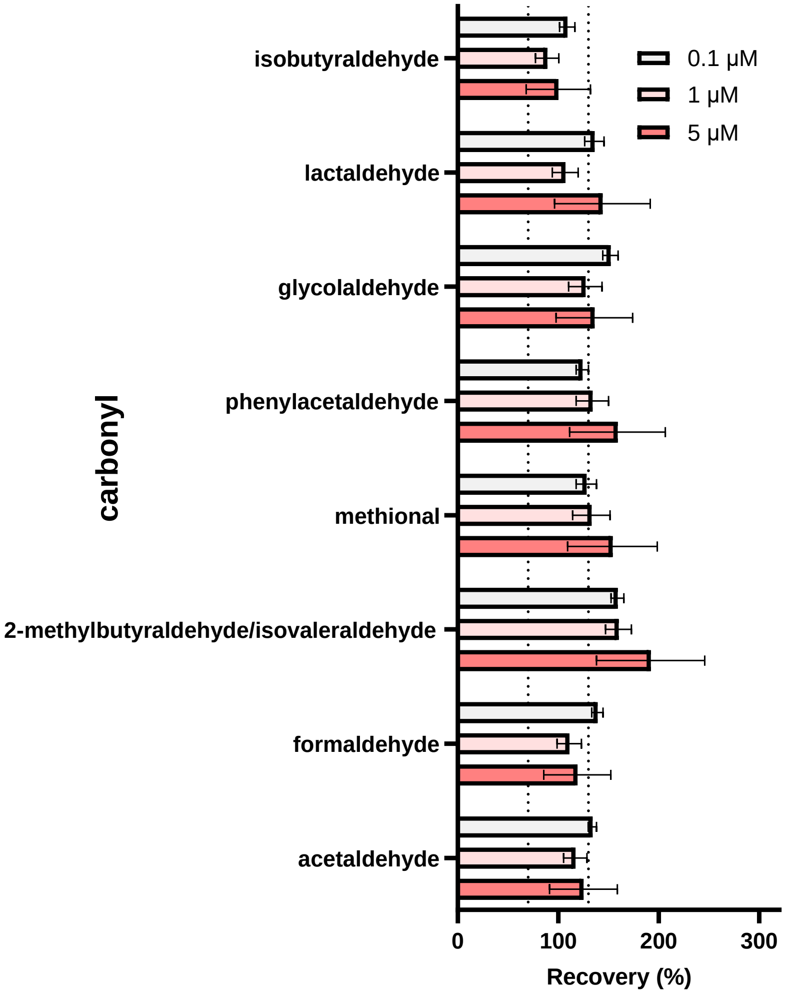

Figure S1.20. Recoveries (%) of amino acid carbonyl adducts after undergoing the SPREx extraction protocol (using 0.1, 1, or 5 μM of each carbonyl). Dashed lines across the x-axis indicate the carbonyl adducts that fall within the acceptable range of 70-130% recovery. Values are reported as the average value of each extracted sample peak area divided by the average peak area of the non-extracted samples. Error bars indicate the standard deviation (n = 3).

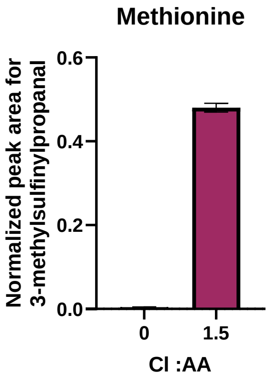

Figure S1.21. Peak area for 3-methylsulfinylpropanal (normalized to the benzaldehyde-d5 internal standard) versus chlorine dose for the methionine amino acid system (n = 3). The initial concentration for methionine was 10 µM. Error bars indicate the standard deviations.

References

(1) Zhang, Z.; Prasse, C. Chlorination of Para-Substituted Phenols: Formation of α, β-Unsaturated C4-Dialdehydes and C4-Dicarboxylic Acids. *Journal of Environmental Sciences* **2022**, S1001074222002017. https://doi.org/10.1016/j.jes.2022.04.029.

(2) Fantoni, N. Z.; El-Sagheer, A. H.; Brown, T. A Hitchhiker's Guide to Click-Chemistry with Nucleic Acids. *Chem. Rev.* **2021**, *121* (12), 7122–7154. https://doi.org/10.1021/acs.chemrev.0c00928.

(3) Wang, Q.; Chan, T. R.; Hilgraf, R.; Fokin, V. V.; Sharpless, K. B.; Finn, M. G. Bioconjugation by Copper(I)-Catalyzed Azide-Alkyne [3 + 2] Cycloaddition. *J. Am. Chem. Soc.* **2003**, *125* (11), 3192–3193. https://doi.org/10.1021/ja021381e.

(4) Hein, J. E.; Fokin, V. V. Copper-Catalyzed Azide–Alkyne Cycloaddition (CuAAC) and beyond: New Reactivity of Copper(i) Acetylides. *Chem. Soc. Rev.* **2010**, *39* (4), 1302. https://doi.org/10.1039/b904091a.

(5) Haldón, E.; Nicasio, M. C.; Pérez, P. J. Copper-Catalysed Azide–Alkyne Cycloadditions (CuAAC): An Update. *Org. Biomol. Chem.* **2015**, *13* (37), 9528–9550. https://doi.org/10.1039/C5OB01457C.

(6) Hong, V.; Presolski, S. I.; Ma, C.; Finn, M. G. Analysis and Optimization of Copper-Catalyzed Azide-Alkyne Cycloaddition for Bioconjugation. *Angewandte Chemie* **2009**, *121* (52), 10063–10067. https://doi.org/10.1002/ange.200905087.

(7) Presolski, S. I.; Hong, V. P.; Finn, M. G. Copper-Catalyzed Azide–Alkyne Click Chemistry for Bioconjugation. *Current Protocols in Chemical Biology* **2011**, *3* (4), 153–162. https://doi.org/10.1002/9780470559277.ch110148.

(8) Cooper, C.; Packer, N.; Williams, K. *Amino Acid Analysis Protocols*; Humana Press: New Jersey, 2000; Vol. 159. https://doi.org/10.1385/1592590470.

(9) *Basic Protein and Peptide Protocols*; Walker, J. M., Ed.; Methods in molecular biology; Humana Press: Totowa, N.J, 1994.

(10) Olson, B. J. S. C. Assays for Determination of Protein Concentration. *CP Pharmacology* **2016**, *73* (1). https://doi.org/10.1002/cpph.3.

(11) Sun, Y.; Kunc, F.; Balhara, V.; Coleman, B.; Kodra, O.; Raza, M.; Chen, M.; Brinkmann, A.; Lopinski, G. P.; Johnston, L. J. Quantification of Amine Functional Groups on Silica Nanoparticles: A Multi-Method Approach. *Nanoscale Adv.* **2019**, *1* (4), 1598–1607. https://doi.org/10.1039/C9NA00016J.

(12) Jue, R.; Lambert, J. M.; Pierce, L. R.; Traut, R. R. Addition of Sulfhydryl Groups of Escherichia Coli Ribosomes by Protein Modification with 2-Iminothiolane (Methyl 4-Mercaptobutyrimidate). *Biochemistry* **1978**, *17* (25), 5399–5406. https://doi.org/10.1021/bi00618a013.

(13) Becher, G.; Ovrum, N. M.; Christman, R. F. Novel Chlorination By-Products of Aquatic Humic Substances. *Science of The Total Environment* **1992**, *117–118*, 509–520. https://doi.org/10.1016/0048-9697(92)90115-9.

(14) Manasfi, T.; Houska, J.; Gebhardt, I.; von Gunten, U. Formation of Carbonyl Compounds during Ozonation of Lake Water and Wastewater: Development of a Non-Target Screening Method and Quantification of Target Compounds. *Water Research* **2023**, 119751. https://doi.org/10.1016/j.watres.2023.119751.

(15) Houska, J.; Manasfi, T.; Gebhardt, I.; von Gunten, U. Ozonation of Lake Water and Wastewater: Identification of Carbonous and Nitrogenous Carbonyl- Containing Oxidation

Byproducts by Non-Target Screening. *Water Research* **2022**, 119484. https://doi.org/10.1016/j.watres.2022.119484.
(16) Marron, E. L.; Prasse, C.; Buren, J. V.; Sedlak, D. L. Formation and Fate of Carbonyls in Potable Water Reuse Systems. *Environ. Sci. Technol.* **2020**, *54* (17), 10895–10903. https://doi.org/10.1021/acs.est.0c02793.
(17) Glaze, W. H.; Koga, M.; Cancilla, D. Ozonation Byproducts. 2. Improvement of an Aqueous-Phase Derivatization Method for the Detection of Formaldehyde and Other Carbonyl Compounds Formed by the Ozonation of Drinking Water. *Environ. Sci. Technol.* **1989**, *23* (7), 838–847. https://doi.org/10.1021/es00065a013.
(18) Froese, K. L.; Wolanski, A.; Hrudey, S. E. Factors Governing Odorous Aldehyde Formation as Disinfection By-Products in Drinking Water. *Water Research* **1999**, *33* (6), 1355–1364. https://doi.org/10.1016/S0043-1354(98)00357-1.
(19) How, Z. T.; Linge, K. L.; Busetti, F.; Joll, C. A. Chlorination of Amino Acids: Reaction Pathways and Reaction Rates. *Environ. Sci. Technol.* **2017**, *51* (9), 4870–4876. https://doi.org/10.1021/acs.est.6b04440.
(20) Prasse, C.; Wagner, M.; Schulz, R.; Ternes, T. A. Biotransformation of the Antiviral Drugs Acyclovir and Penciclovir in Activated Sludge Treatment. *Environ. Sci. Technol.* **2011**, *45* (7), 2761–2769. https://doi.org/10.1021/es103732y.
(21) Yeung, K.; Moore, N.; Sun, J.; Taylor-Edmonds, L.; Andrews, S.; Hofmann, R.; Peng, H. Thiol Reactome: A Nontargeted Strategy to Precisely Identify Thiol Reactive Drinking Water Disinfection Byproducts. *Environ. Sci. Technol.* **2023**, acs.est.2c05486. https://doi.org/10.1021/acs.est.2c05486.
(22) Böhme, A.; Thaens, D.; Paschke, A.; Schüürmann, G. Kinetic Glutathione Chemoassay To Quantify Thiol Reactivity of Organic Electrophiles—Application to α,β-Unsaturated Ketones, Acrylates, and Propiolates. *Chem. Res. Toxicol.* **2009**, *22* (4), 742–750. https://doi.org/10.1021/tx800492x.
(23) LoPachin, R. M.; Geohagen, B. C.; Nordstroem, L. U. Mechanisms of Soft and Hard Electrophile Toxicities. *Toxicology* **2019**, *418*, 62–69. https://doi.org/10.1016/j.tox.2019.02.005.
(24) LoPachin, R. M.; Gavin, T. Molecular Mechanisms of Aldehyde Toxicity: A Chemical Perspective. *Chem. Res. Toxicol.* **2014**, *27* (7), 1081–1091. https://doi.org/10.1021/tx5001046.
(25) Kalia, J.; Raines, R. T. Hydrolytic Stability of Hydrazones and Oximes. *Angew. Chem. Int. Ed.* **2008**, *47* (39), 7523–7526. https://doi.org/10.1002/anie.200802651.
(26) Sun, Y.; Tang, H.; Wang, Y. Progress and Challenges in Quantifying Carbonyl-Metabolomic Phenomes with LC-MS/MS. *Molecules* **2021**, *26* (20), 6147. https://doi.org/10.3390/molecules26206147.
(27) Abdel-Magid, A. F.; Carson, K. G.; Harris, B. D.; Maryanoff, C. A.; Shah, R. D. Reductive Amination of Aldehydes and Ketones with Sodium Triacetoxyborohydride. Studies on Direct and Indirect Reductive Amination Procedures [1]. *J. Org. Chem.* **1996**, *61* (11), 3849–3862. https://doi.org/10.1021/jo960057x.
(28) Kostal, J. Computational Chemistry in Predictive Toxicology. In *Advances in Molecular Toxicology*; Elsevier, 2016; Vol. 10, pp 139–186. https://doi.org/10.1016/B978-0-12-804700-2.00004-0.
