# Supplementary material for: Solid-Phase Reactivity-Directed Extraction (SPREx): An Alternative Approach for Simultaneous Extraction, Identification, and Prioritization of Toxic Electrophiles Produced in Water Treatment Applications
Source: ACS Environ Au. 2024 Sep 20;4(6):317–32. doi: 10.1021/acsenvironau.4c00025 (PMC11583095; doi:10.1021/acsenvironau.4c00025)
Supplement: Supplementary file 2 — vg4c00025_si_002.pdf [file vg4c00025_si_002.pdf]

Supporting Information 2

Solid-phase reactivity-directed extraction (SPREx): an alternative approach for simultaneous extraction, identification, and prioritization of toxic electrophiles produced in water treatment applications

Daisy N. Grace,[1] Matthew N. Newmeyer,[1] Carsten Prasse[1,2,*]

[1]Department of Environmental Health and Engineering, Johns Hopkins University, Baltimore, MD 21218, USA.

[2]Risk Sciences and Policy Institute, Bloomberg School of Public Health, Johns Hopkins University, Baltimore, MD 21205, USA

*Corresponding author. Email: cprasse1@jhu.edu

Contents

Part 1: Aminooxy (ONH_2) bead system 6

Part 2: Hydrazide (NNH_2) bead system 255

Part 3: Amine (NH_2) bead system 366

Part 4: Reduced amine (NH_2 + $NaBH_3CN$) bead system 377

Part 1: Aminooxy (ONH_2) bead system

Figure S2.1. Signature fragment pattern from the ONH_2 bead system. S6

Figure S2.2. Selected ion chromatogram for the 1,4-benzoquinone Schiff base adduct via ONH_2-bead derivatization (*m/z* 437). S6

Figure S2.3. Selected ion chromatogram and MS^2 spectrum for the 2-butene-1,4-dial Schiff base adduct via ONH_2-bead derivatization (earlier retention time; *m/z* 413). S7

Figure S2.4. Selected ion chromatogram and MS^2 spectrum for the 2-butene-1,4-dial Schiff base adduct via ONH_2-bead derivatization (later retention time; *m/z* 413). S7

Figure S2.5. Selected ion chromatogram and MS^2 spectrum for the 2-butene-1,4-dial Michael addition adduct via ONH_2-bead derivatization (*m/z* 431). S8

Figure S2.6. Selected ion chromatogram and MS^2 spectrum for the 2-methylbutyraldehyde Schiff base adduct via ONH_2-bead derivatization (*m/z* 415). S8

Figure S2.7. Selected ion chromatogram and MS^2 spectrum for the 3-methylcrotonaldehyde Schiff base adduct via ONH_2-bead derivatization (*m/z* 413). S9

Figure S2.8. Selected ion chromatogram and MS^2 spectrum for the 3-methylcrotonaldehyde Michael addition adduct via ONH_2-bead derivatization (*m/z* 431). S9

Figure S2.9. Selected ion chromatogram and MS^2 spectrum for the acetaldehyde Schiff base adduct via ONH_2-bead derivatization (*m/z* 373). S10

Figure S2.10. Selected ion chromatogram and MS^2 spectrum for the acrolein Schiff base adduct via ONH_2-bead derivatization (*m/z* 385). S10

Figure S2.11. Selected ion chromatogram and MS^2 spectrum for the acrolein Michael addition adduct via ONH_2-bead derivatization (*m/z* 403). S11

Figure S2.12. Selected ion chromatogram for the acrolein dimer Schiff base adduct via ONH_2-bead derivatization (*m/z* 423). S11

Figure S2.13. Selected ion chromatogram for the acrylamide Schiff base adduct via ONH_2-bead derivatization (*m/z* 400). S12

Figure S2.14. Selected ion chromatogram and MS^2 spectrum for the benzaldehyde Schiff base adduct via ONH_2-bead derivatization (*m/z* 435). S12

Figure S2.15. Selected ion chromatogram and MS^2 spectrum for the benzaldehyde-d5 Schiff base adduct via ONH_2-bead derivatization (*m/z* 440). S13

Figure S2.16. Selected ion chromatogram and MS^2 spectrum for the butanal Schiff base adduct via ONH_2-bead derivatization (*m/z* 401). S13

Figure S2.17. Selected ion chromatogram and MS^2 spectrum for the butanal-d2 Schiff base adduct via ONH_2-bead derivatization (*m/z* 403). S14

Figure S2.18. Selected ion chromatogram and MS^2 spectrum for the crotonaldehyde/2-methacrolein/methyl vinyl ketone (isomers) Schiff base adduct via ONH_2-bead derivatization (*m/z* 399). S14
Figure S2.19. Selected ion chromatogram and MS^2 spectrum for the crotonaldehyde/2-methacrolein/methyl vinyl ketone (isomers) Michael addition adduct via ONH_2-bead derivatization (earlier retention time; *m/z* 417). S15
Figure S2.20. Selected ion chromatogram and MS^2 spectrum for the crotonaldehyde/2-methacrolein/methyl vinyl ketone (isomers) Michael addition adduct via ONH_2-bead derivatization (later retention time; *m/z* 417). S15
Figure S2.21. Selected ion chromatogram and MS^2 spectrum for the formaldehyde Schiff base adduct via ONH_2-bead derivatization (*m/z* 359). S16
Figure S2.22. Selected ion chromatogram and MS^2 spectrum for the furaldehyde Schiff base adduct via ONH_2-bead derivatization (*m/z* 425). S16
Figure S2.23. Selected ion chromatogram and MS^2 spectrum for the glycolaldehyde Schiff base adduct via ONH_2-bead derivatization (*m/z* 389). S17
Figure S2.24. Selected ion chromatogram and MS^2 spectrum for the glyoxal Schiff base adduct via ONH_2-bead derivatization (*m/z* 387). S17
Figure S2.25. Selected ion chromatogram and MS^2 spectrum for the glyoxal hydrate Schiff base adduct via ONH_2-bead derivatization (*m/z* 405). S18
Figure S2.26. Selected ion chromatogram and MS^2 spectrum for the glyoxal dimer Schiff base adduct via ONH_2-bead derivatization (*m/z* 463). S18
Figure S2.27. Selected ion chromatogram and MS^2 spectrum for the hexanal Schiff base adduct via ONH_2-bead derivatization (*m/z* 429). S19
Figure S2.28. Selected ion chromatogram and MS^2 spectrum for the isobutyraldehyde Schiff base adduct via ONH_2-bead derivatization (*m/z* 401). S19
Figure S2.29. Selected ion chromatogram and MS^2 spectrum for the isovaleraldehyde Schiff base adduct via ONH_2-bead derivatization (*m/z* 415). S20
Figure S2.30. Selected ion chromatogram and MS^2 spectrum for the lactaldehyde Schiff base adduct via ONH_2-bead derivatization (*m/z* 403). S20
Figure S2.31. Selected ion chromatogram and MS^2 spectrum for the methacrylate Schiff base adduct via ONH_2-bead derivatization (*m/z* 429). S21
Figure S2.32. Selected ion chromatogram for the methacrylate Michael addition adduct via ONH_2-bead derivatization (*m/z* 447). S21
Figure S2.33. Selected ion chromatogram and MS^2 spectrum for the methional Schiff base adduct via ONH_2-bead derivatization (*m/z* 433). S22
Figure S2.34. Selected ion chromatogram and MS^2 spectrum for the 3-methylsulfinylpropanal Schiff base adduct via ONH_2-bead derivatization (*m/z* 449). S22
Figure S2.35. Selected ion chromatogram and MS^2 spectrum for the methylglyoxal Schiff base adduct via ONH_2-bead derivatization (*m/z* 401). S23

Figure S2.36. Selected ion chromatogram and MS^2 spectrum for the methylglyoxal hydrate Schiff base adduct via ONH_2-bead derivatization (*m/z* 419). S23
Figure S2.37. Selected ion chromatogram and MS^2 spectrum for the phenylacetaldehyde Schiff base adduct via ONH_2-bead derivatization (*m/z* 449). S24

Part 2: Hydrazide (NNH_2) bead system

Figure S2.38. Selected ion chromatogram for the 2-butene-1,4-dial Schiff base adduct via NNH_2-bead derivatization (*m/z* 427). S25
Figure S2.39. Selected ion chromatogram for the 2-butene-1,4-dial Michael addition adduct via NNH_2-bead derivatization (*m/z* 445). S25
Figure S2.40. Selected ion chromatogram and MS^2 spectrum for the 3-methylcrotonaldehyde Schiff base adduct via NNH_2-bead derivatization (*m/z* 427). S26
Figure S2.41. Selected ion chromatogram for the 3-methylcrotonaldehyde Michael addition adduct via NNH_2-bead derivatization (*m/z* 445). S26
Figure S2.42. Selected ion chromatogram and MS^2 spectrum for the acrolein Schiff base adduct via NNH_2-bead derivatization (*m/z* 399). S27
Figure S2.43. Selected ion chromatogram and MS^2 spectrum for the acrolein Michael addition adduct via NNH_2-bead derivatization (*m/z* 417). S27
Figure S2.44. Selected ion chromatogram for the benzaldehyde Schiff base adduct via NNH_2-bead derivatization (*m/z* 449). S28
Figure S2.45. Selected ion chromatogram and MS^2 spectrum for the benzaldehyde-d5 Schiff base adduct via NNH_2-bead derivatization (*m/z* 454). S28
Figure S2.46. Selected ion chromatogram and MS^2 spectrum for the butanal Schiff base adduct via NNH_2-bead derivatization (*m/z* 415). S29
Figure S2.47. Selected ion chromatogram and MS^2 spectrum for the butanal-d2 Schiff base adduct via NNH_2-bead derivatization (*m/z* 417). S29
Figure S2.48. Selected ion chromatogram and MS^2 spectrum for the crotonaldehyde/2-methacrolein/methyl vinyl ketone (isomers) Schiff base adduct via NNH_2-bead derivatization (*m/z* 413). S30
Figure S2.49. Selected ion chromatogram for the crotonaldehyde/2-methacrolein/methyl vinyl ketone (isomers) Michael addition adduct via NNH_2-bead derivatization (*m/z* 431). S30
Figure S2.50. Selected ion chromatogram for the crotonaldehyde dimer Schiff base adduct via NNH_2-bead derivatization (*m/z* 483). S31
Figure S2.51. Selected ion chromatogram and MS^2 spectrum for the furaldehyde Schiff base adduct via NNH_2-bead derivatization (*m/z* 439). S31
Figure S2.52. Selected ion chromatogram for the glyoxal Schiff base adduct via NNH_2-bead derivatization (*m/z* 401). S32

Figure S2.53. Selected ion chromatogram for the glyoxal hydrate Schiff base adduct via NNH_2-bead derivatization (*m/z* 419). S32
Figure S2.54. Selected ion chromatogram and MS^2 spectrum for the hexanal Schiff base adduct via NNH_2-bead derivatization (*m/z* 443). S33
Figure S2.55. Selected ion chromatogram for the methacrylate Schiff base adduct via NNH_2-bead derivatization (*m/z* 443). S33
Figure S2.56. Selected ion chromatogram for the methacrylate Michael addition adduct via NNH_2-bead derivatization (*m/z* 461). S34
Figure S2.57. Selected ion chromatogram and MS^2 spectrum for the methylglyoxal Schiff base adduct via NNH_2-bead derivatization (*m/z* 415). S34
Figure S2.58. Selected ion chromatogram for the methylglyoxal hydrate Schiff base adduct via NNH_2-bead derivatization (*m/z* 433). S35
Figure S2.59. Selected ion chromatogram for the methylglyoxal dimer Schiff base adduct via NNH_2-bead derivatization (*m/z* 505). S35

Part 3: Amine (NH_2) bead system

Figure S2.60. Selected ion chromatogram and MS^2 spectrum for the 2-butene-1,4-dial Schiff base adduct via NH_2-bead derivatization (*m/z* 340). S36
Figure S2.61. Selected ion chromatogram for the glyoxal Schiff base adduct via NH_2-bead derivatization (*m/z* 314). S36

Part 4: Reduced amine (NH_2 + $NaBH_3CN$) bead system

Figure S2.62. Selected ion chromatogram for the 1,4-benzoquinone Schiff base adduct via reduced NH_2-bead derivatization (*m/z* 366). S37
Figure S2.63. Selected ion chromatogram for the 2-butene-1,4-dial Schiff base adduct via reduced NH_2-bead derivatization (*m/z* 342). S37
Figure S2.64. Selected ion chromatogram for the butanal Schiff base adduct via reduced NH_2-bead derivatization (*m/z* 330). S38
Figure S2.65. Selected ion chromatogram for the butanal-d2 Schiff base adduct via reduced NH_2-bead derivatization (*m/z* 332). S38
Figure S2.66. Selected ion chromatogram and MS^2 spectrum for the glyoxal Schiff base adduct via reduced NH_2-bead derivatization (*m/z* 316). S39
Figure S2.67. Selected ion chromatogram for the hexanal Schiff base adduct via reduced NH_2-bead derivatization (*m/z* 358). S39
Figure S2.68. Selected ion chromatogram and MS^2 spectrum for the methylglyoxal Schiff base adduct via reduced NH_2-bead derivatization (*m/z* 330). S40

Part 1: Aminooxy (ONH_2) bead system

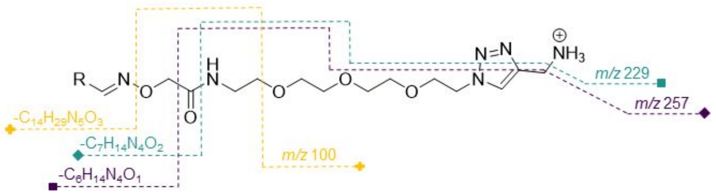

Figure S2.1. Signature fragment pattern from the ONH_2 bead system. Note the appearance of these fragments (i.e., *m/z* 229 & 257) in the following MS^2 spectra for the ONH_2-carbonyl adducts. The *m/z* 100 fragment was also present for many of the ONH_2-carbonyl adducts. Neutral losses of -$C_9H_{18}N_4O_3$, -N_3H_3, and -NH_3 were also observed in the majority of the ONH_2-carbonyl MS^2 spectra (see figures below).

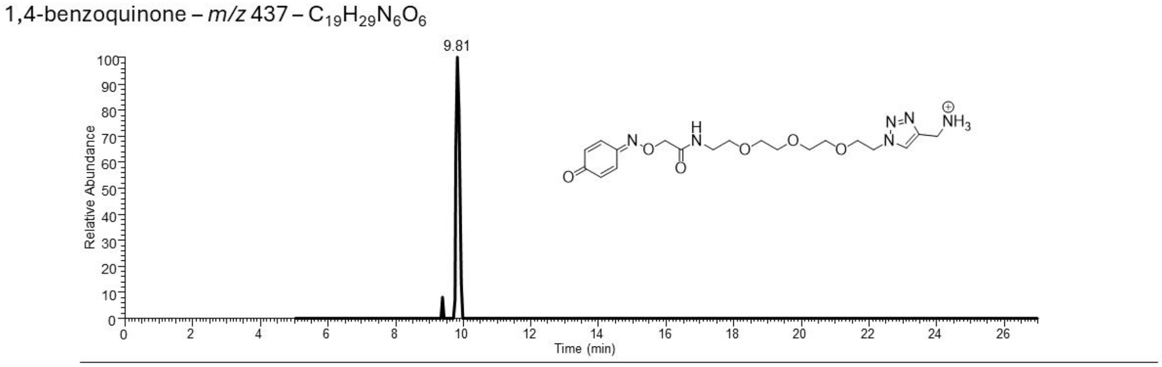

Figure S2.2. Selected ion chromatogram for the 1,4-benzoquinone Schiff base adduct via ONH_2-bead derivatization (*m/z* 437). MS^2 information was not available.

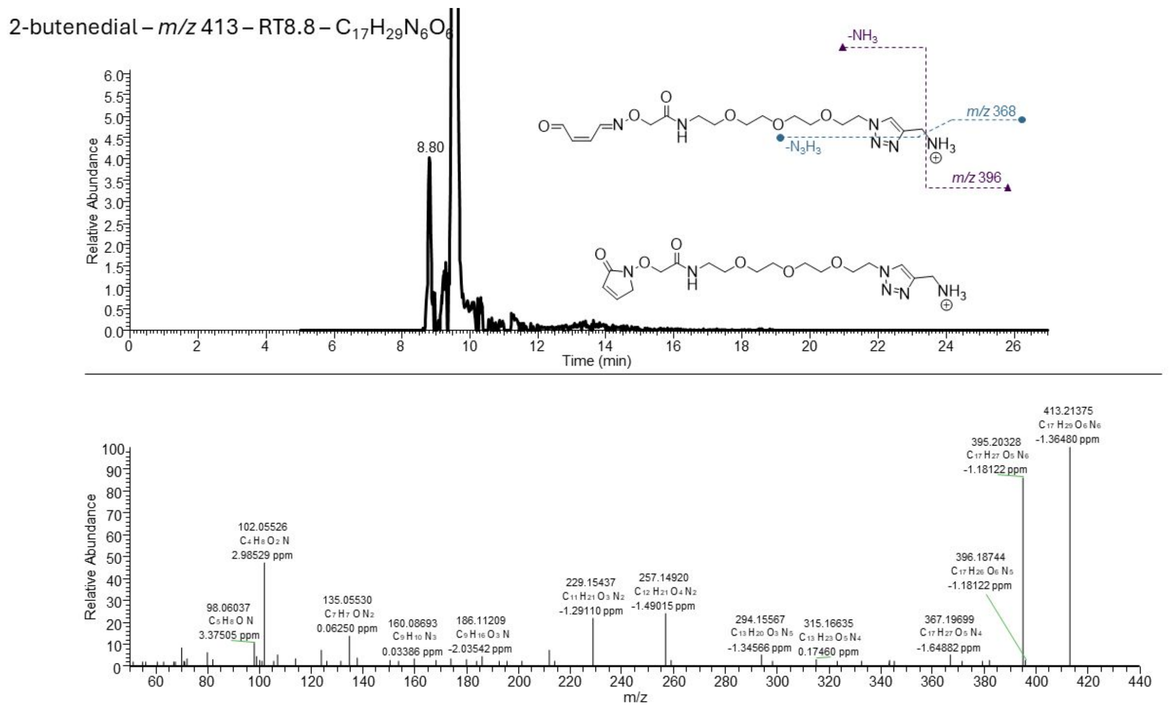

Figure S2.3. Selected ion chromatogram and MS^2 spectrum for the 2-butene-1,4-dial Schiff base adduct via ONH_2-bead derivatization (earlier retention time; *m/z* 413). Both the ring and open-chain proposed structures are depicted. RT = retention time.

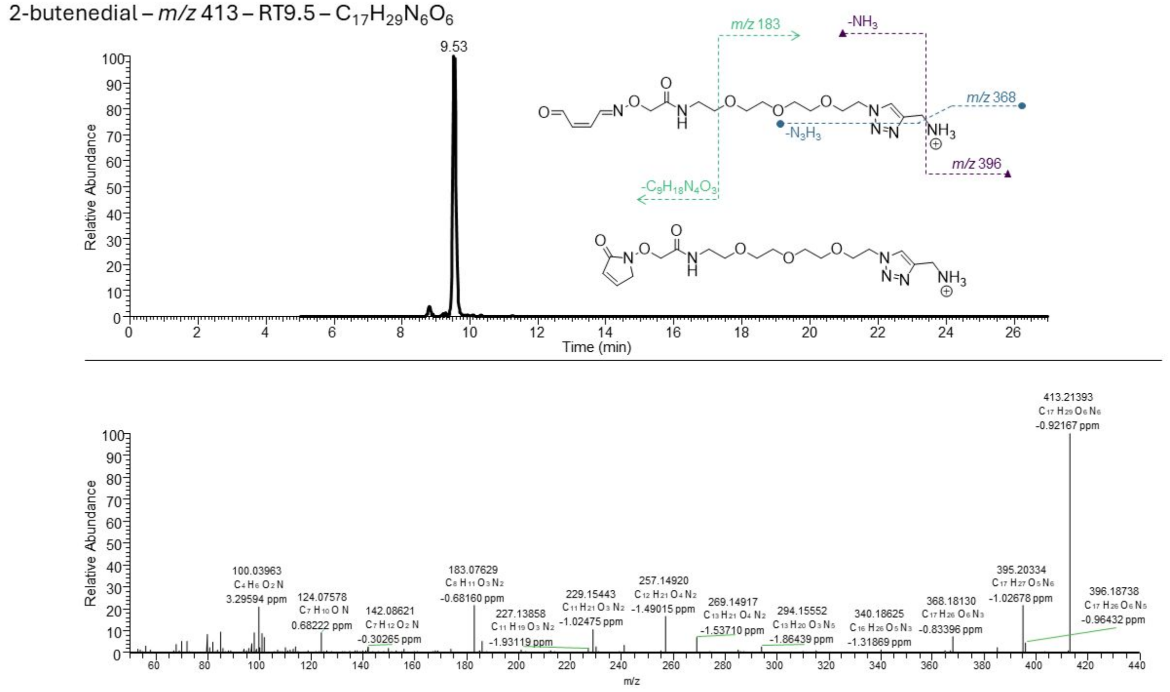

Figure S2.4. Selected ion chromatogram and MS^2 spectrum for the 2-butene-1,4-dial Schiff base adduct via ONH_2-bead derivatization (later retention time; *m/z* 413). Both the ring and open-chain proposed structures are depicted. RT = retention time.

2-butenedial – *m/z* 431 – $C_{17}H_{31}N_6O_7$

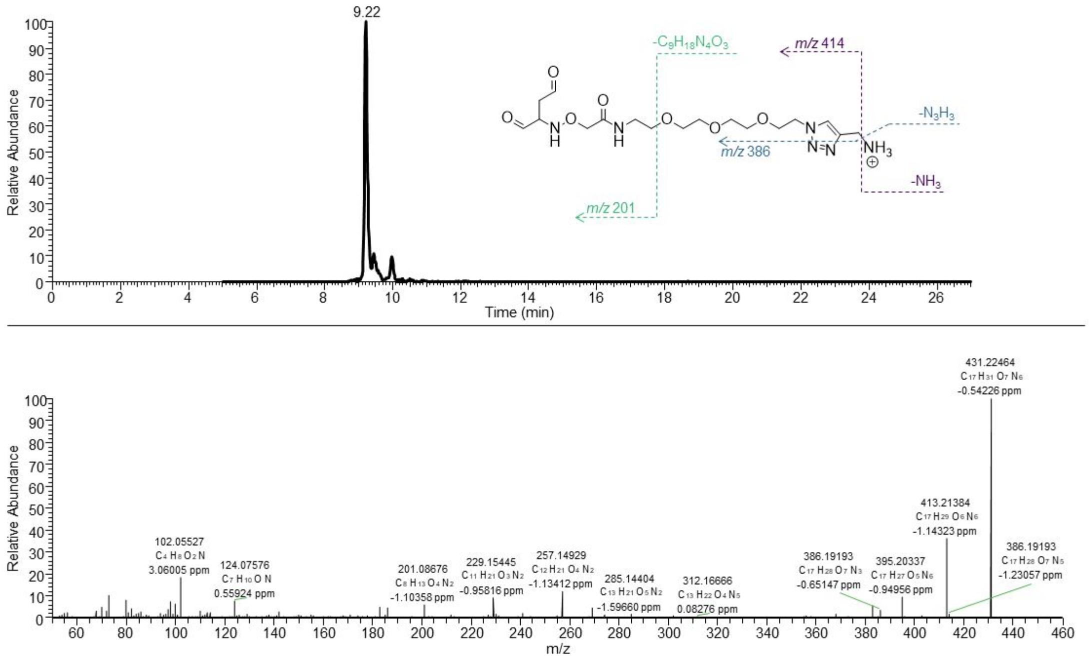

Figure S2.5. Selected ion chromatogram and MS[2] spectrum for the 2-butene-1,4-dial Michael addition adduct via ONH_2-bead derivatization (*m/z* 431).

2-methylbutyraldehyde – *m/z* 415 – $C_{18}H_{35}N_6O_5$

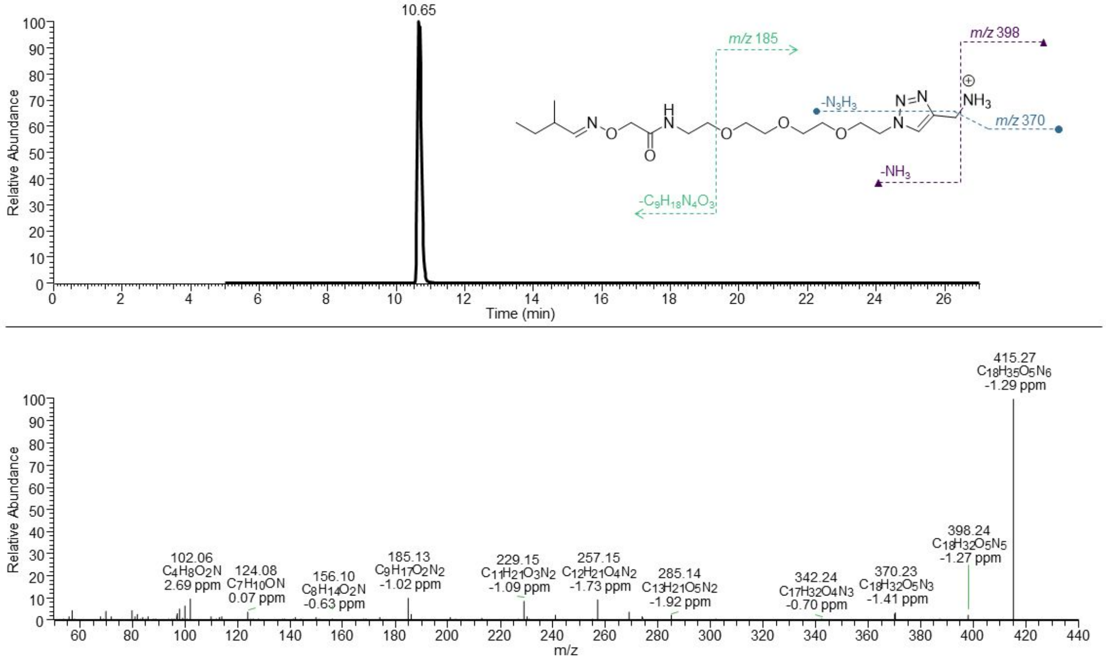

Figure S2.6. Selected ion chromatogram and MS[2] spectrum for the 2-methylbutyraldehyde Schiff base adduct via ONH_2-bead derivatization (*m/z* 415).

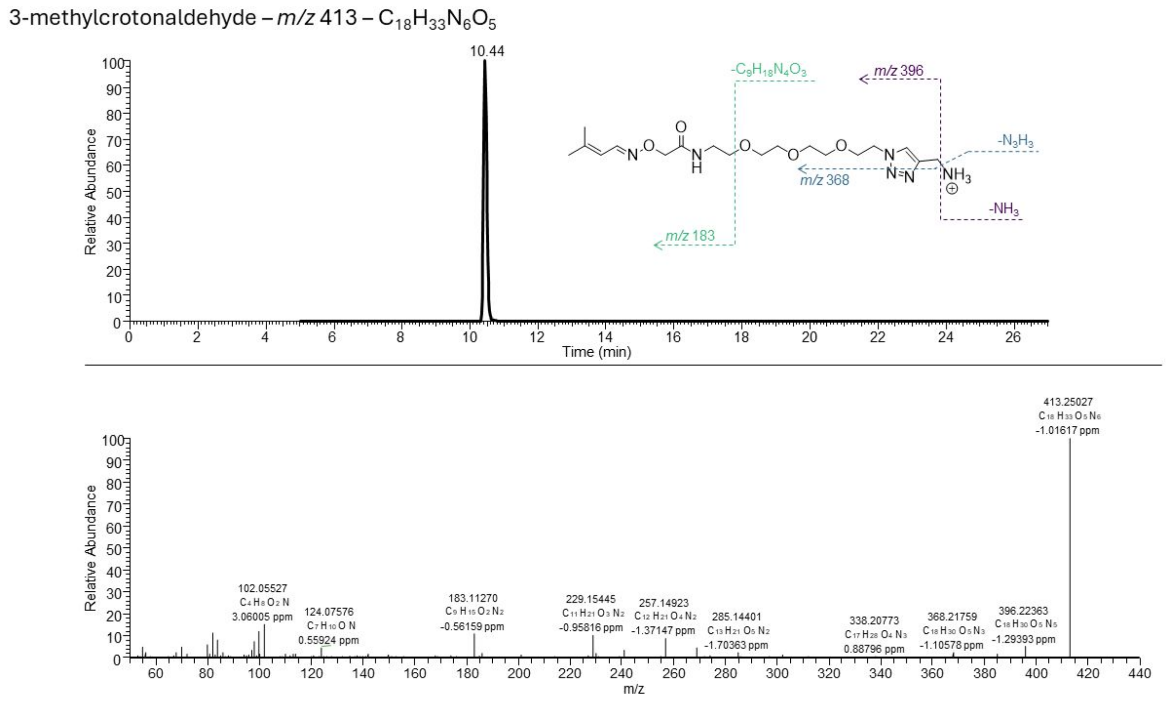

Figure S2.7. Selected ion chromatogram and MS^2 spectrum for the 3-methylcrotonaldehyde Schiff base adduct via ONH_2-bead derivatization (*m/z* 413).

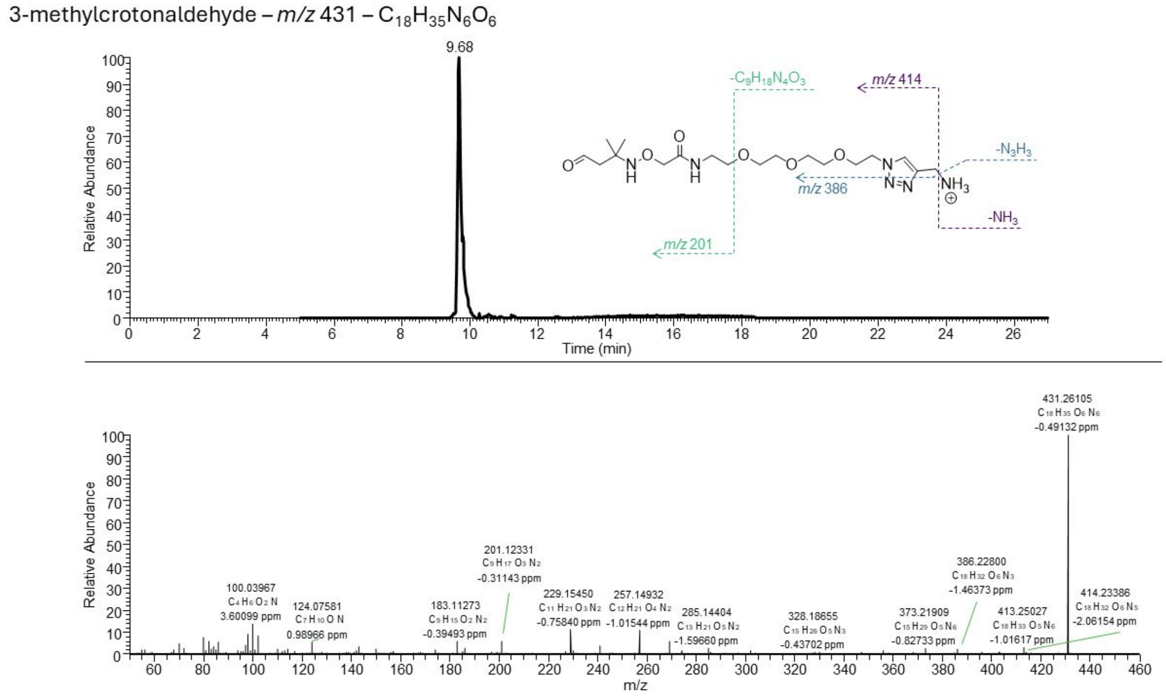

Figure S2.8. Selected ion chromatogram and MS^2 spectrum for the 3-methylcrotonaldehyde Michael addition adduct via ONH_2-bead derivatization (*m/z* 431).

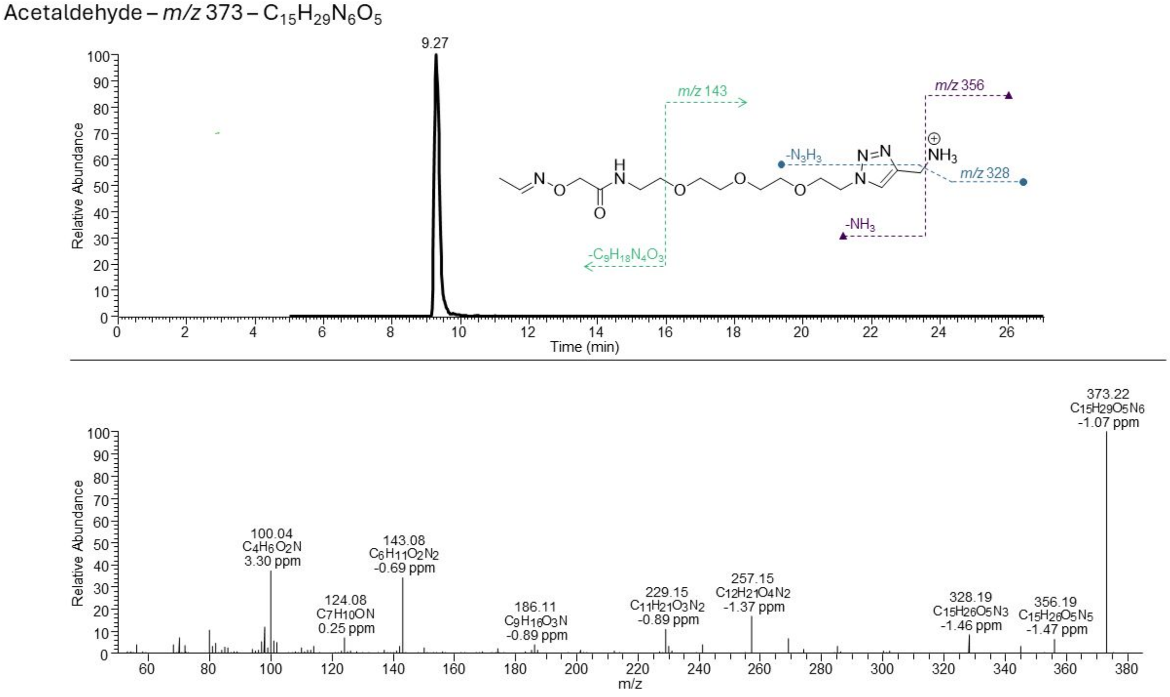

Figure S2.9. Selected ion chromatogram and MS^2 spectrum for the acetaldehyde Schiff base adduct via ONH$_2$-bead derivatization (*m/z* 373).

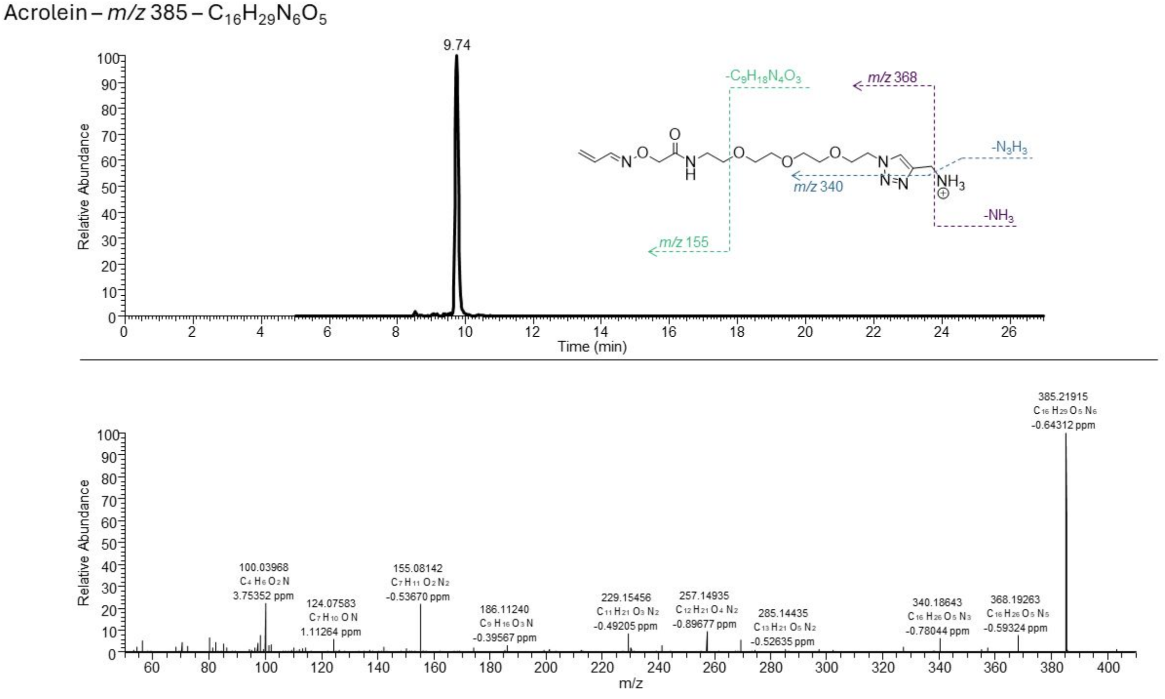

Figure S2.10. Selected ion chromatogram and MS^2 spectrum for the acrolein Schiff base adduct via ONH2-bead derivatization (*m/z* 385).

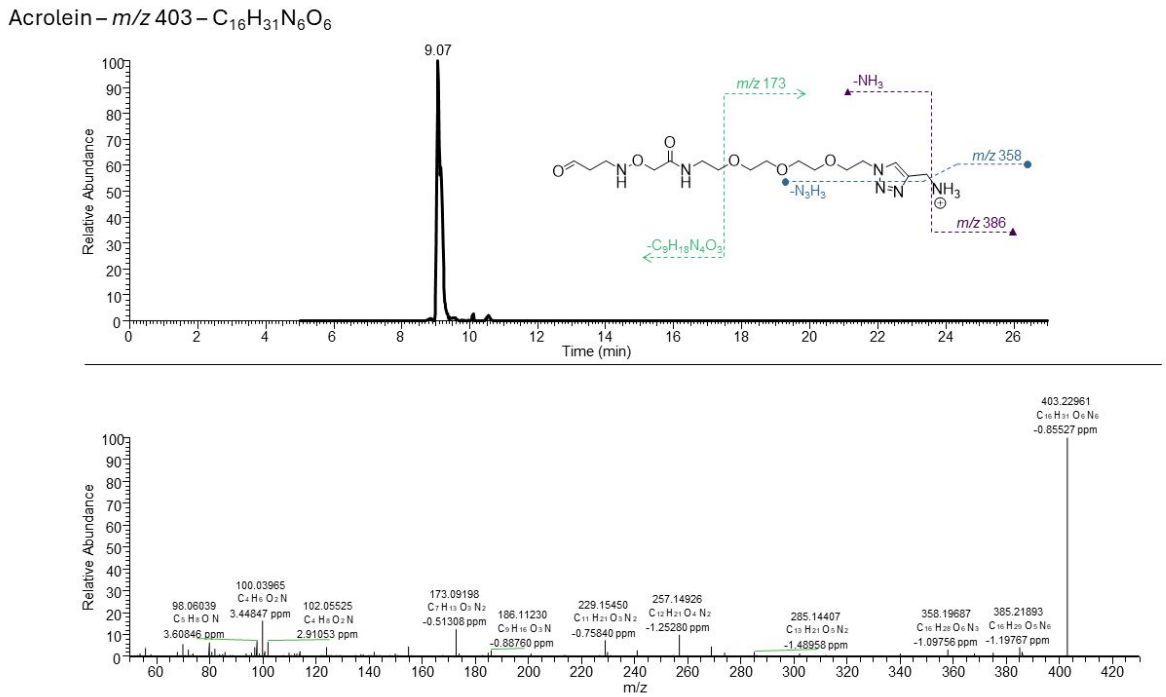

Figure S2.11. Selected ion chromatogram and MS^2 spectrum for the acrolein Michael addition adduct via ONH_2-bead derivatization (*m/z* 403).

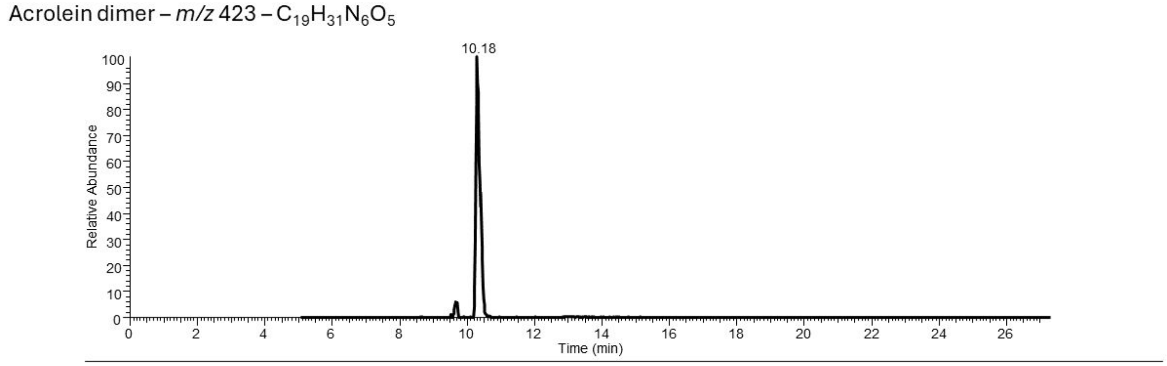

Figure S2.12. Selected ion chromatogram for the acrolein dimer Schiff base adduct via ONH_2-bead derivatization (*m/z* 423). MS^2 information was not available.

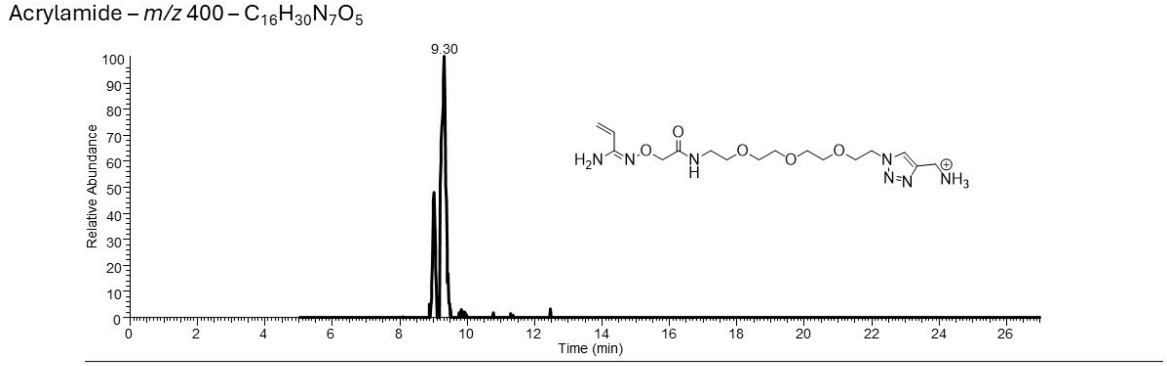

Figure S2.13. Selected ion chromatogram for the acrylamide Schiff base adduct via ONH_2-bead derivatization (*m/z* 400). MS^2 information was not available.

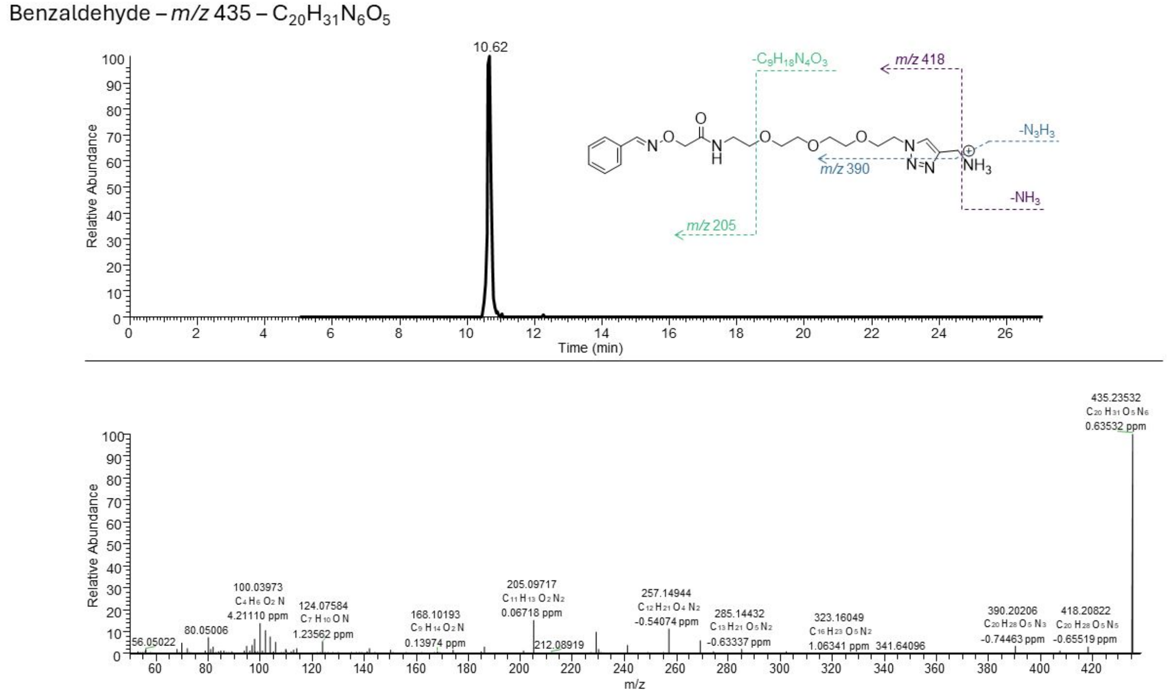

Figure S2.14. Selected ion chromatogram and MS^2 spectrum for the benzaldehyde Schiff base adduct via ONH_2-bead derivatization (*m/z* 435).

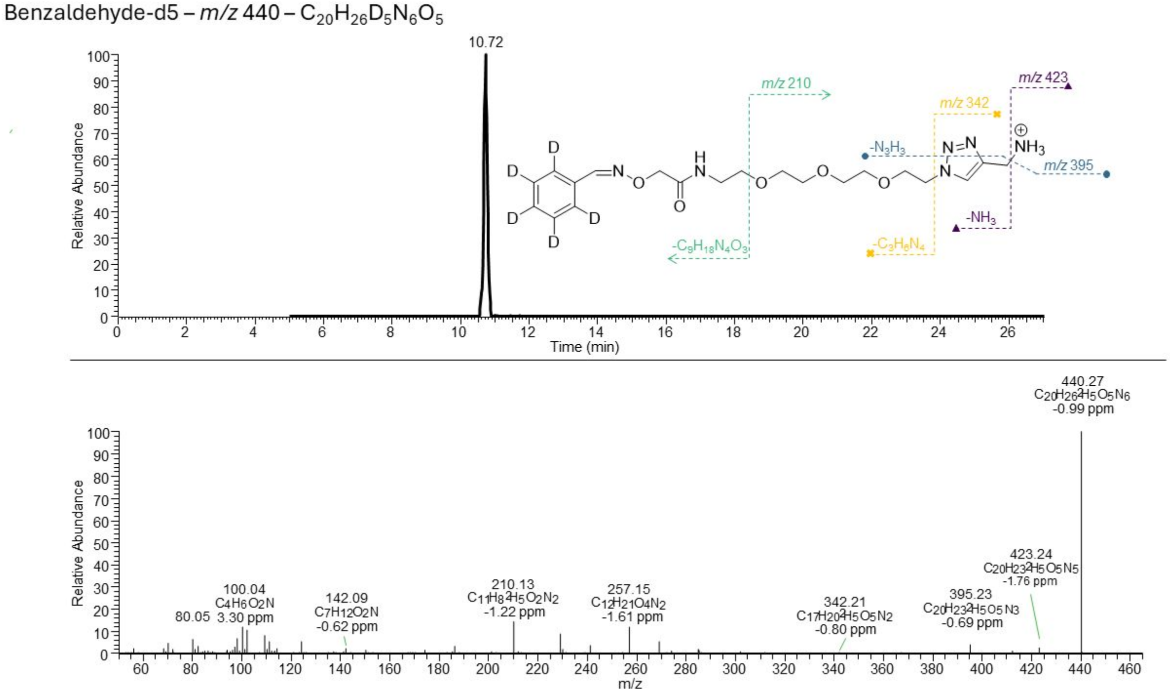

Figure S2.15. Selected ion chromatogram and MS^2 spectrum for the benzaldehyde-d5 Schiff base adduct via ONH$_2$-bead derivatization (*m/z* 440).

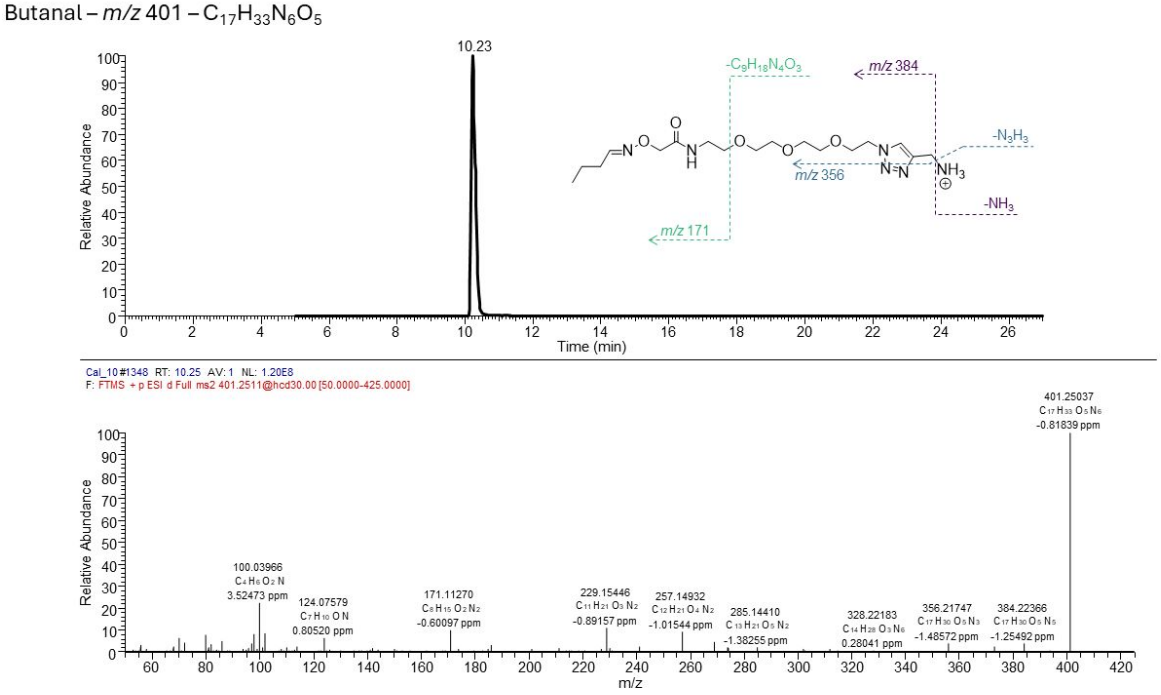

Figure S2.16. Selected ion chromatogram and MS^2 spectrum for the butanal Schiff base adduct via ONH$_2$-bead derivatization (*m/z* 401).

Butanal-d2 – *m/z* 403 – $C_{17}H_{31}D_2N_6O_5$

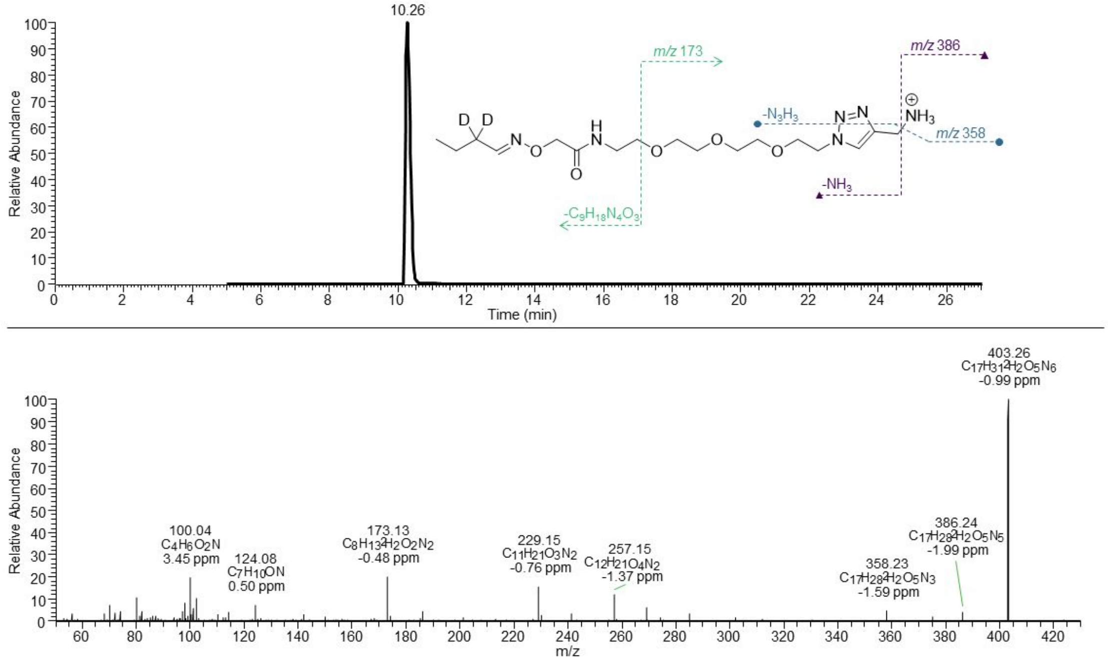

Figure S2.17. Selected ion chromatogram and MS^2 spectrum for the butanal-d2 Schiff base adduct via ONH_2-bead derivatization (*m/z* 403).

Crotonaldehyde, 2-methacrolein, methyl vinyl ketone (isomers) – *m/z* 399 – $C_{17}H_{31}N_6O_5$

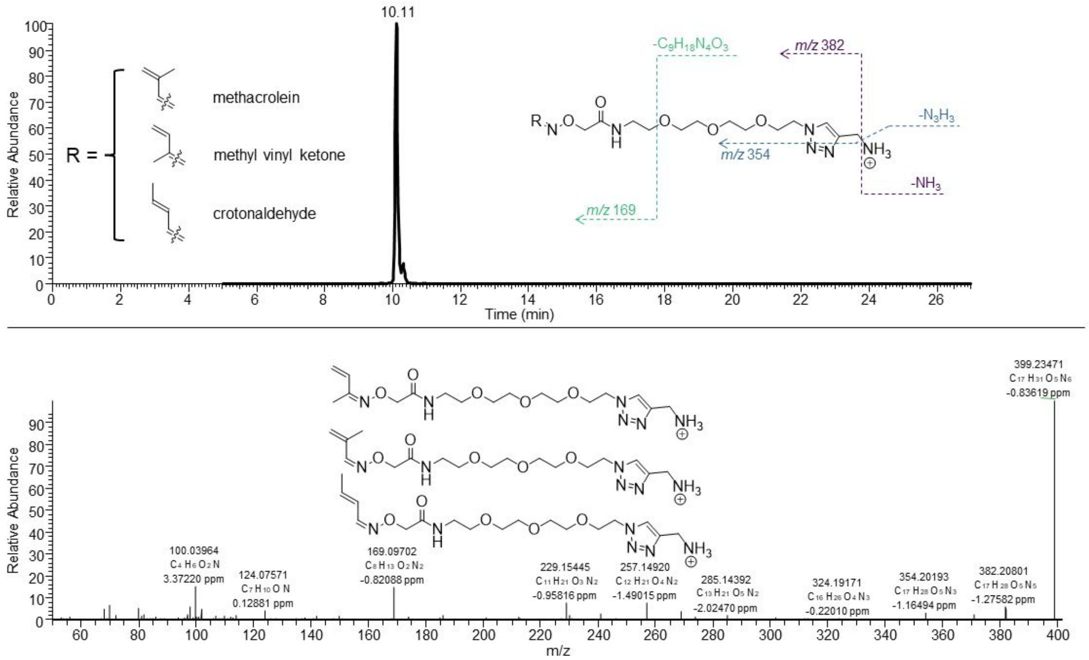

Figure S2.18. Selected ion chromatogram and MS^2 spectrum for the crotonaldehyde/2-methacrolein/methyl vinyl ketone (isomers) Schiff base adduct via ONH_2-bead derivatization (*m/z* 399).

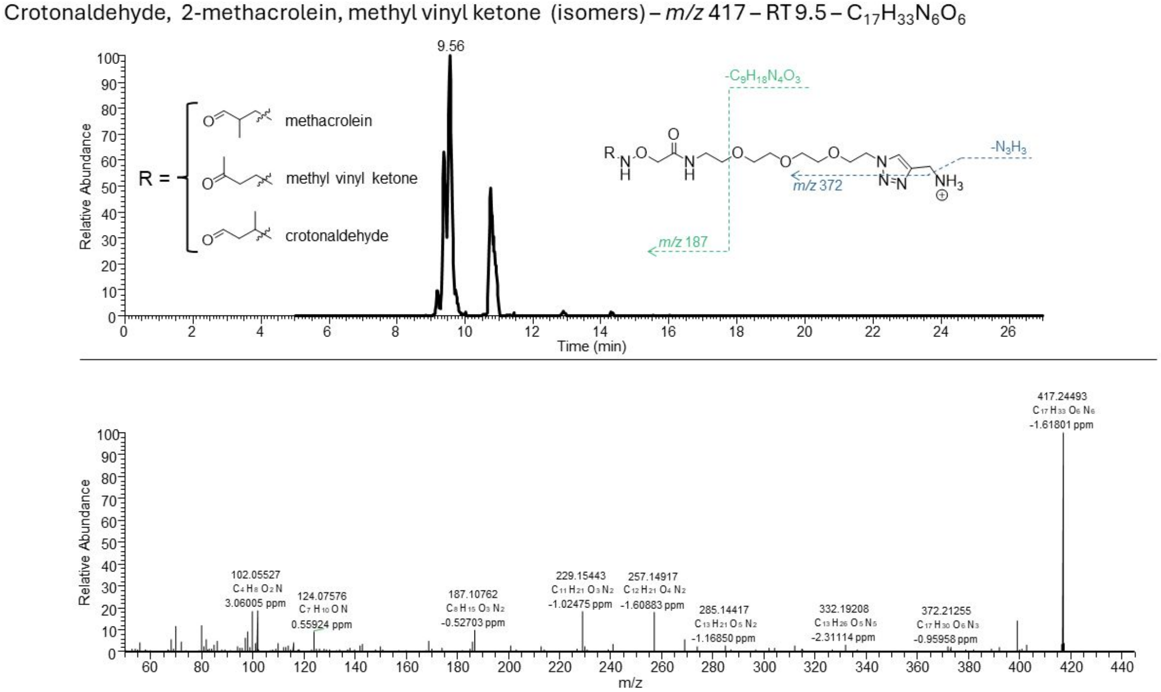

Figure S2.19. Selected ion chromatogram and MS^2 spectrum for the crotonaldehyde/2-methacrolein/methyl vinyl ketone (isomers) Michael addition adduct via ONH_2-bead derivatization (earlier retention time; *m/z* 417). RT = retention time.

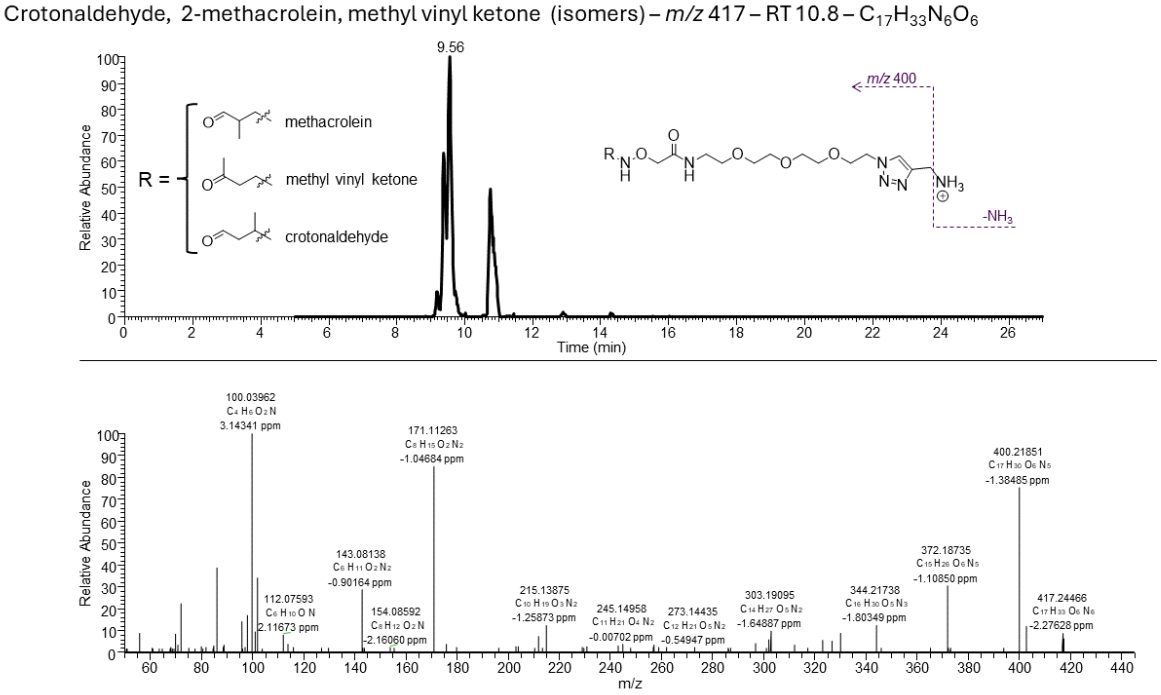

Figure S2.20. Selected ion chromatogram and MS^2 spectrum for the crotonaldehyde/2-methacrolein/methyl vinyl ketone (isomers) Michael addition adduct via ONH_2-bead derivatization (later retention time; *m/z* 417). RT = retention time.

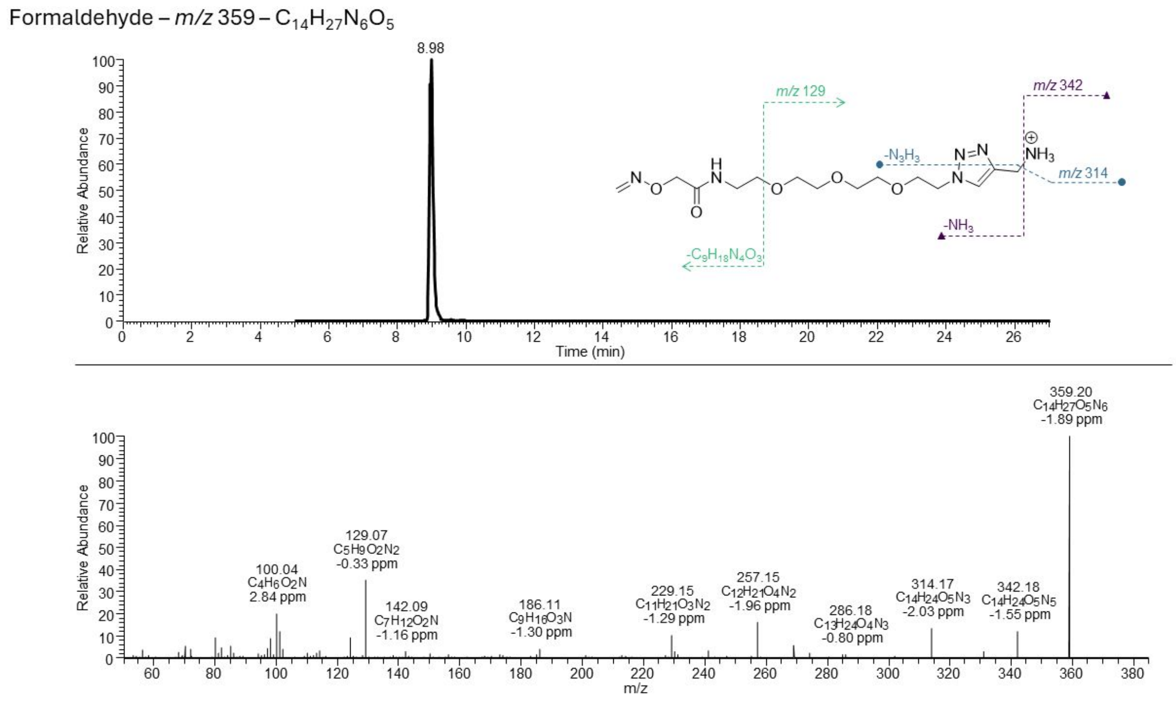

Figure S2.21. Selected ion chromatogram and MS^2 spectrum for the formaldehyde Schiff base adduct via ONH_2-bead derivatization (*m/z* 359).

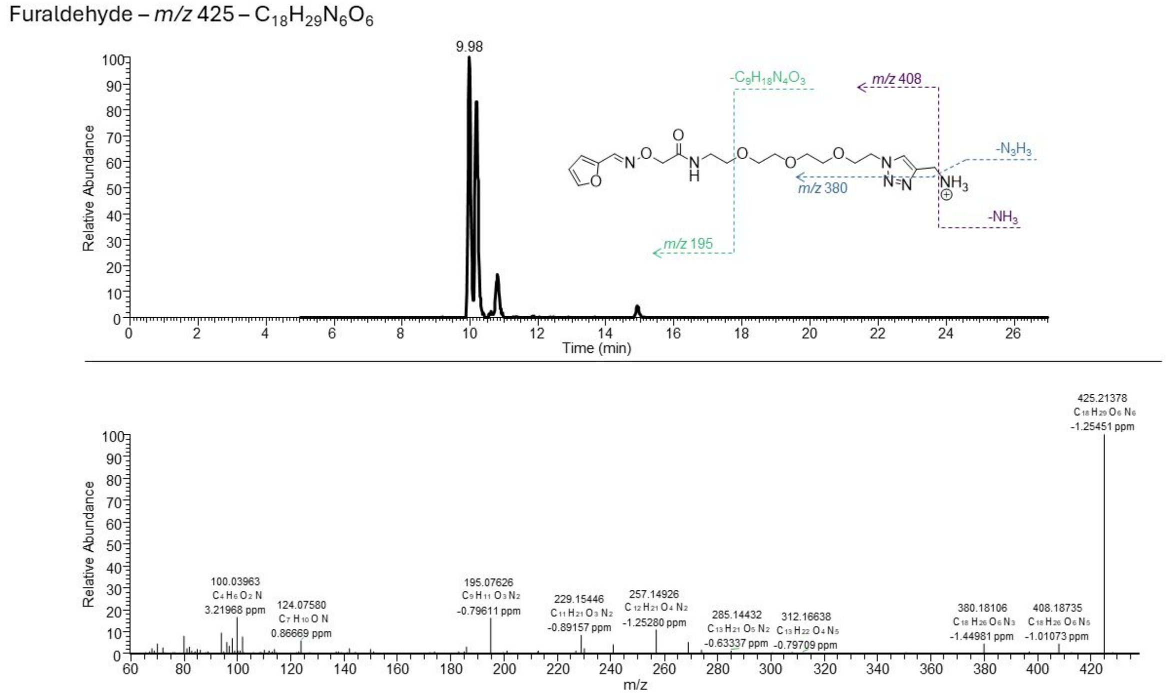

Figure S2.22. Selected ion chromatogram and MS^2 spectrum for the furaldehyde Schiff base adduct via ONH_2-bead derivatization (*m/z* 425).

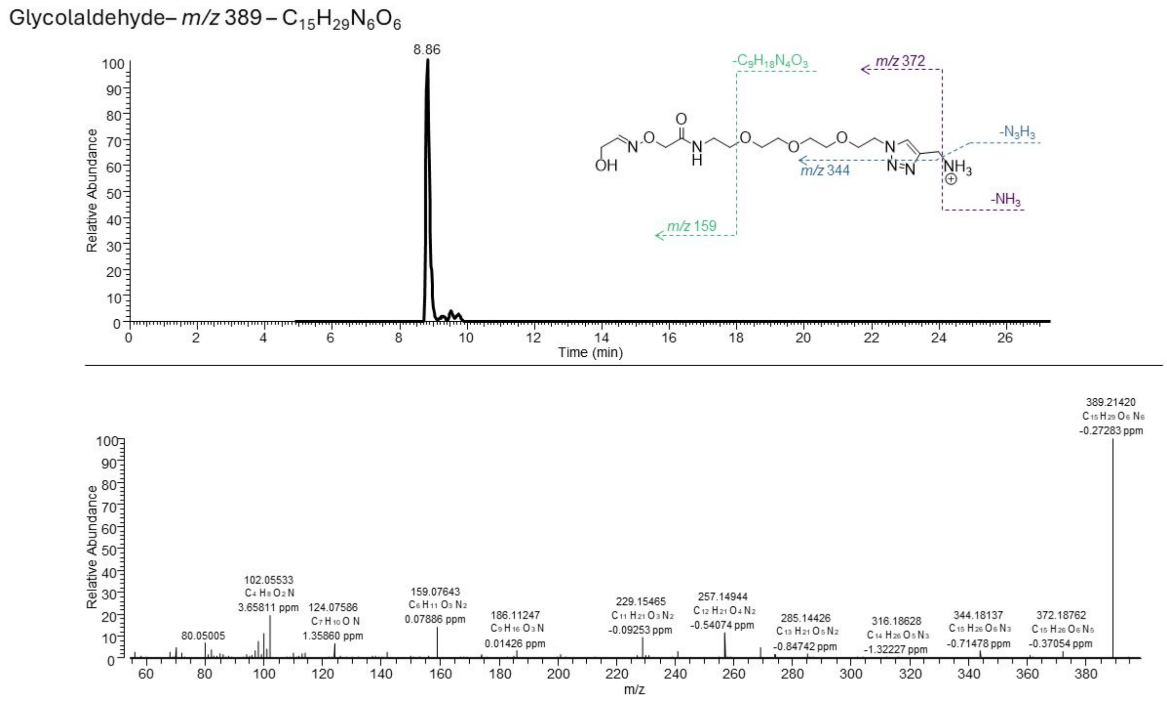

Figure S2.23. Selected ion chromatogram and MS^2 spectrum for the glycolaldehyde Schiff base adduct via ONH_2-bead derivatization (*m/z* 389).

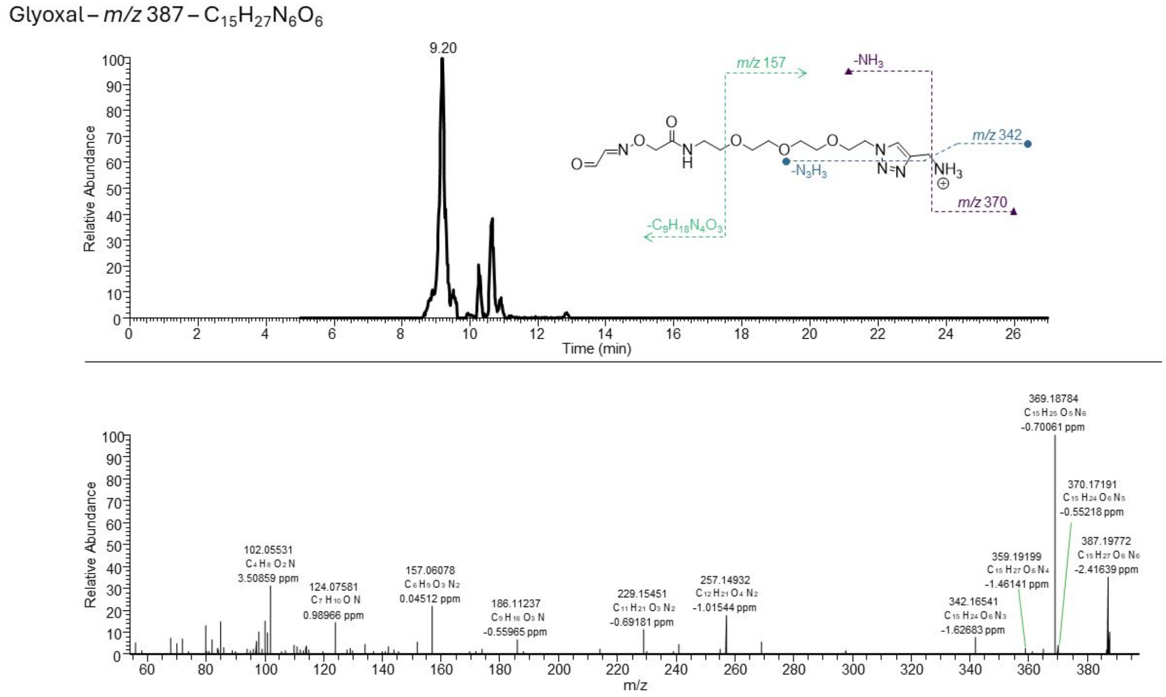

Figure S2.24. Selected ion chromatogram and MS^2 spectrum for the glyoxal Schiff base adduct via ONH_2-bead derivatization (*m/z* 387).

Glyoxal – *m/z* 405 – $C_{15}H_{29}N_6O_7$

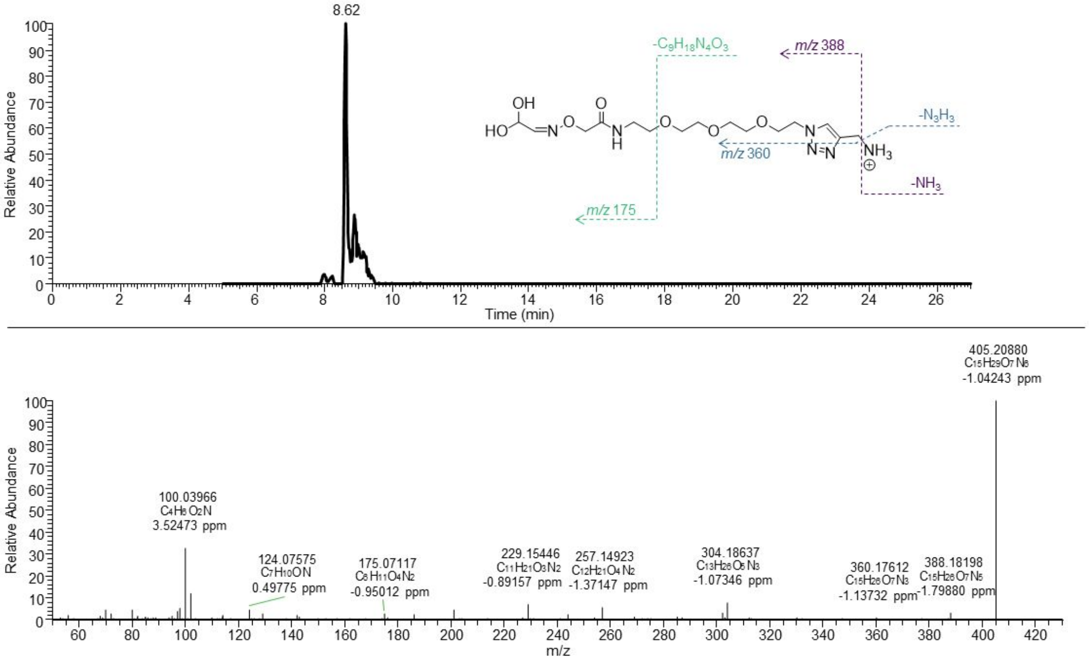

Figure S2.25. Selected ion chromatogram and MS^2 spectrum for the glyoxal hydrate Schiff base adduct via ONH_2-bead derivatization (*m/z* 405).

Glyoxal – *m/z* 463 – $C_{17}H_{31}N_6O_9$

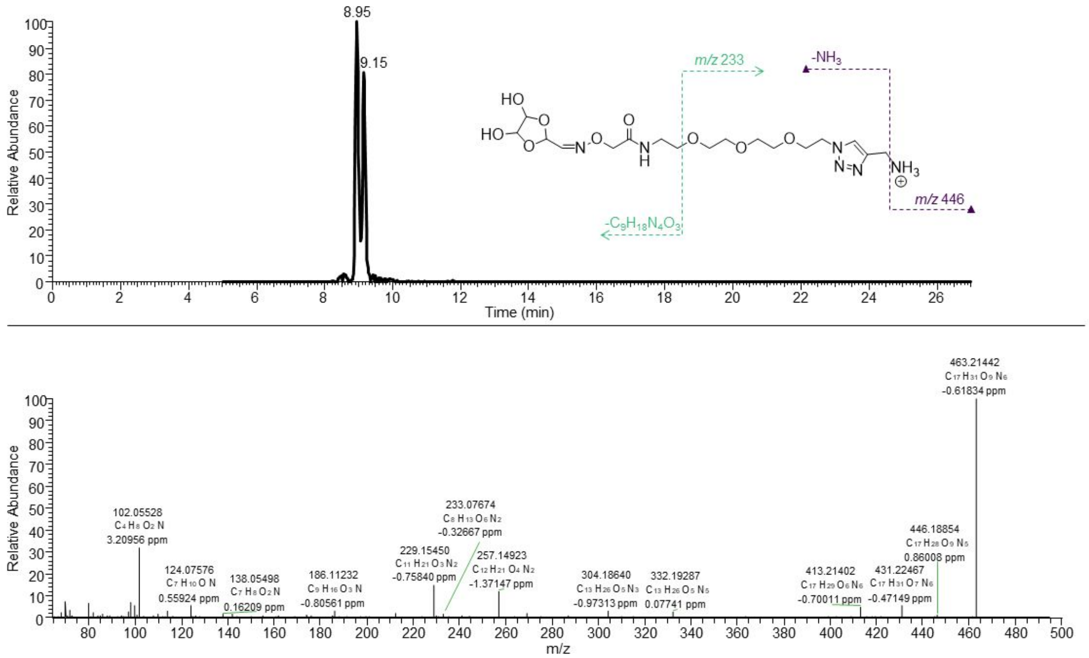

Figure S2.26. Selected ion chromatogram and MS^2 spectrum for the glyoxal dimer Schiff base adduct via ONH_2-bead derivatization (*m/z* 463).

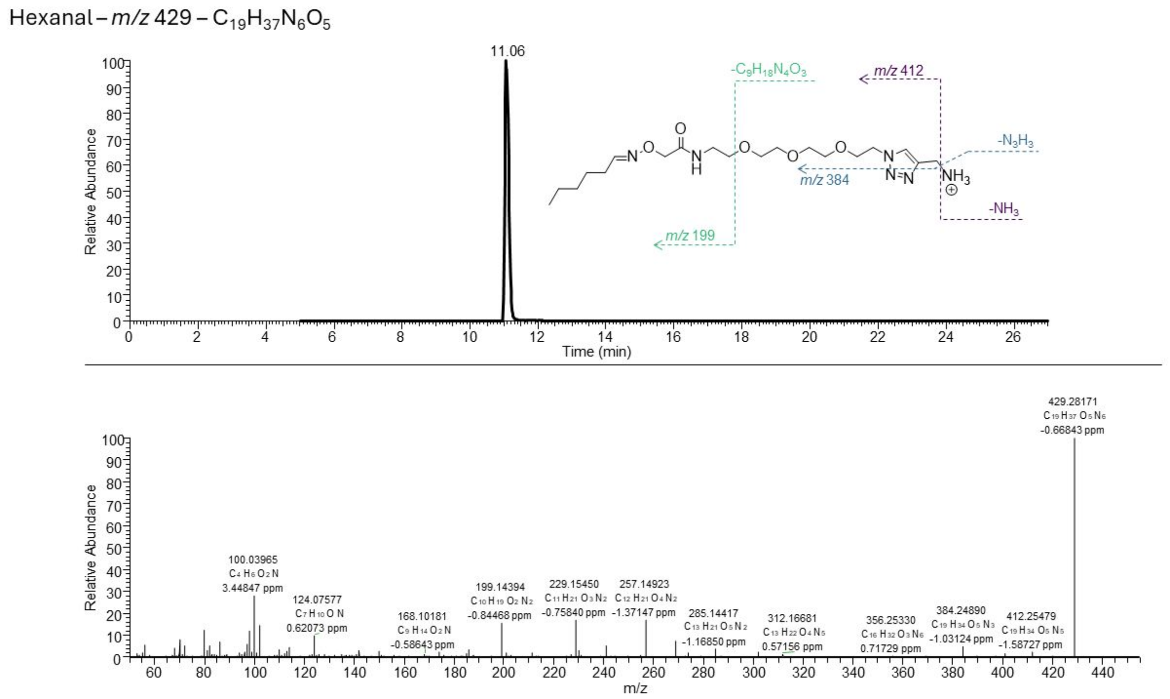

Figure S2.27. Selected ion chromatogram and MS^2 spectrum for the hexanal Schiff base adduct via ONH_2-bead derivatization (*m/z* 429).

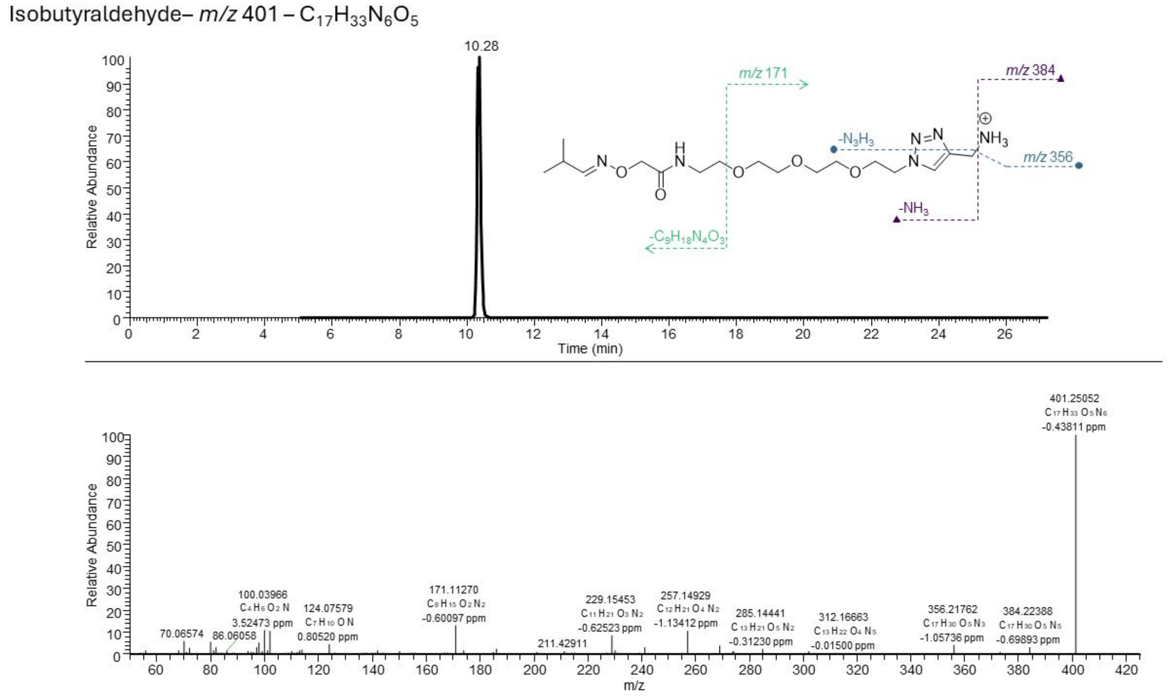

Figure S2.28. Selected ion chromatogram and MS^2 spectrum for the isobutyraldehyde Schiff base adduct via ONH_2-bead derivatization (*m/z* 401).

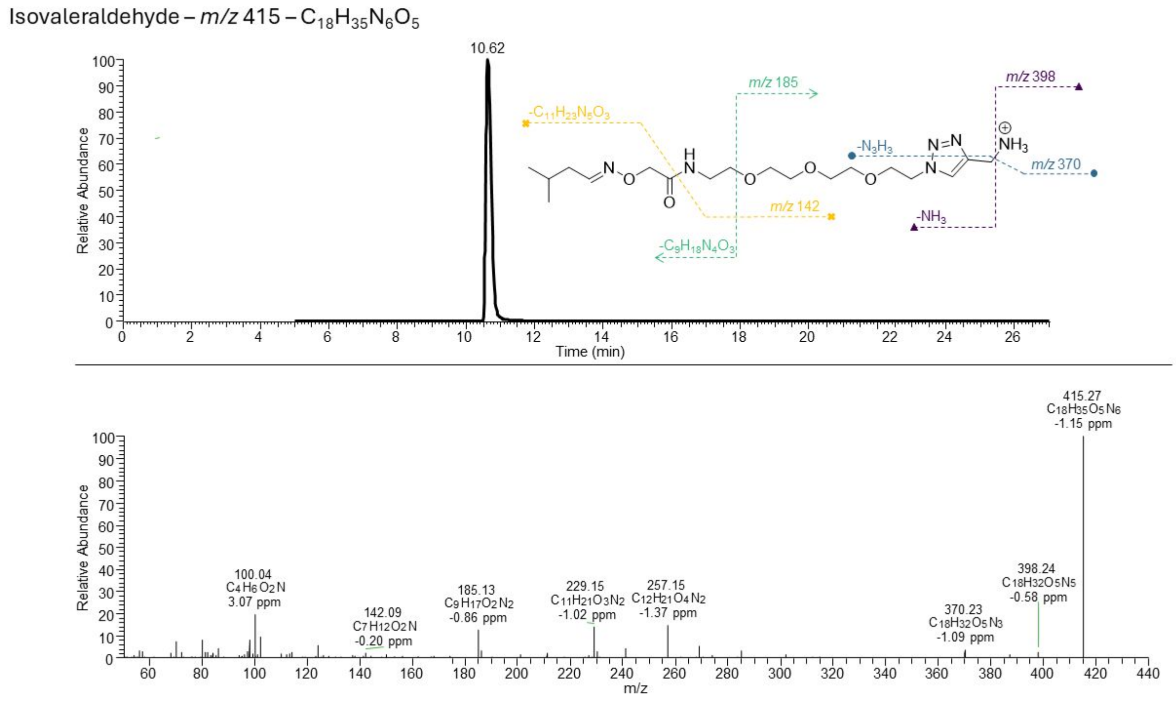

Figure S2.29. Selected ion chromatogram and MS^2 spectrum for the isovaleraldehyde Schiff base adduct via ONH_2-bead derivatization (*m/z* 415).

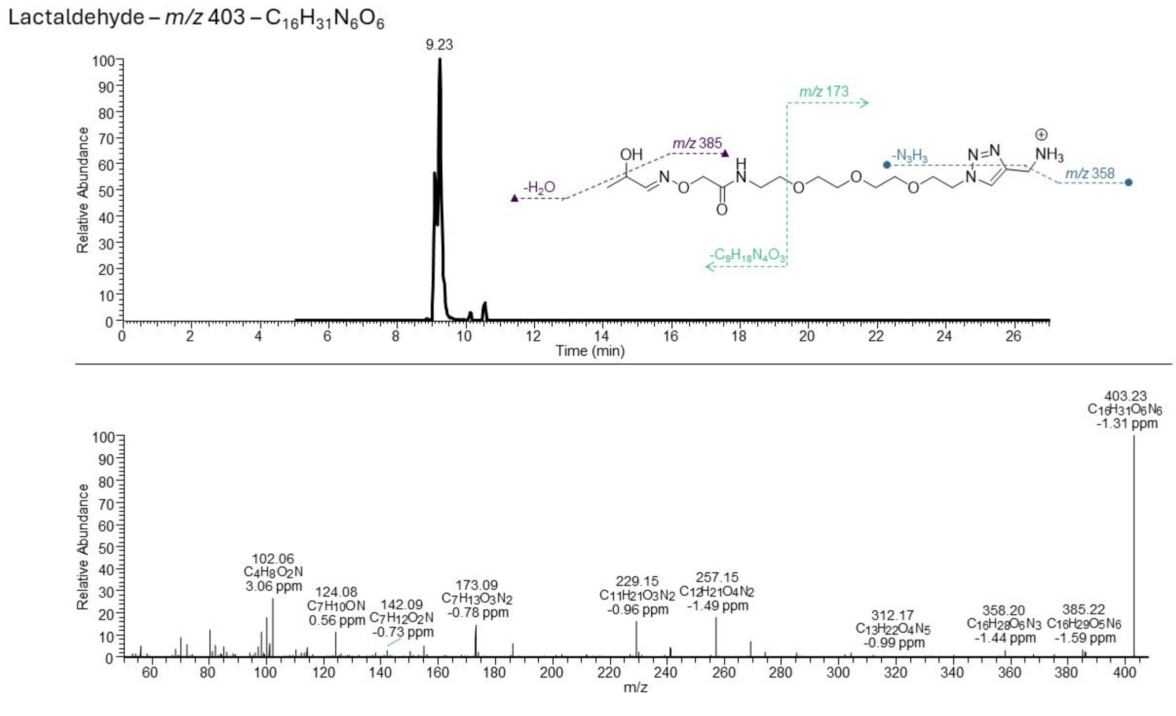

Figure S2.30. Selected ion chromatogram and MS^2 spectrum for the lactaldehyde Schiff base adduct via ONH_2-bead derivatization (*m/z* 403).

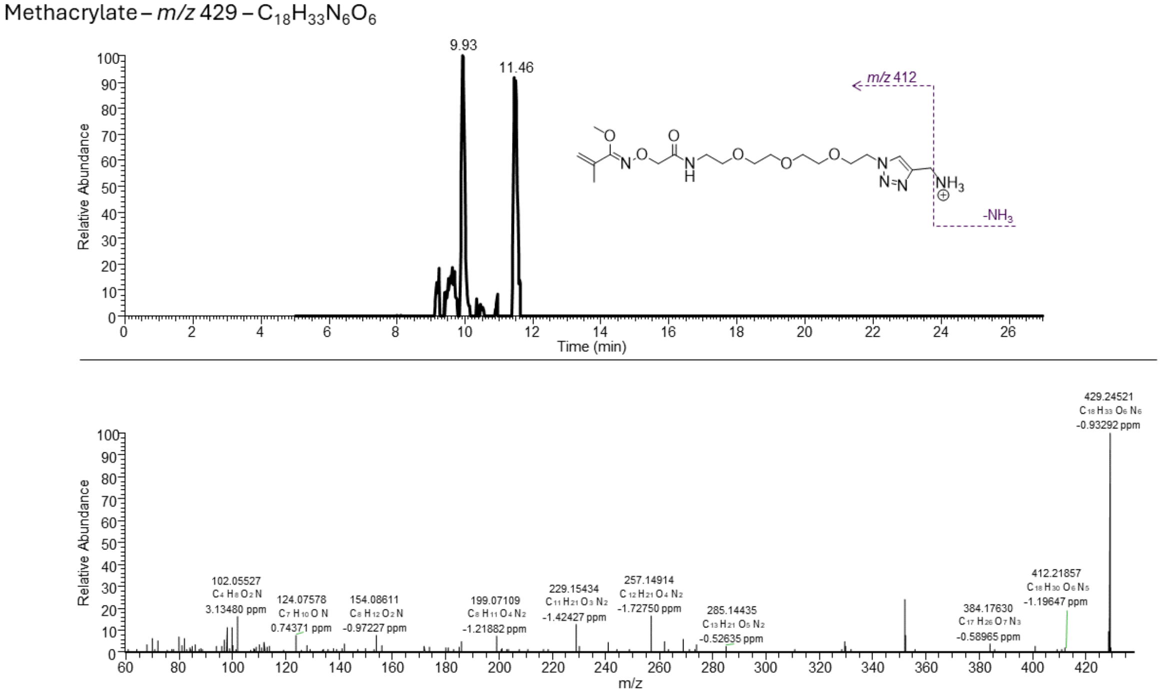

Figure S2.31. Selected ion chromatogram and MS² spectrum for the methacrylate Schiff base adduct via ONH_2-bead derivatization (*m/z* 429).

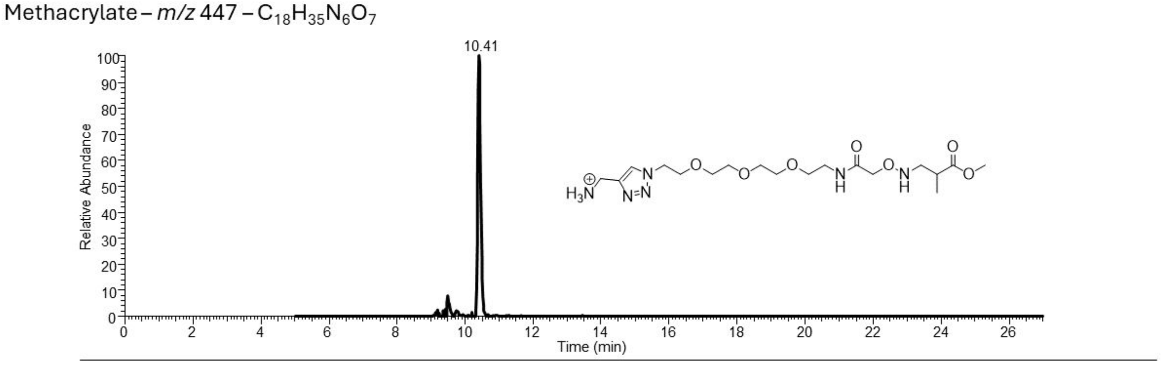

Figure S2.32. Selected ion chromatogram for the methacrylate Michael addition adduct via ONH_2-bead derivatization (*m/z* 447). MS² information was not available.

Methional – *m/z* 433 – $C_{17}H_{33}N_6O_5S$

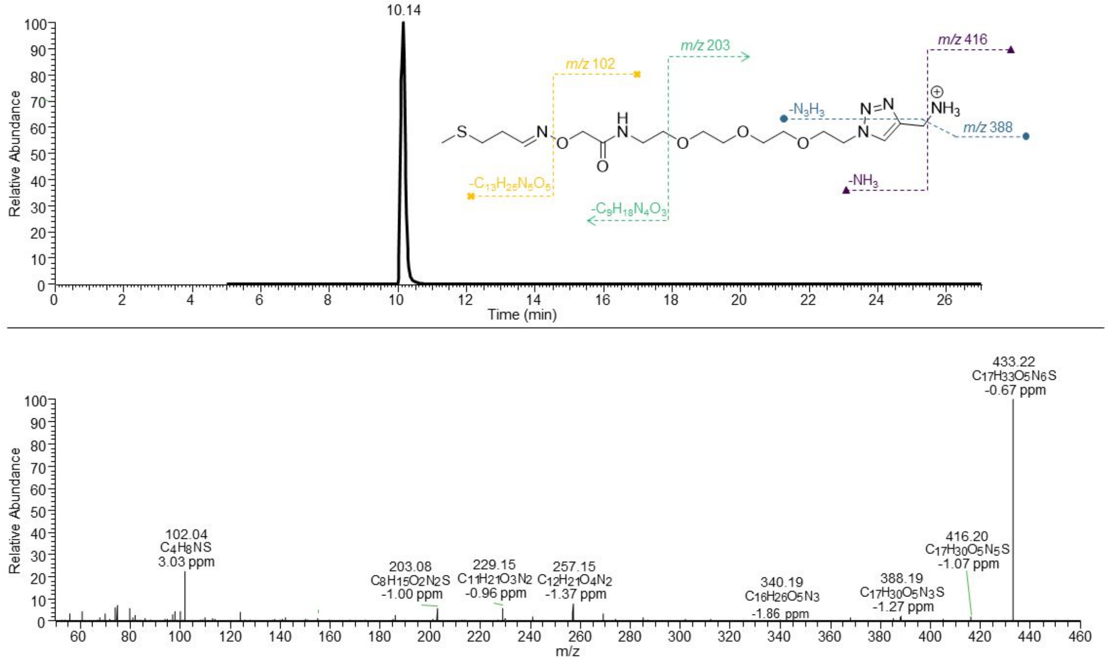

Figure S2.33. Selected ion chromatogram and MS^2 spectrum for the methional Schiff base adduct via ONH_2-bead derivatization (*m/z* 433).

3-methylsulfinylpropanal – *m/z* 449 – $C_{17}H_{33}N_6O_6S$

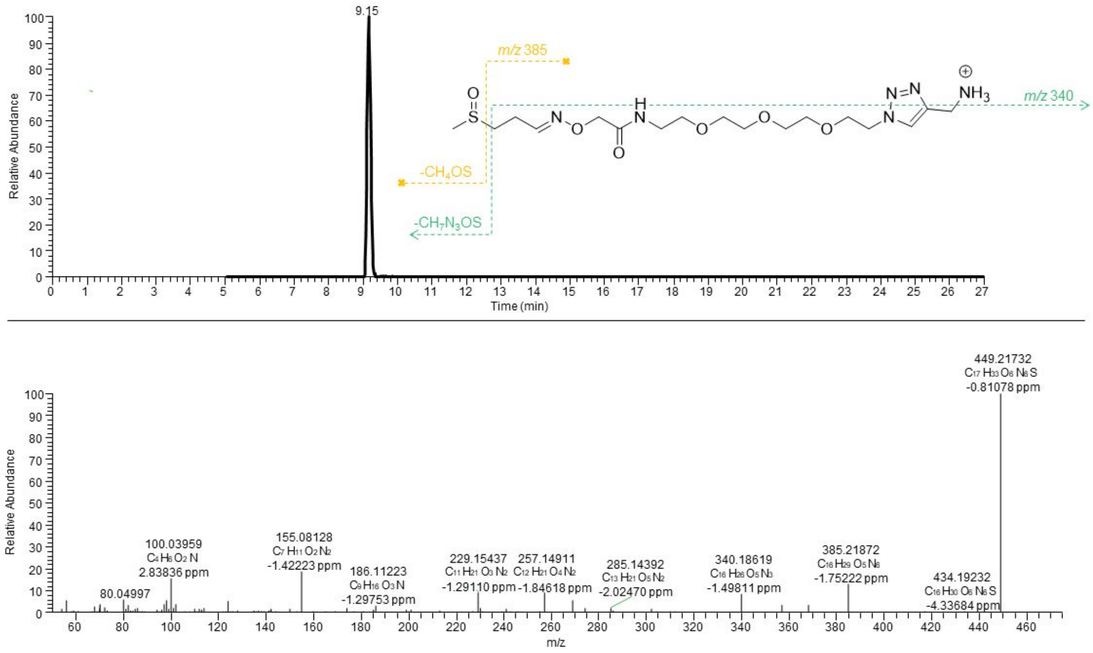

Figure S2.34. Selected ion chromatogram and MS^2 spectrum for the 3-methylfulfinylpropanal Schiff base adduct via ONH_2-bead derivatization (*m/z* 449).

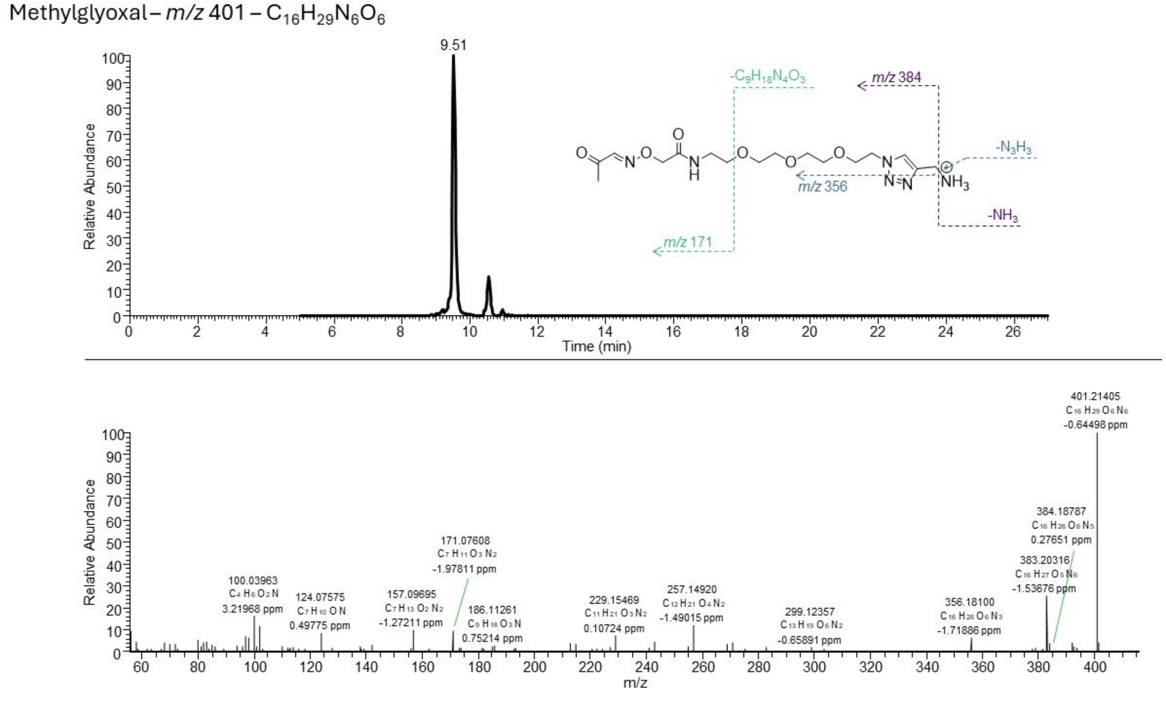

Figure S2.35. Selected ion chromatogram and MS^2 spectrum for the methylglyoxal Schiff base adduct via ONH_2-bead derivatization (*m/z* 401).

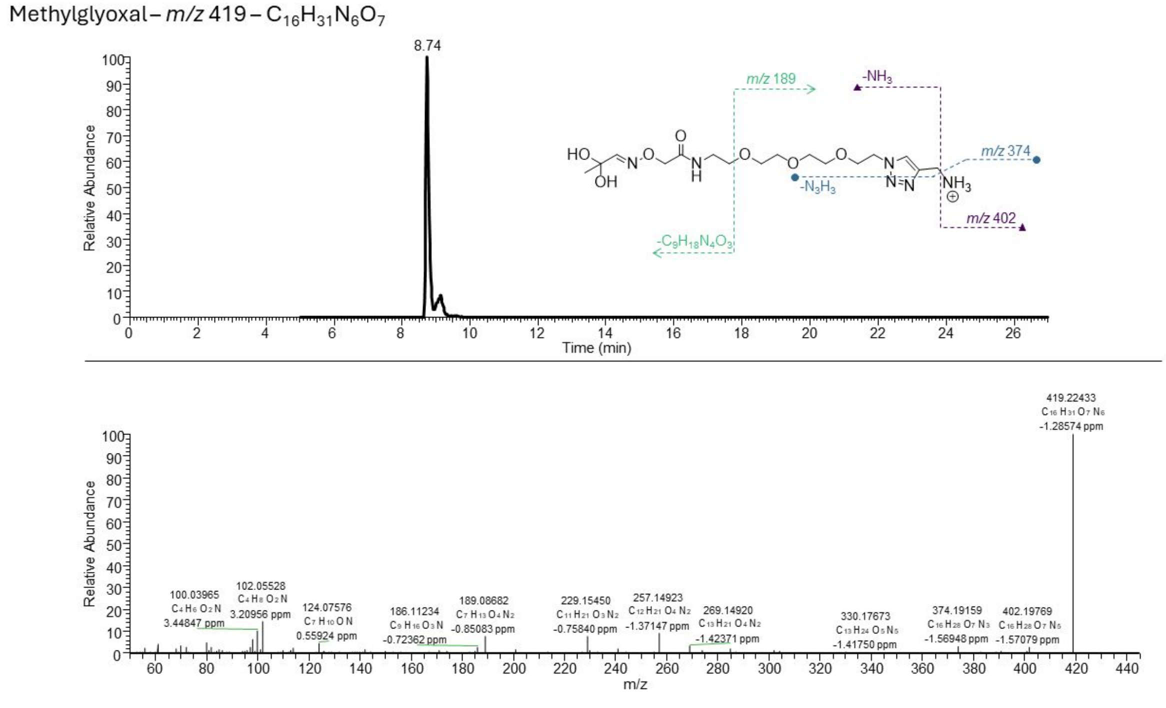

Figure S2.36. Selected ion chromatogram and MS^2 spectrum for the methylglyoxal hydrate Schiff base adduct via ONH_2-bead derivatization (*m/z* 419).

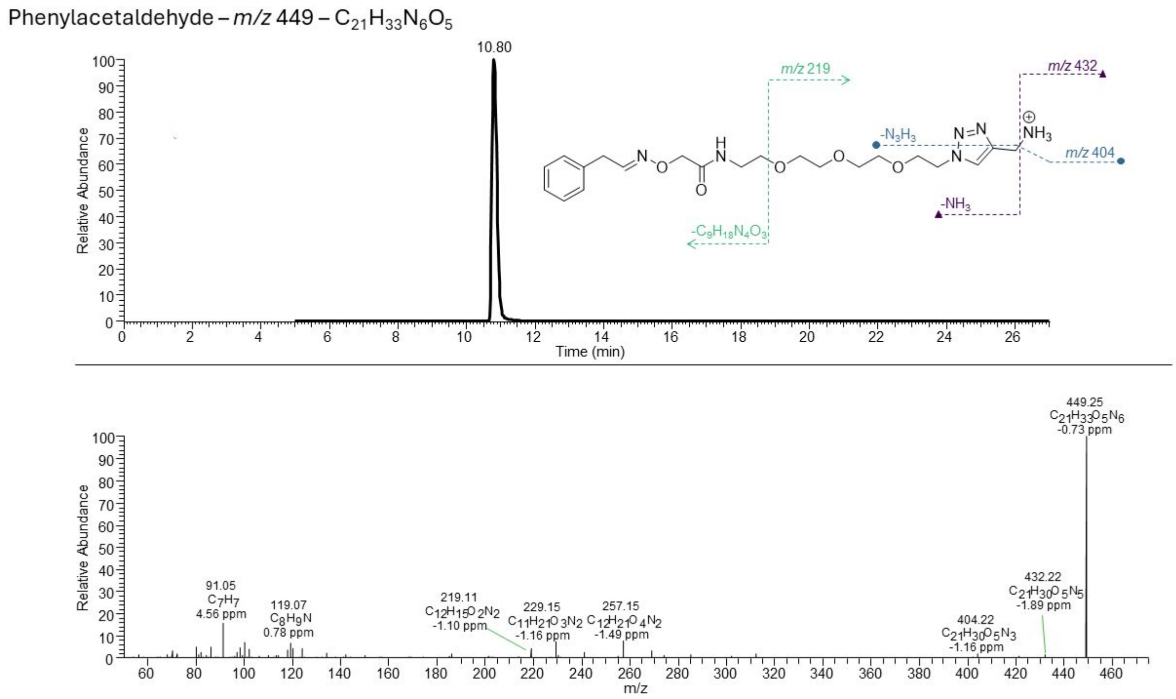

Figure S2.37. Selected ion chromatogram and MS^2 spectrum for the phenylacetaldehyde Schiff base adduct via ONH_2-bead derivatization (*m/z* 449).

Part 2: Hydrazide (NH_2) bead system

2-butenedial – *m/z* 427 – $C_{18}H_{31}N_6O_6$

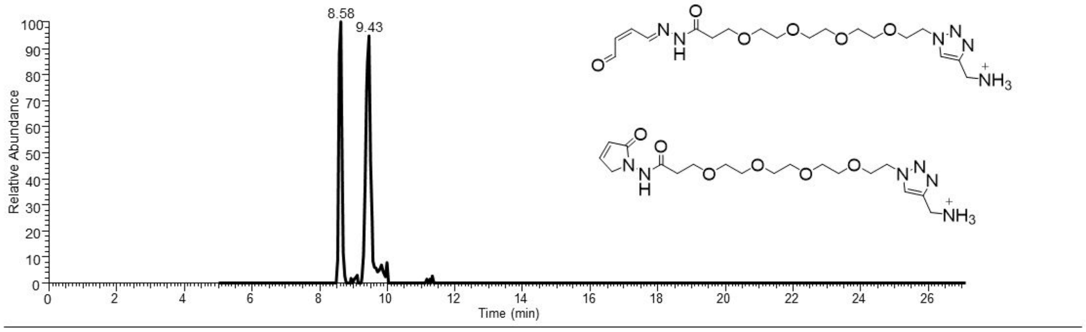

Figure S2.38. Selected ion chromatogram for the 2-butene-1,4-dial Schiff base adduct via NNH_2-bead derivatization (*m/z* 427). MS^2 information was not available.

2-butenedial – *m/z* 445 – $C_{18}H_{33}N_6O_7$

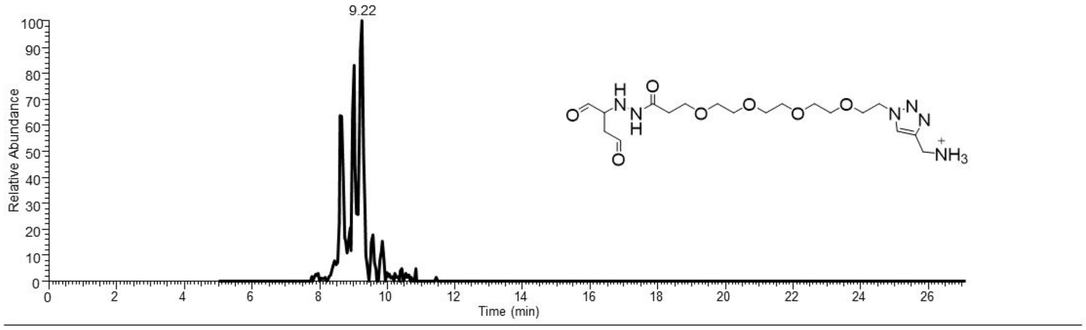

Figure S2.39. Selected ion chromatogram for the 2-butene-1,4-dial Michael addition adduct via NNH_2-bead derivatization (*m/z* 445). MS^2 information was not available.

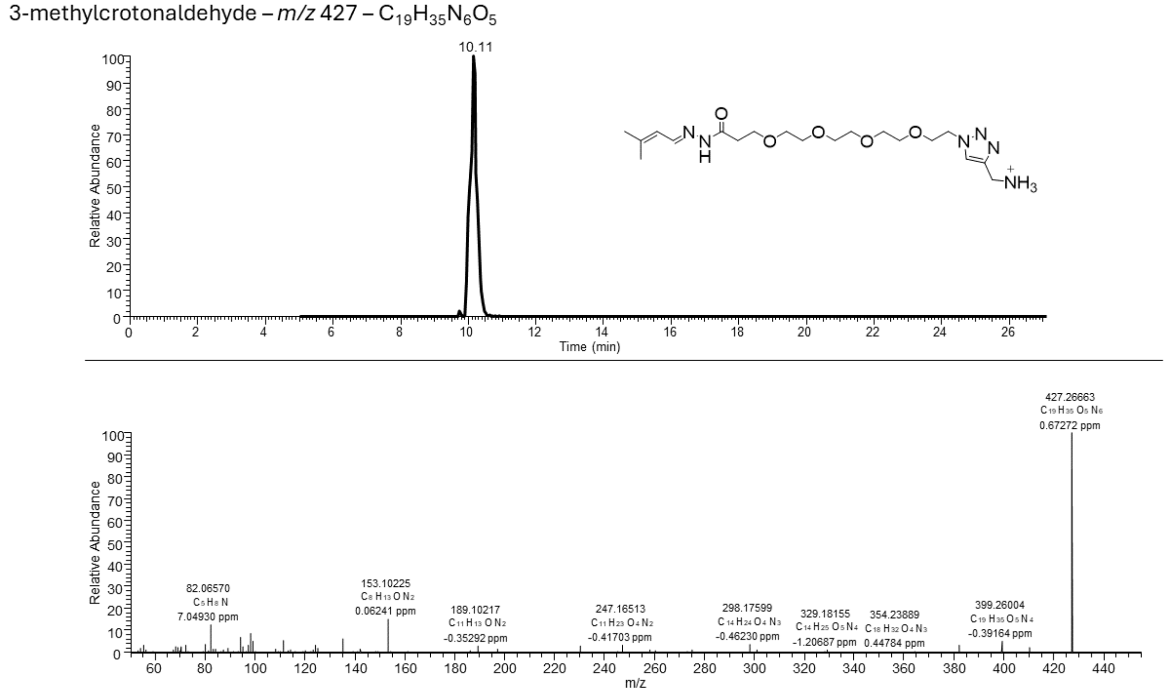

Figure S2.40. Selected ion chromatogram and MS^2 spectrum for the 3-methylcrotonaldehyde Schiff base adduct via NNH_2-bead derivatization (*m/z* 427).

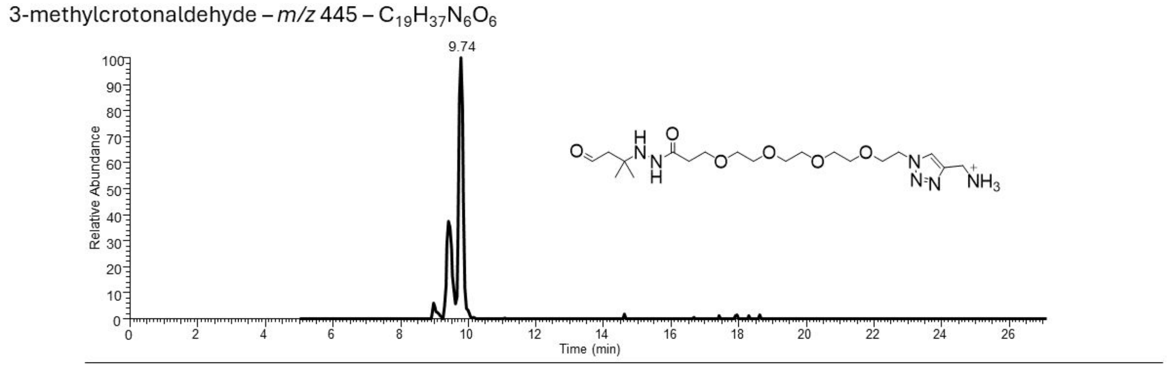

Figure S2.41. Selected ion chromatogram for the 3-methylcrotonaldehyde Michael addition adduct via NNH_2-bead derivatization (*m/z* 445). MS^2 information was not available.

Acrolein – *m/z* 399 – $C_{17}H_{31}N_6O_5$

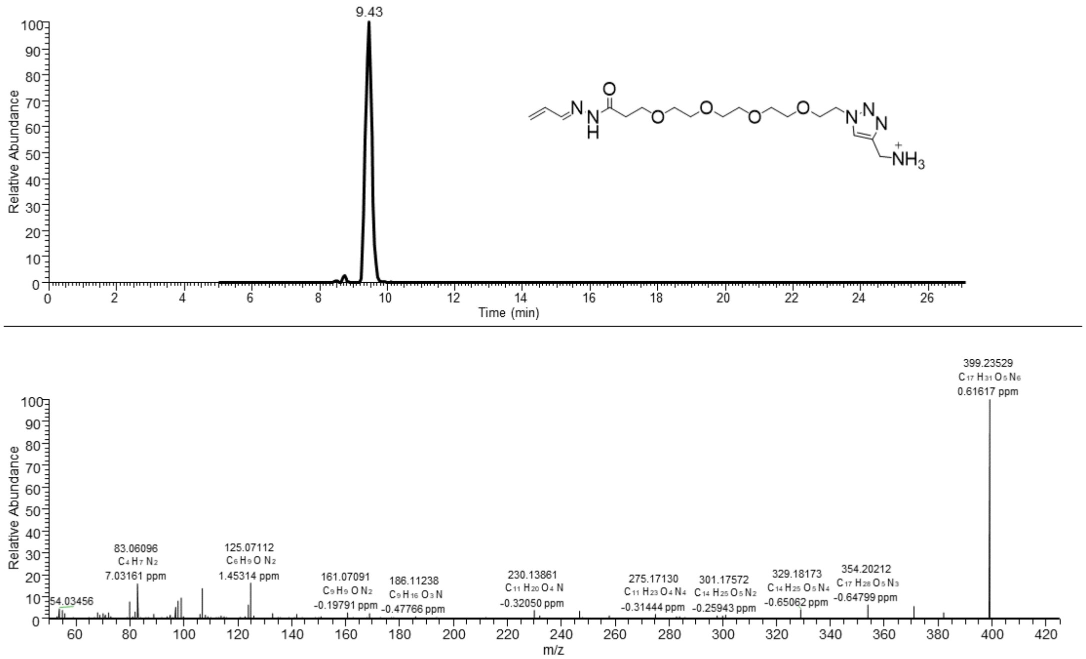

Figure S2.42. Selected ion chromatogram and MS[2] spectrum for the acrolein Schiff base adduct via NNH_2-bead derivatization (*m/z* 399).

Acrolein – *m/z* 417 – $C_{17}H_{33}N_6O_6$

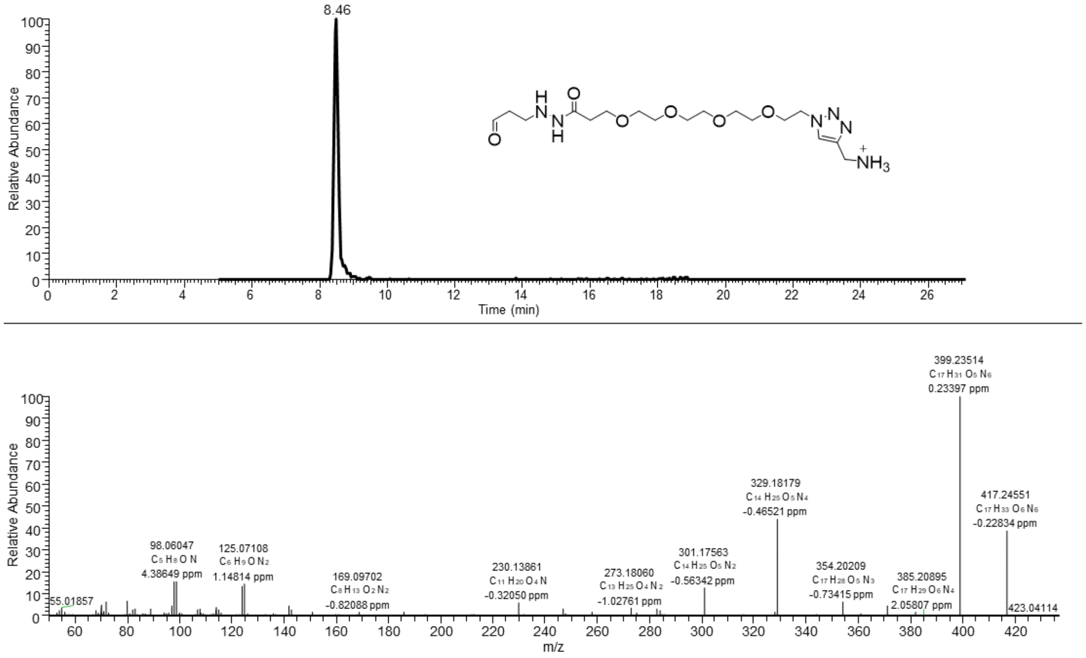

Figure S2.43. Selected ion chromatogram and MS[2] spectrum for the acrolein Michael addition adduct via NNH_2-bead derivatization (*m/z* 417).

Benzaldehyde – *m/z* 449 – $C_{21}H_{33}N_6O_5$

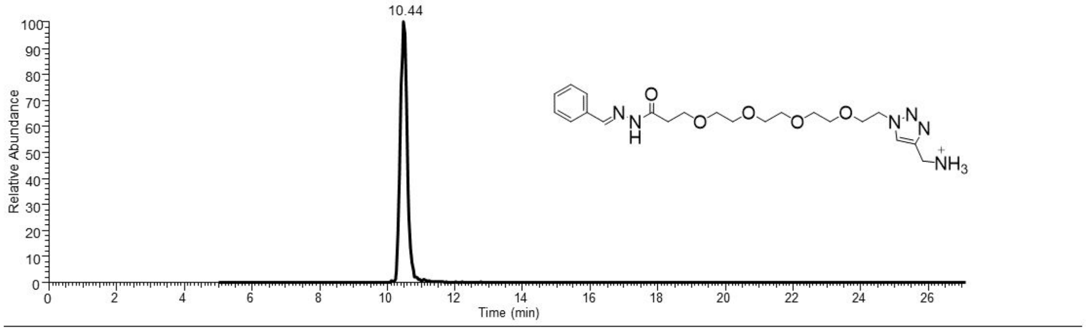

Figure S2.44. Selected ion chromatogram for the benzaldehyde Schiff base adduct via NNH_2-bead derivatization (*m/z* 449). MS^2 information was not available.

Benzaldehyde-d5 – *m/z* 454 – $C_{21}H_{28}D_5N_6O_5$

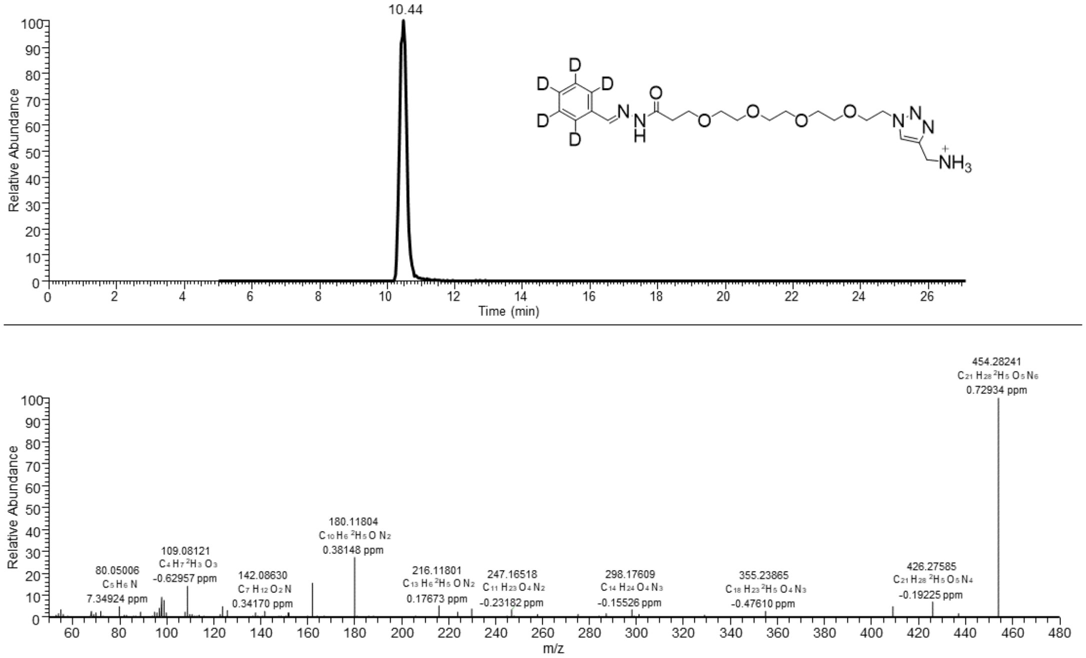

Figure S2.45. Selected ion chromatogram and MS^2 spectrum for the benzaldehyde-d5 Schiff base adduct via NNH_2-bead derivatization (*m/z* 454).

Butanal – *m/z* 415 – $C_{18}H_{35}N_6O_5$

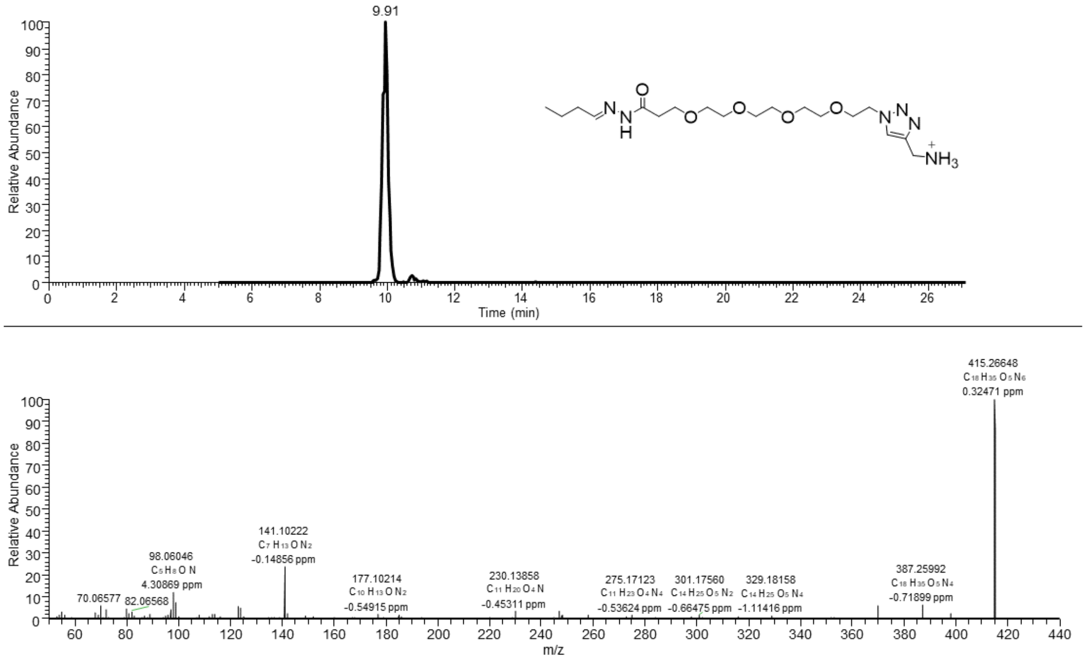

Figure S2.46. Selected ion chromatogram and MS^2 spectrum for the butanal Schiff base adduct via NNH_2-bead derivatization (*m/z* 415).

Butanal-d2 – *m/z* 417 – $C_{18}H_{33}D_2N_6O_5$

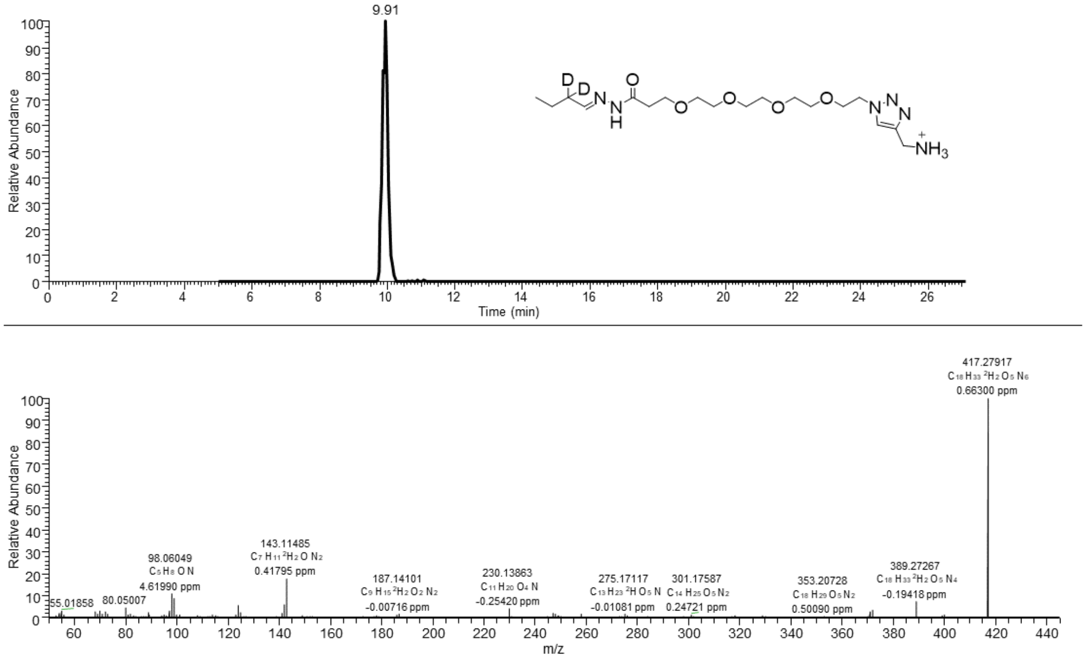

Figure S2.47. Selected ion chromatogram and MS^2 spectrum for the butanal-d2 Schiff base adduct via NNH_2-bead derivatization (*m/z* 417).

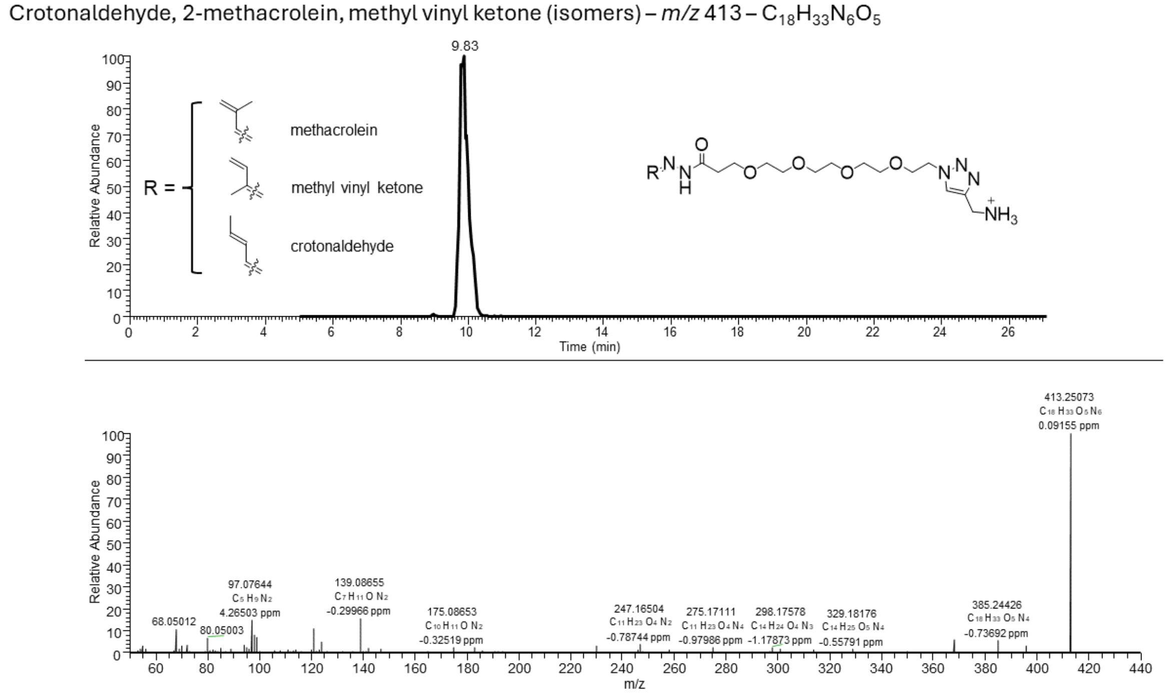

Figure S2.48. Selected ion chromatogram and MS[2] spectrum for the crotonaldehyde/2-methacrolein/methyl vinyl ketone (isomers) Schiff base adduct via NNH_2-bead derivatization (*m/z* 413).

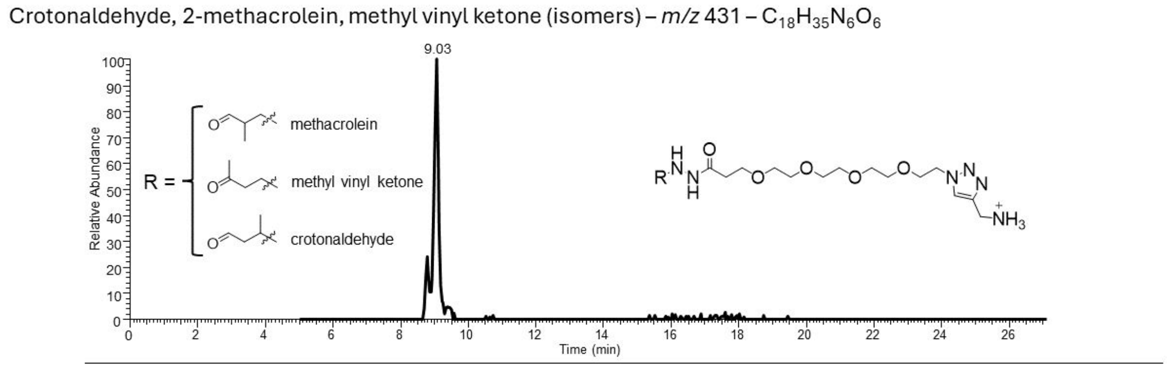

Figure S2.49. Selected ion chromatogram for the crotonaldehyde/2-methacrolein/methyl vinyl ketone (isomers) Michael addition adduct via NNH_2-bead derivatization (*m/z* 431). MS[2] information was not available.

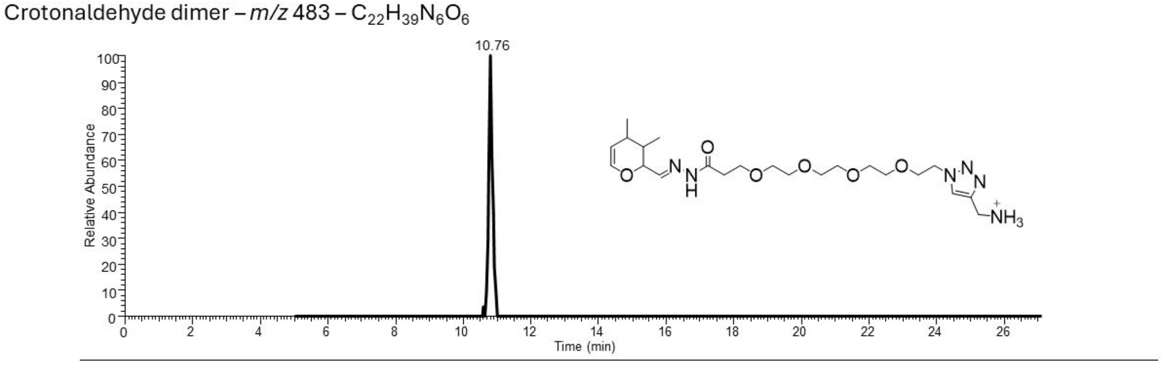

Figure S2.50. Selected ion chromatogram for the crotonaldehyde dimer Schiff base adduct via NNH_2-bead derivatization (*m/z* 483). MS^2 information was not available.

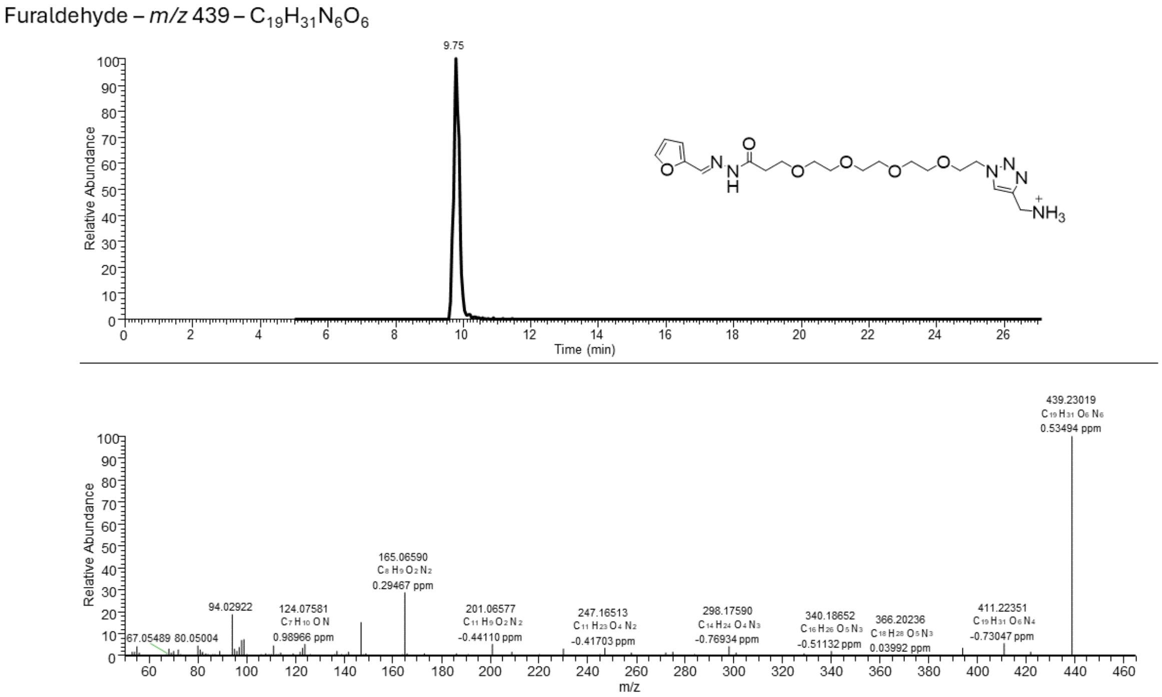

Figure S2.51. Selected ion chromatogram and MS^2 spectrum for the furaldehyde Schiff base adduct via NNH_2-bead derivatization (*m/z* 439).

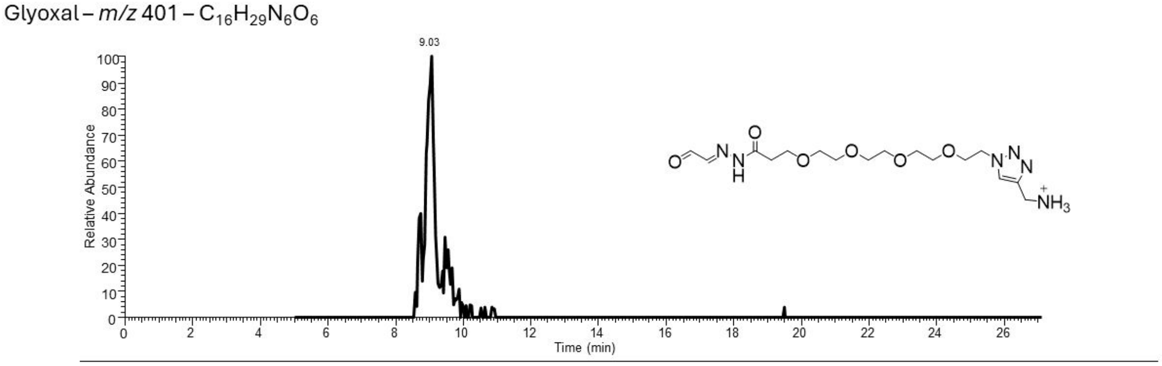

Figure S2.52. Selected ion chromatogram for the glyoxal Schiff base adduct via NNH_2-bead derivatization (*m/z* 401). MS^2 information was not available.

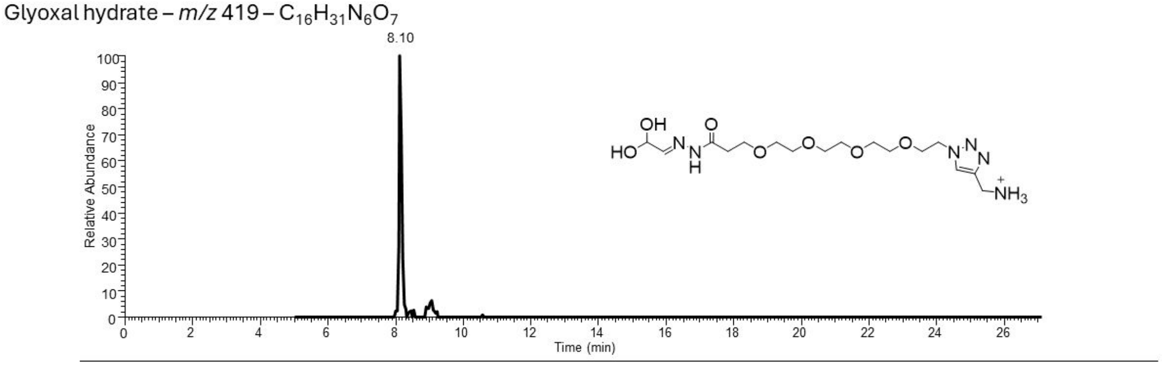

Figure S2.53. Selected ion chromatogram for the glyoxal hydrate Schiff base adduct via NNH_2-bead derivatization (*m/z* 419). MS^2 information was not available.

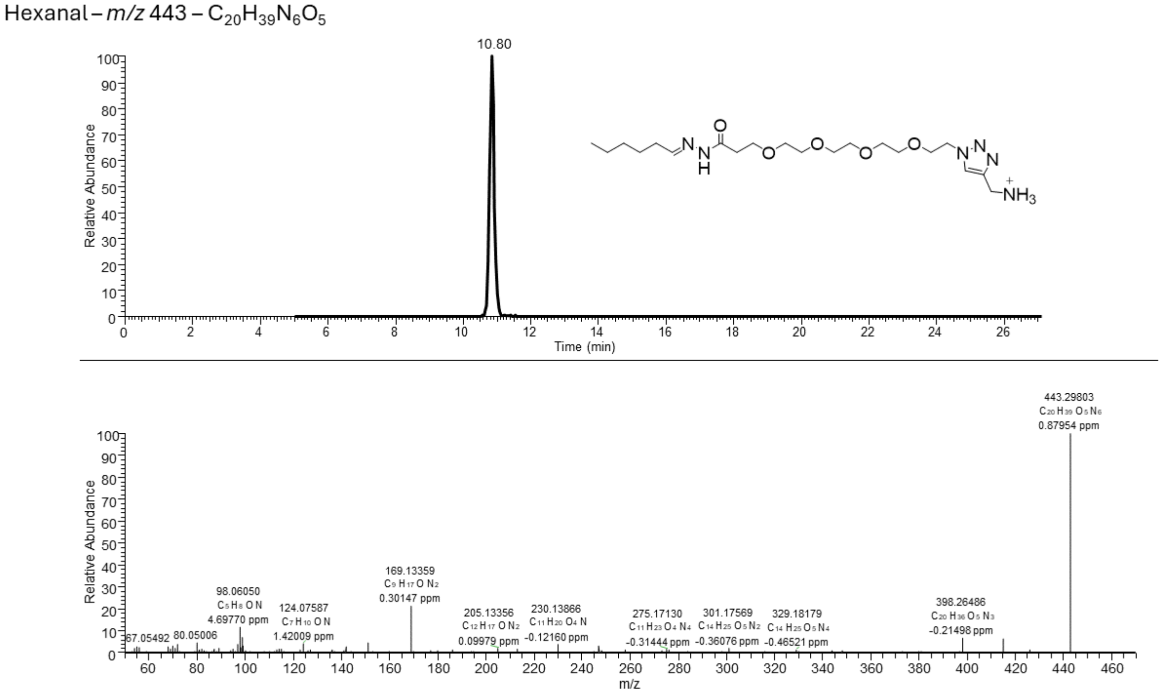

Figure S2.54. Selected ion chromatogram and MS^2 spectrum for the hexanal Schiff base adduct via NNH_2-bead derivatization (*m/z* 443).

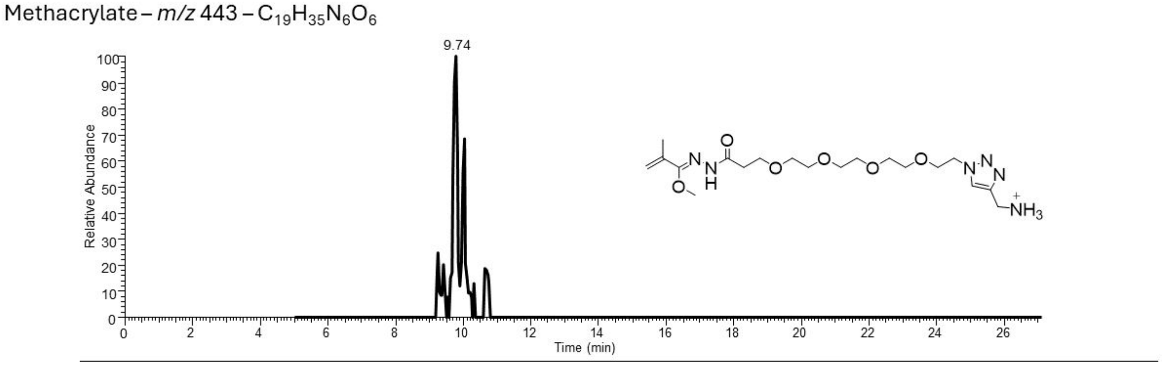

Figure S2.55. Selected ion chromatogram for the methacrylate Schiff base adduct via NNH_2-bead derivatization (*m/z* 443). MS^2 information was not available.

Methacrylate – *m/z* 461 – $C_{19}H_{37}N_6O_7$

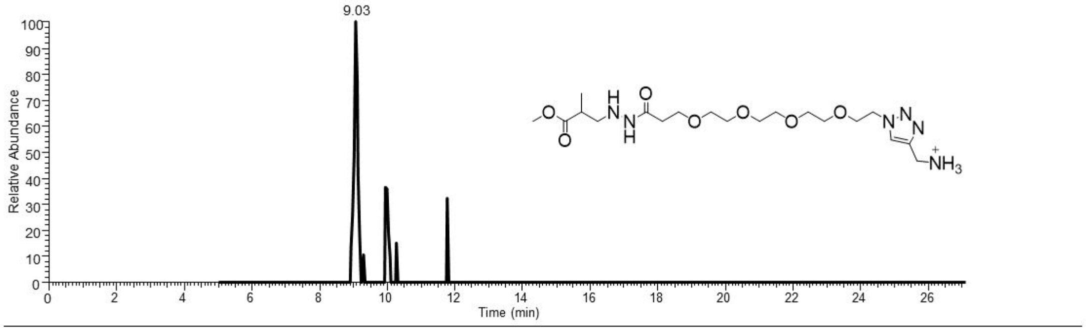

Figure S2.56. Selected ion chromatogram for the methacrylate Michael addition adduct via NNH_2-bead derivatization (*m/z* 461). MS^2 information was not available.

Methylglyoxal – *m/z* 415 (RT 9.11 min) – $C_{17}H_{31}N_6O_6$

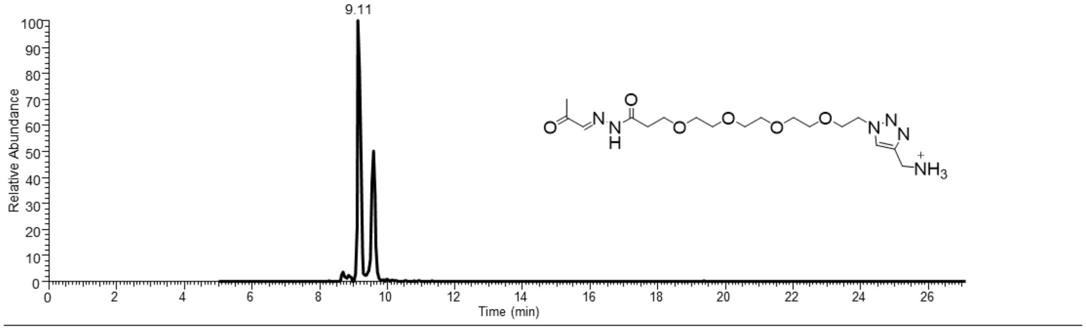

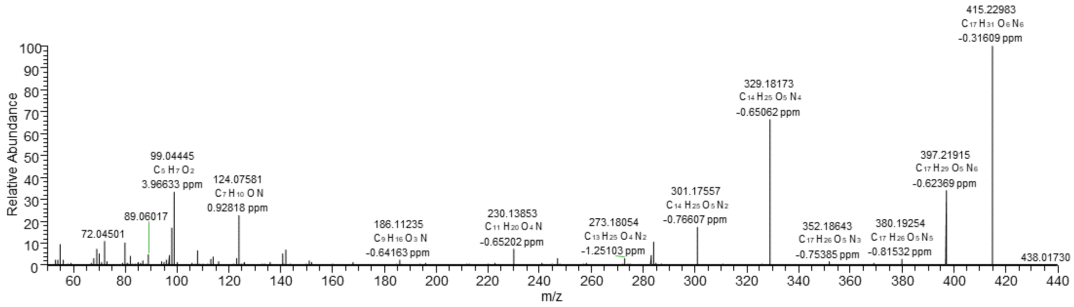

Figure S2.57. Selected ion chromatogram and MS^2 spectrum for the methylglyoxal Schiff base adduct via NNH_2-bead derivatization (*m/z* 415). RT = retention time.

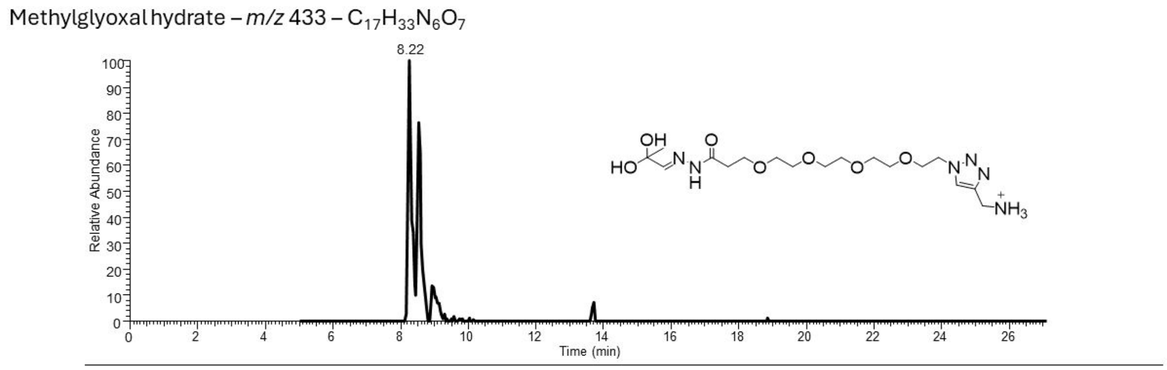

Figure S2.58. Selected ion chromatogram for the methylglyoxal hydrate Schiff base adduct via NNH_2-bead derivatization (*m/z* 433). MS^2 information was not available.

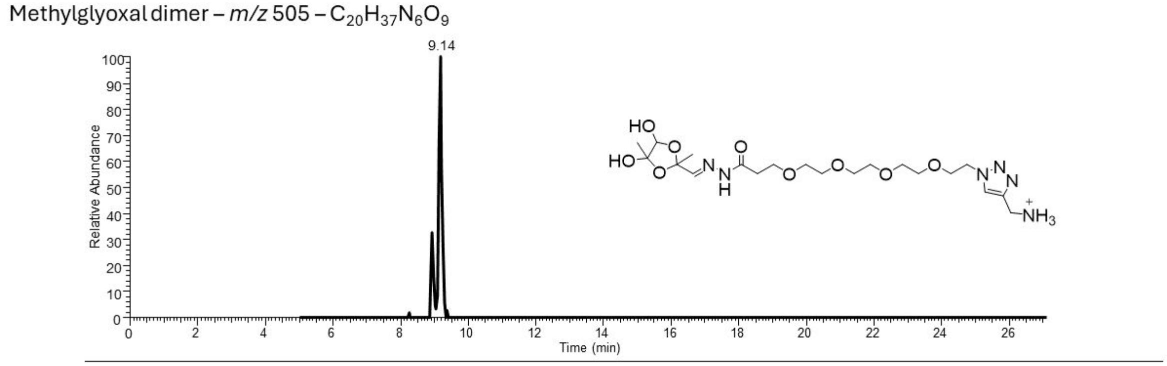

Figure S2.59. Selected ion chromatogram for the methylglyoxal dimer Schiff base adduct via NNH_2-bead derivatization (*m/z* 505). MS^2 information was not available.

Part 3: Amine (NH_2) bead system

2-butenedial – *m/z* 340 – $C_{15}H_{26}N_5O_4$

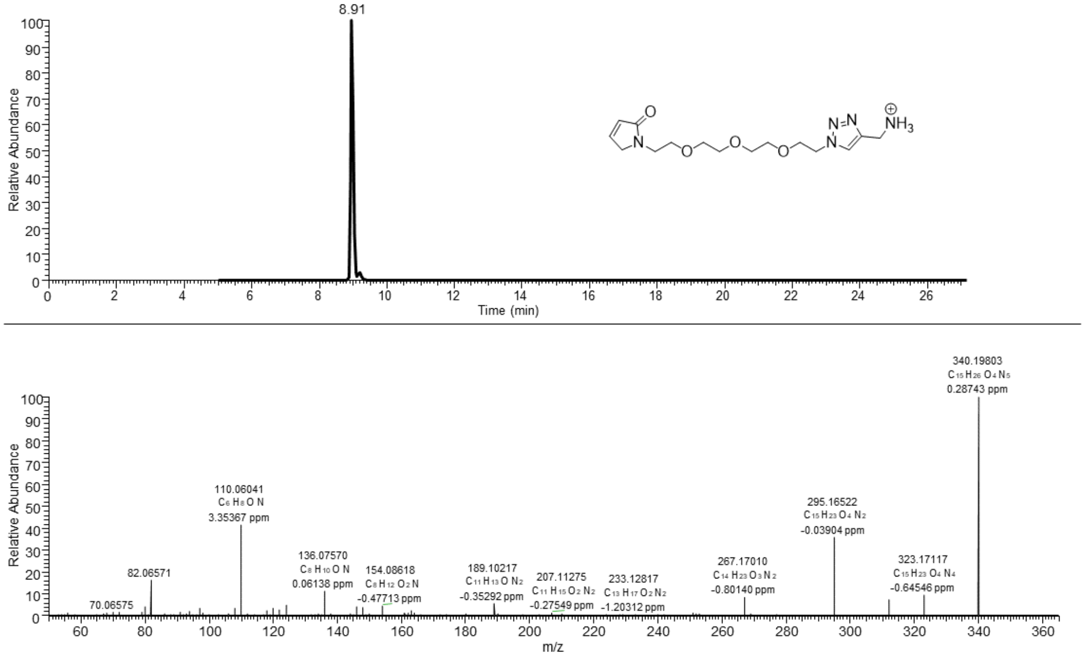

Figure S2.60. Selected ion chromatogram and MS^2 spectrum for the 2-butene-1,4-dial Schiff base adduct via NH_2-bead derivatization (*m/z* 340).

Glyoxal – *m/z* 314 – $C_{13}H_{24}N_5O_4$

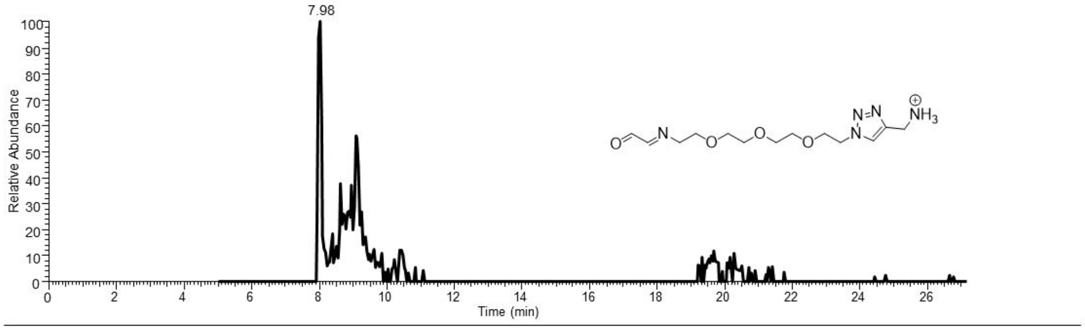

Figure S2.61. Selected ion chromatogram for the glyoxal Schiff base adduct via NH_2-bead derivatization (*m/z* 314). MS^2 information was not available.

Part 4: Reduced amine ($NH_2 + NaBH_3CN$) bead system

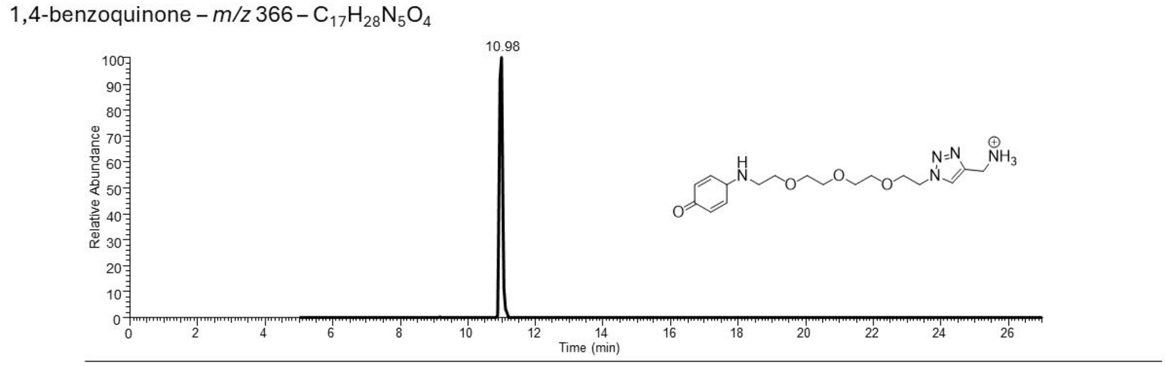

Figure S2.62. Selected ion chromatogram for the 1,4-benzoquinone Schiff base adduct via reduced NH_2-bead derivatization (*m/z* 366). MS^2 information was not available.

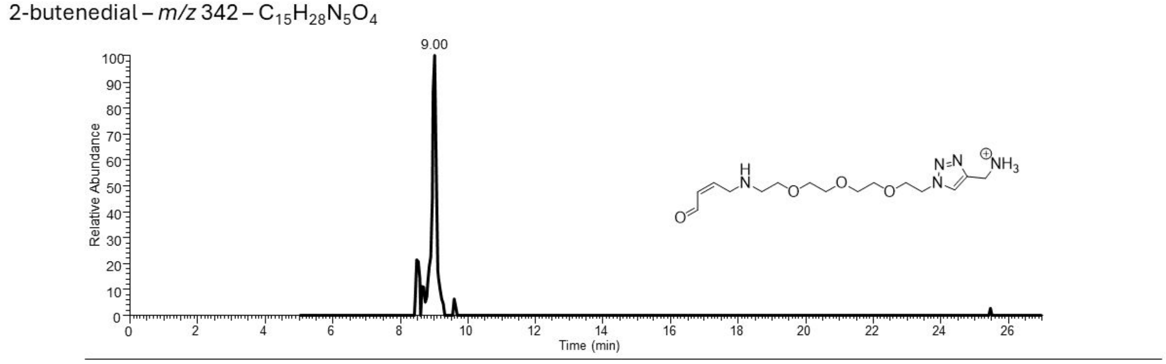

Figure S2.63. Selected ion chromatogram for the 2-butene-1,4-dial Schiff base adduct via reduced NH_2-bead derivatization (*m/z* 342). MS^2 information was not available.

Butanal – *m/z* 330 – $C_{15}H_{32}N_5O_3$

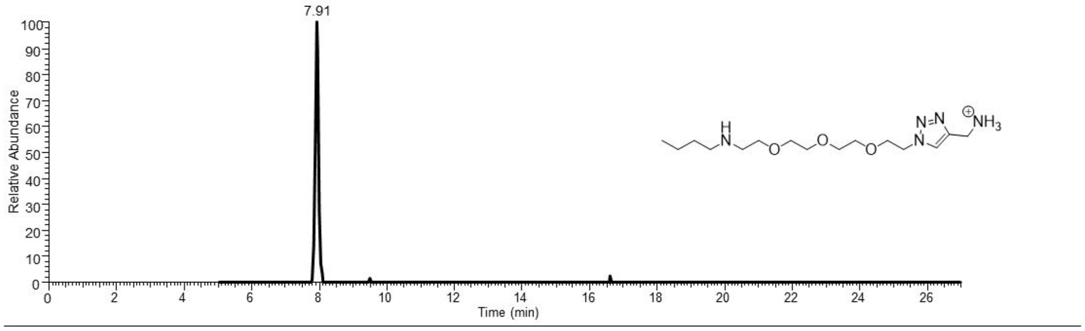

Figure S2.64. Selected ion chromatogram for the butanal Schiff base adduct via reduced NH_2-bead derivatization (*m/z* 330). MS^2 information was not available.

Butanal-d2 – *m/z* 332 – $C_{15}H_{30}D_2N_5O_3$

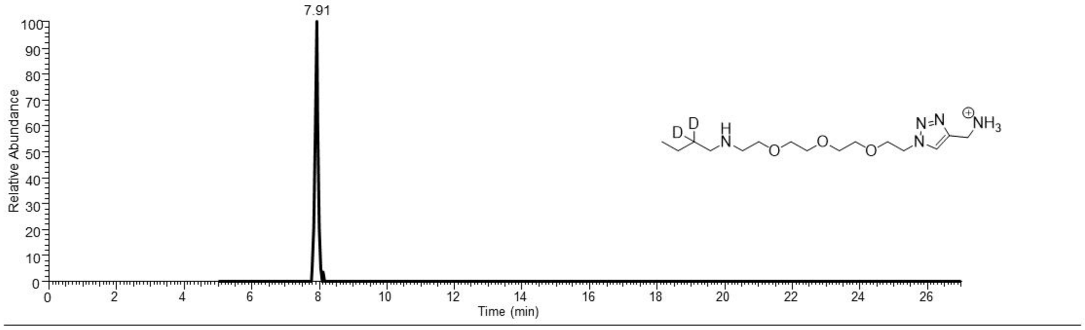

Figure S2.65. Selected ion chromatogram for the butanal-d2 Schiff base adduct via reduced NH_2-bead derivatization (*m/z* 332). MS^2 information was not available.

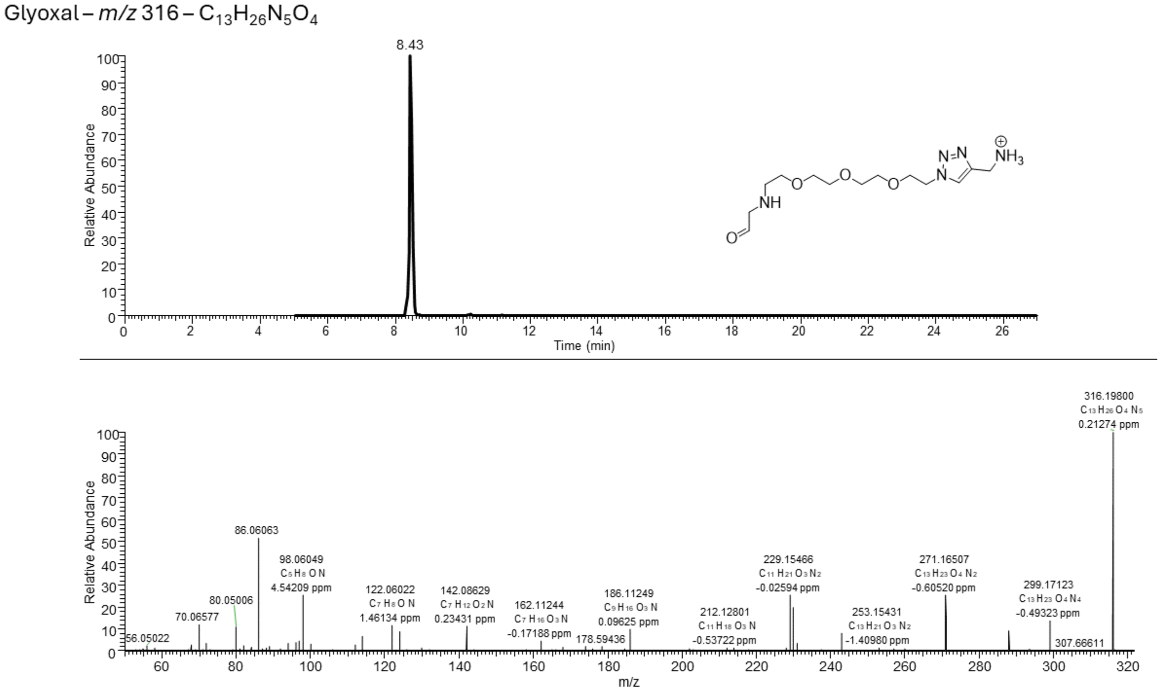

Figure S2.66. Selected ion chromatogram and MS^2 spectrum for the glyoxal Schiff base adduct via reduced NH_2-bead derivatization (*m/z* 316).

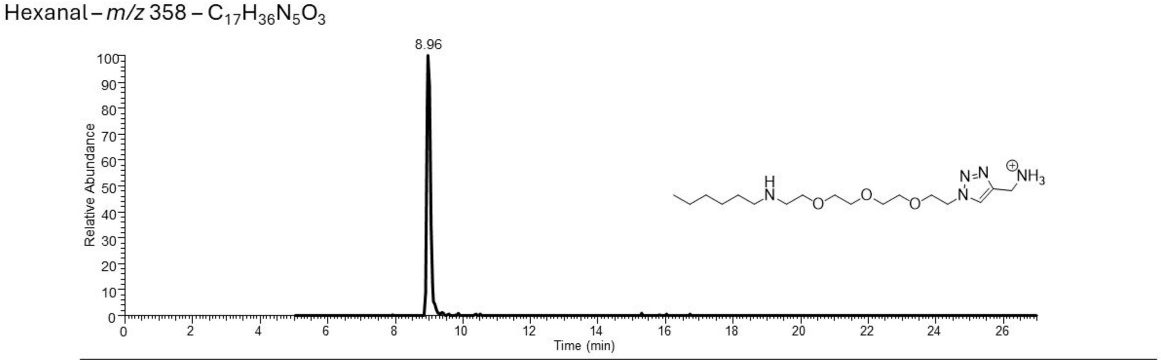

Figure S2.67. Selected ion chromatogram for the hexanal Schiff base adduct via reduced NH_2-bead derivatization (*m/z* 358). MS^2 information was not available.

Methylglyoxal – *m/z* 330 – $C_{14}H_{28}N_5O_4$

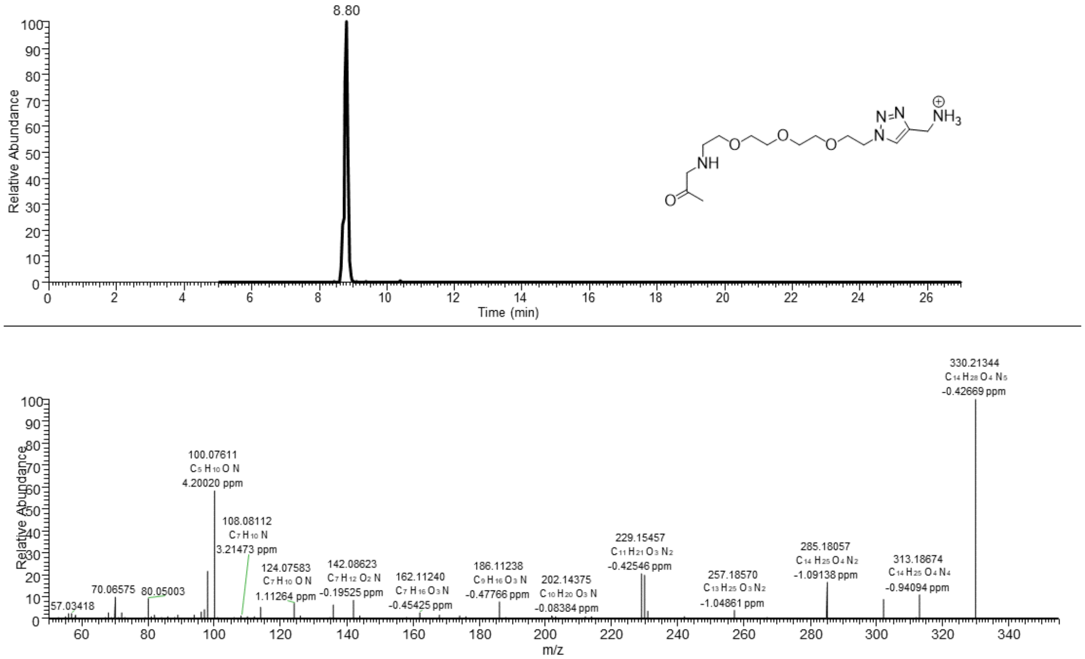

Figure S2.68. Selected ion chromatogram and MS^2 spectrum for the methylglyoxal Schiff base adduct via reduced NH_2-bead derivatization (*m/z* 330).
